# Fungal and ciliate protozoa are the main rumen microbes associated with methane emissions in dairy cattle

**DOI:** 10.1093/gigascience/giab088

**Published:** 2022-01-25

**Authors:** Adrián López-García, Alejandro Saborío-Montero, Mónica Gutiérrez-Rivas, Raquel Atxaerandio, Idoia Goiri, Aser García-Rodríguez, Jose A Jiménez-Montero, Carmen González, Javier Tamames, Fernando Puente-Sánchez, Magdalena Serrano, Rafael Carrasco, Cristina Óvilo, Oscar González-Recio

**Affiliations:** Departamento de Mejora Genética Animal, Instituto Nacional de Investigación y Tecnología Agraria y Alimentaria, Crta. de la Coruña km 7.5, 28040 Madrid, Spain; Departamento de Mejora Genética Animal, Instituto Nacional de Investigación y Tecnología Agraria y Alimentaria, Crta. de la Coruña km 7.5, 28040 Madrid, Spain; Escuela de Zootecnia y Centro de Investigación en Nutrición Animal, Universidad de Costa Rica, 11501 San José, Costa Rica; Departamento de Mejora Genética Animal, Instituto Nacional de Investigación y Tecnología Agraria y Alimentaria, Crta. de la Coruña km 7.5, 28040 Madrid, Spain; NEIKER – Instituto Vasco de Investigación y Desarrollo Agrario. Basque Research and Technology Alliance (BRTA), Campus Agroalimentario de Arkaute s/n, 01192 Arkaute, Spain; NEIKER – Instituto Vasco de Investigación y Desarrollo Agrario. Basque Research and Technology Alliance (BRTA), Campus Agroalimentario de Arkaute s/n, 01192 Arkaute, Spain; NEIKER – Instituto Vasco de Investigación y Desarrollo Agrario. Basque Research and Technology Alliance (BRTA), Campus Agroalimentario de Arkaute s/n, 01192 Arkaute, Spain; Confederación de Asociaciones de Frisona Española (CONAFE), Ctra. de Andalucía km 23600 Valdemoro, 28340 Madrid, Spain; Departamento de Mejora Genética Animal, Instituto Nacional de Investigación y Tecnología Agraria y Alimentaria, Crta. de la Coruña km 7.5, 28040 Madrid, Spain; Departamento de Biología de Sistemas, Centro Nacional de Biotecnología, CSIC, Madrid, 28049 Madrid, Spain; Departamento de Biología de Sistemas, Centro Nacional de Biotecnología, CSIC, Madrid, 28049 Madrid, Spain; Departamento de Mejora Genética Animal, Instituto Nacional de Investigación y Tecnología Agraria y Alimentaria, Crta. de la Coruña km 7.5, 28040 Madrid, Spain; Departamento de Periodismo y Nuevos Medios, Universidad Complutense de Madrid, Ciudad Universitaria s/n, 28040 Madrid, Spain; Departamento de Mejora Genética Animal, Instituto Nacional de Investigación y Tecnología Agraria y Alimentaria, Crta. de la Coruña km 7.5, 28040 Madrid, Spain; Departamento de Mejora Genética Animal, Instituto Nacional de Investigación y Tecnología Agraria y Alimentaria, Crta. de la Coruña km 7.5, 28040 Madrid, Spain; Departamento de Producción Agraria, Escuela Técnica Superior de Ingeniería Agronómica, Alimentaria y de Biosistemas, Universidad Politécnica de Madrid, Ciudad Universitaria s/n, 28040 Madrid, Spain

**Keywords:** dairy cattle, microbiome, rumen, methane, Nanopore, long reads

## Abstract

**Background:**

Mitigating the effects of global warming has become the main challenge for humanity in recent decades. Livestock farming contributes to greenhouse gas emissions, with an important output of methane from enteric fermentation processes, mostly in ruminants. Because ruminal microbiota is directly involved in digestive fermentation processes and methane biosynthesis, understanding the ecological relationships between rumen microorganisms and their active metabolic pathways is essential for reducing emissions. This study analysed whole rumen metagenome using long reads and considering its compositional nature in order to disentangle the role of rumen microbes in methane emissions.

**Results:**

The β-diversity analyses suggested a subtle association between methane production and overall microbiota composition (0.01 < *R*^2^ < 0.02). Differential abundance analysis identified 36 genera and 279 KEGGs as significantly associated with methane production (*P*_adj_ < 0.05). Those genera associated with high methane production were Eukaryota from Alveolata and Fungi clades, while Bacteria were associated with low methane emissions. The genus-level association network showed 2 clusters grouping Eukaryota and Bacteria, respectively. Regarding microbial gene functions, 41 KEGGs were found to be differentially abundant between low- and high-emission animals and were mainly involved in metabolic pathways. No KEGGs included in the methane metabolism pathway (ko00680) were detected as associated with high methane emissions. The KEGG network showed 3 clusters grouping KEGGs associated with high emissions, low emissions, and not differentially abundant in either. A deeper analysis of the differentially abundant KEGGs revealed that genes related with anaerobic respiration through nitrate degradation were more abundant in low-emission animals.

**Conclusions:**

Methane emissions are largely associated with the relative abundance of ciliates and fungi. The role of nitrate electron acceptors can be particularly important because this respiration mechanism directly competes with methanogenesis. Whole metagenome sequencing is necessary to jointly consider the relative abundance of Bacteria, Archaea, and Eukaryota in the statistical analyses. Nutritional and genetic strategies to reduce CH_4_ emissions should focus on reducing the relative abundance of Alveolata and Fungi in the rumen. This experiment has generated the largest ONT ruminal metagenomic dataset currently available.

## Introduction

Next-generation sequencing technologies have provided special relevance to microbial communities from different niches because they let their taxonomic and functional profile be identified. This has made it possible to unravel the relationships between host and microbiota, as well as the complex interactions between microbes, focusing on the special contribution of the role of digestive microbiome in complex traits both in humans [1] (e.g., Type 2 diabetes, cancer, mental diseases) and in domestic animals [2,3] (e.g., feed efficiency, methane emissions, animal health).

Microbial communities are of special relevance in livestock. In ruminants, one of the main microbial communities is found in the rumen, owing to its high diversity and large microbial mass [4] and its main role in feed fermentation to provide substrate to the animal, which is then transformed into product. Additionally, enteric methane is produced in the rumen by methanogenic microorganisms during feed fermentation [5] and is the main contributor of greenhouse gases from livestock, with 2.8–3.5 gigatonnes of CO_2_-equivalent per year [6,7]. The ongoing climate emergency urgently calls for efficient strategies to mitigate the carbon footprint from all sectors, including agriculture and livestock farming. Previous studies have proven that complex traits in ruminants are usually influenced by global changes in ruminal microbial communities, more than by fluctuations in the abundance of specific microorganisms [8,9]. These global changes are usually due to the intricate interactions between different species in these communities (i.e., predation, competition of ecological niche, or co-dependency). Consequently, a better understanding of the interactions between microbial genes during methanogenesis is needed to propose strategies for reducing methane emissions. Promising strategies have been proposed to modulate the metagenome, nutrition, and genetics [10].

Classic statistical approaches do not allow the results of microbiome studies to be accurately assessed. The high sparsity of these data and their compositional nature generate multiple problems in statistical analysis, including subcompositional incoherence, increased false-positive rates in differential abundance analyses, and detection of spurious correlations [11].

As a consequence, new approaches considering both compositionality and multiple correlations are needed. It is also important to point out the advantages of whole-metagenome sequencing over metataxonomic studies because the latter cannot be used to determine functionality and because they pose some difficulties at simultaneously analysing different superkingdoms [12], which is necessary to account for the total variability of microbiomes and the interactions among their components. Different amplicons must be used to correctly classify Bacteria, Archaea, Protozoa, and Fungi, increasing the cost of the studies and involving additional bias due to PCR [13]. They pose the additional difficulty of a proper comparison between communities sequenced in different reactions with different primers. Nanopore sequencing offers a cost-efficient sequencing strategy for metagenomics studies, providing both taxonomical and functional information simultaneously and for microbes from all superkingdoms. This technology has been improved in recent years, allowing taxonomic and functional assignments to be performed with an accuracy comparable to Illumina [14].

The objective of this study was to characterize the taxonomical and functional composition of rumen microbiota using long sequence reads obtained with Nanopore technology, and their relationship with enteric methane emission.

## Results

### Taxonomy of microbial composition

After initial selection of core taxonomy, 6,394,671 reads with N50 = 4,022 bp were classified in 3,921 taxonomical features up to genus level. A filtering strategy was implemented to exclude low-abundance microbes while keeping the core microbiome relevant for methane emissions. This process removed 48,517 reads (<1%), which reduced the sparsity of the metagenome from 87% to 68%, although a large number of singleton and doubleton features remained (Supplementary Fig. S1). The final core subcomposition included a total of 6,318,344 reads, in 437 samples, classified in 1,240 taxonomical features: 967 known genera (722 bacteria, 13 archaea, and 232 eukaryotes) and 273 that only reached family rank (i.e., Unclassified denomination). Overall, 503 families, 277 orders, 158 classes, and 86 different phyla (37 bacterial phyla, 3 archaeal phyla, and 46 eukaryotic clades) were classified. The relative abundance (RA) distribution by superkingdoms and phyla is summarized below.

**Figure 1:**
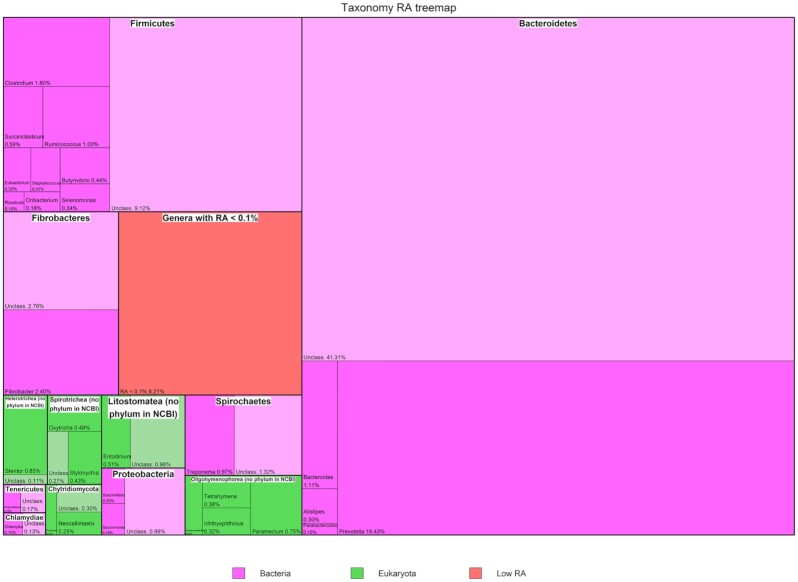
Mean relative abundance of genera. Mean relative abundance of core microbial taxa, including those classified only to family level (i.e., unclassified genera), which represent 60.2% of total abundance.

Predominant microorganisms in this core rumen subcomposition were bacteria (mean 91.6% [SD 6.93] of total mean RA) from Bacteroidetes, Firmicutes, and Fibrobacteres (Fig. 1), representing a mean RA of 63%, 16%, and 5%, respectively. The Bacteroidetes fraction was majorly composed by *Prevotella* and was the main representative genus in the total community (19.4% mean RA), along with other Prevotellaceae members. The Firmicutes group included a large number of genera. The order of Clostridiales dominated in terms of RA, with Lachnospiraceae and Ruminococcaceae families being the most representative ones. The remaining phyla (34) from the Bacteria superkingdom represented 7.6% mean RA of the core metagenome. Eukaryotes represented a total mean RA of 8.2% (SD 6.95) of the core subcomposition. Predominant eukaryotic clades were those included in the SAR supergroup (Stramenopiles-Alveolata-Rhizaria) [15], accounting for 6% of total mean RA, followed by Fungi (1.3% of total mean RA). Alveolata clade was the most abundant among the eukaryotes, with a high representation of unclassified Ophryoscolecidae, *Stentor*, and *Paramecium*. Archaea representation in the core subcomposition (mean 0.24% [SD 0.25] of total mean RA) consisted mostly of Methanomicrobia, Methanobacteria, and Thermoplasmata members. Yet, a large number of reads could not be assigned to a known genus. The relative abundance per animal of the most relevant taxonomic groups is depicted in Supplementary Fig. S2.

### Functionality of microbial composition

**Figure 2:**
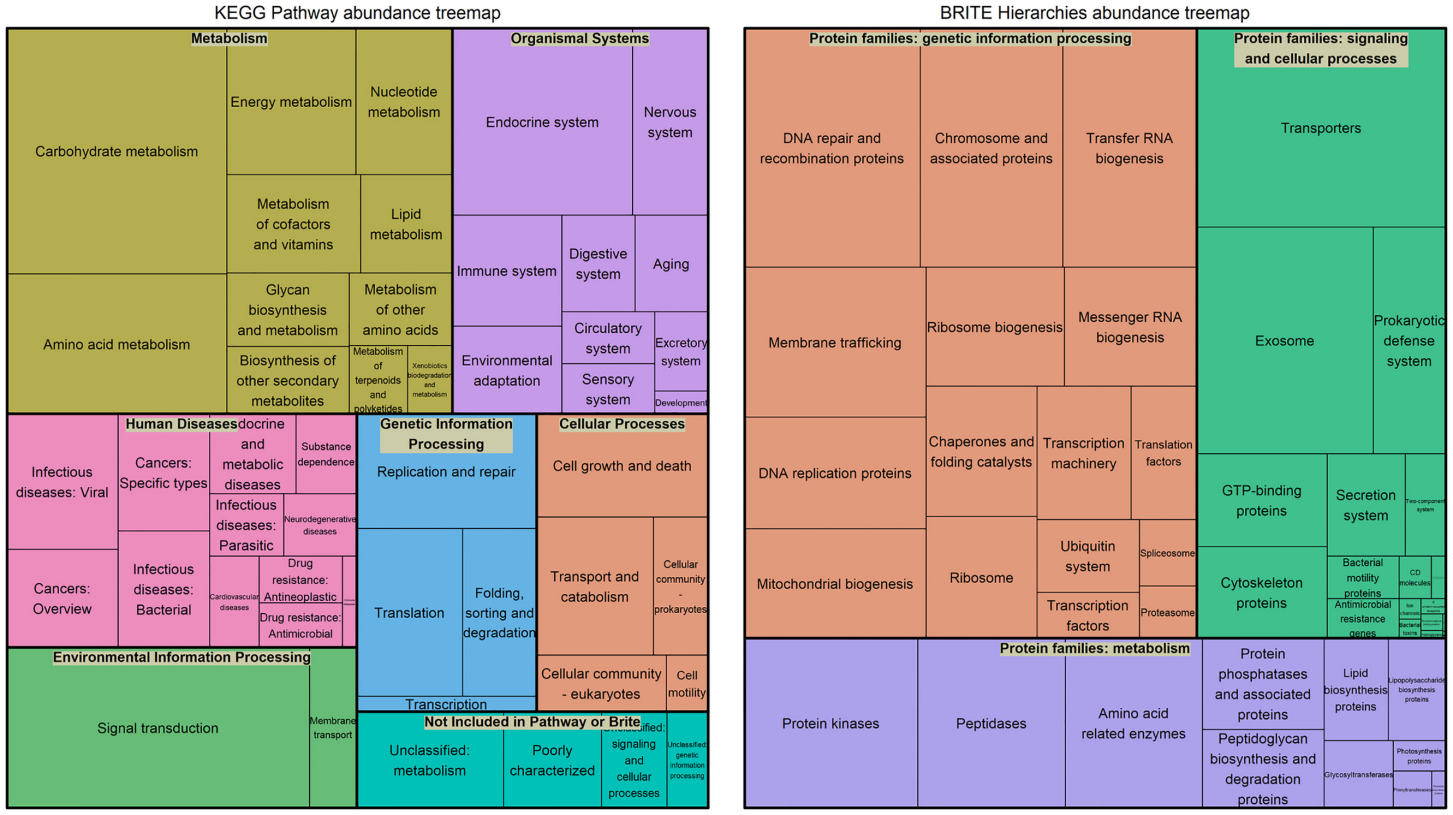
Metagenome functionality. TreeMap distribution of functionality abundances classified as KEGG pathways (left) and BRITE hierarchies (right) associated with core KEGG subcomposition.

A total of 30,326,550 reads with N50 = 5,720 bp were assigned to KEGGs. After prevalence filtering, a total of 84,219 reads (0.28%) were removed and the sparsity was reduced from 72% to 39% (Supplementary Fig. S1). The final KEGG table was composed by 30,145,459 reads from 437 samples, classified in 6,644 KEGGs. These KEGG pathways and BRITE hierarchies [16–18] were represented in a Treemap according to their mean RA (Fig. 2). We found that 26% of the rumen metagenome functions were in pathways that represent the metabolism of carbohydrates, amino acids, and other biological compounds, as well as of energy metabolism. In addition, 34% of functions were involved in cellular generic processes (cell growth [3%], transport and catabolism [4%], genetic and environmental information processing [23%], and other [4%]). KEGG BRITE classification showed a high presence of proteins involved in cellular processes (36%) and metabolism (26%).

### β-diversity and PERMANOVA analysis

**Figure 3:**
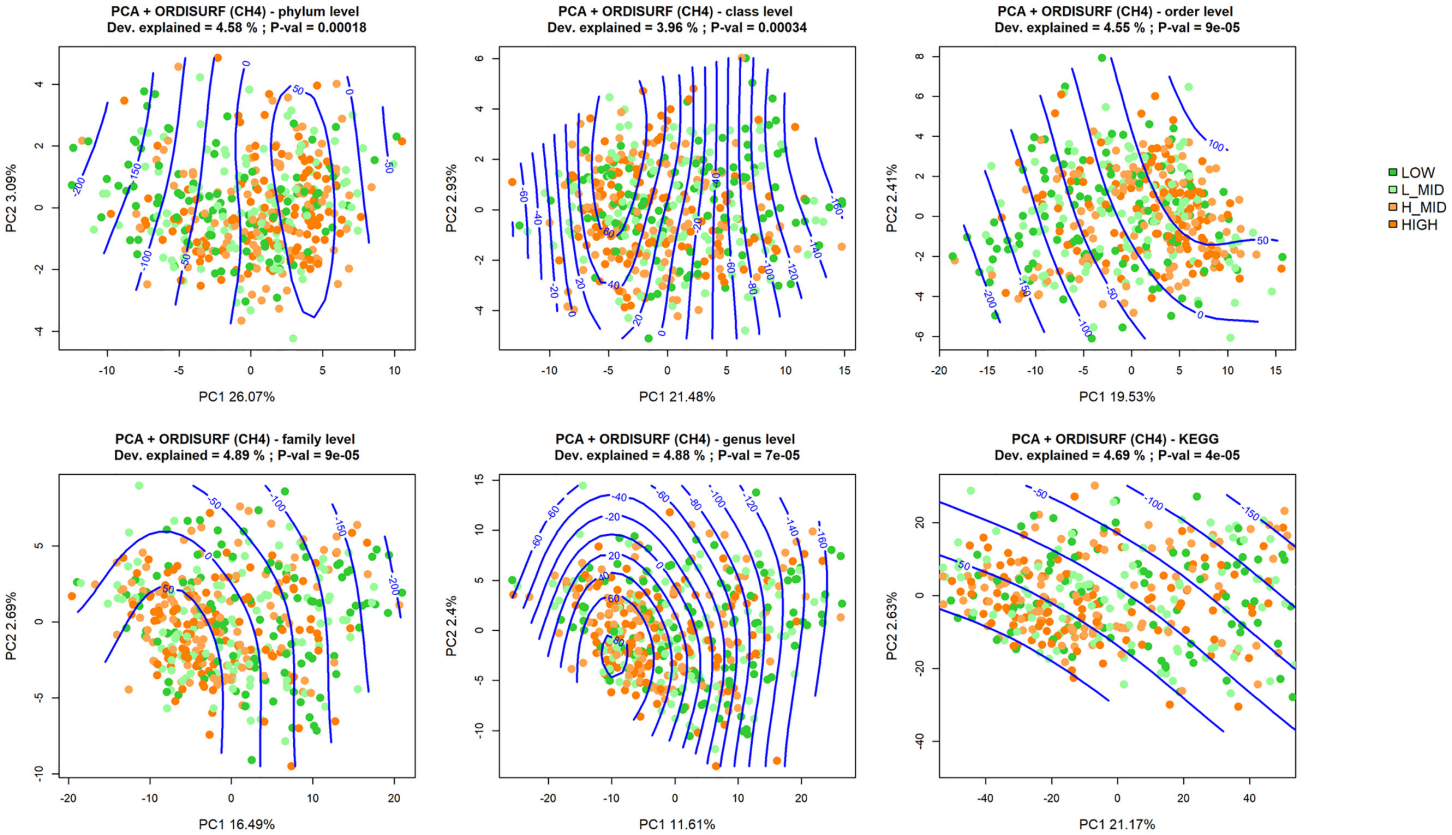
Fitted surface representation of principal component analysis. Dots represent the samples using Euclidean distances of CLR-transformed taxa abundances, coloured by CH4 levels. CH_4_ emissions (ppm) corrected by number and stage of lactation are represented as smooth fitting following a generalized additive model (GAM) (green). Dev. Explained: variability explained by GAM; P-val: approximate significance of the smooth terms being 0 (α = 0.05).

The β-diversity was represented in principal component analysis (PCA) between samples at 5 different taxonomic levels (phylum, class, order, family, and genus), as well as with KEGG, using centered log-ratio (CLR) transformed datasets. Then a permutational analysis of variance (PERMANOVA) was implemented [11], sequentially adding the effect of farm-batch (B), stage of lactation (SL), number of lactation (NL) and level of methane emissions (CH4) discretized in 4 groups (LOW, L-MID, H-MID, and HIGH). The visualization did not show a clear visual clustering of samples by methane emission levels (Fig. 3). However, a generalized additive model (GAM) smooth fitting allowed visualization of non-linear distribution patterns of the microbial samples according to CH_4_ emissions inside the ordination at all taxonomic levels. The non-linear pattern was more evident at the phylum, class, and genus levels, although the proportion of methane variability explained was low (≃4.8% according to GAM model fitting). No relevant differences were visually appreciated using the KEGG information. Nonetheless, some differences in the overall rumen microbiome composition between animals with different methane emissions were evidenced by the PERMANOVA analysis, both for taxonomy and functionality (Table 1). The results showed significant differences for the centroid distance between methane emission groups at every taxonomic level and also for KEGGs (*P* < 0.01), but they explained a low percentage of total variance (0.01 < *R*^2^ < 0.02).

**Table 1:** F statistic and *P*-values for stage of lactation (SL), number of lactation (NL), and methane emission (CH4) variables (added sequentially) and *P*-values from PERMANOVA of the entire dataset (i.e., including all superkingdoms)

Category	Variable	F statistic	*R* ^2^	*P*-value*
Phylum	SL	6.1	0.014	*<0.01*
	NL	1.4	0.003	0.11
	CH4	2.8	0.019	*<0.01*
Class	SL	5.6	0.013	*<0.01*
	NL	1.5	0.003	0.07
	CH4	2.4	0.016	*<0.01*
Order	SL	5.4	0.012	*<0.01*
	NL	1.7	0.004	*0.03*
	CH4	2.3	0.016	*<0.01*
Family	SL	4.9	0.011	*<0.01*
	NL	1.6	0.004	*0.03*
	CH4	2.1	0.014	*<0.01*
Genus	SL	4.0	0.009	*<0.01*
	NL	1.4	0.003	*0.03*
	CH4	1.7	0.012	*<0.01*
KEGG	SL	5.3	0.012	*<0.01*
	NL	2.0	0.004	*0.02*
	CH4	2.4	0.016	*<0.01*

* Italics indicate a statistically significant finding (*P* < 0.05).

### Rumen microbes associated with CH_4_ emissions

**Figure 4:**
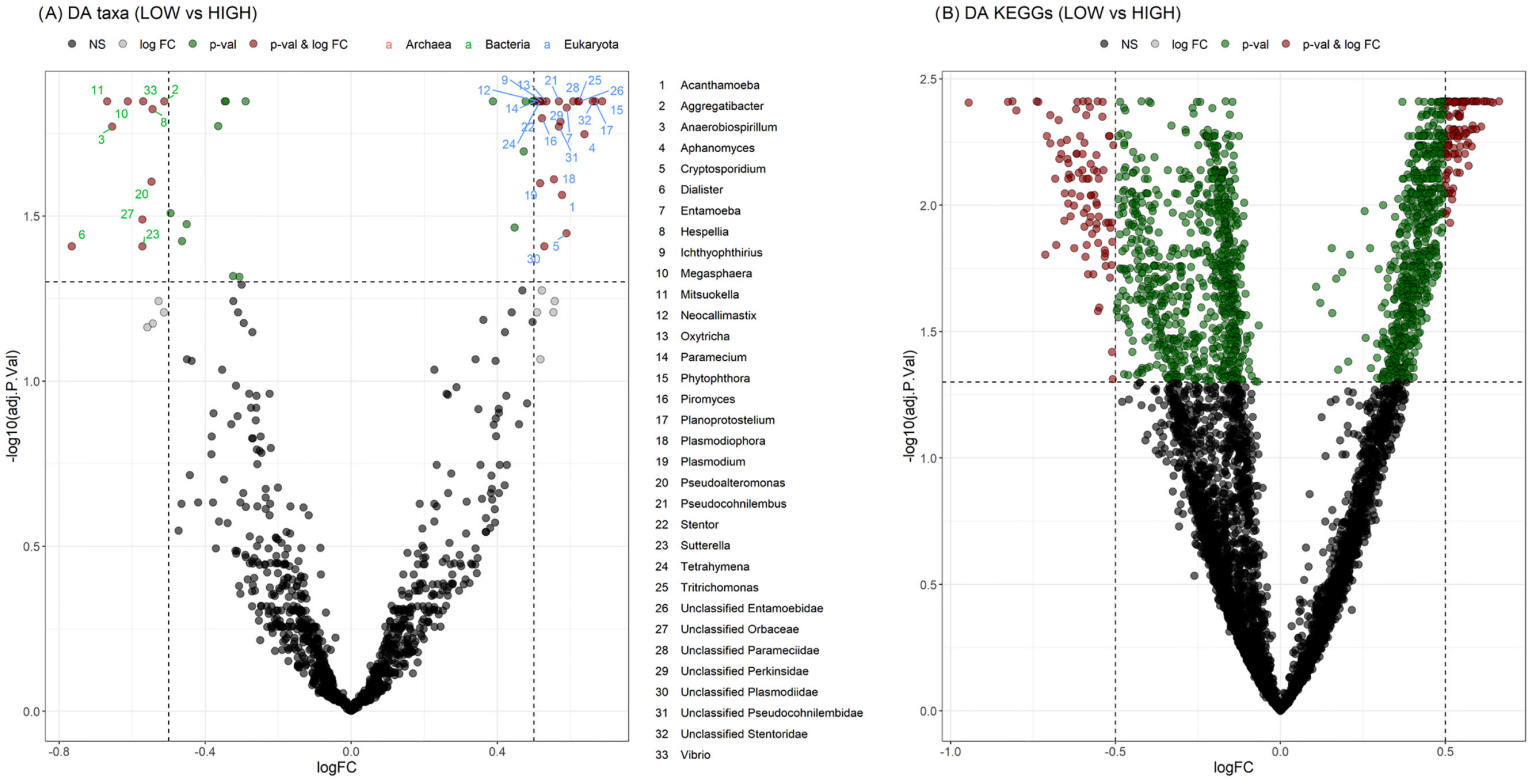
Volcano plots. Volcano plot representing the differential abundance (DA) of genera (A) and KEGGs (B) between LOW and HIGH groups from limma. Significance thresholds were established at *P*_adj_ = 0.05 and log_2_FC = ±0.5. Red points: Significant features with DA above the fold change (FC) threshold. Green points: Significant features with DA below the FC threshold. Gray points: Non-significant features with DA above the FC threshold. Black points: Non-significant features with DA below the FC threshol.

The effect of taxonomic features on methane emission levels was evaluated through differential abundance (DA) analysis. Thirty-three genera were found to be differentially abundant (*P*_adj_ <0.05) between LOW and HIGH emitters (Fig. 4A), while 15 genera showed DA between LOW and H-MID emitters and 1 genus between LOW and L-MID emitters (Supplementary Data S1). Note that 13 of the 15 genera showing DA (*P*_adj_ <0.05) between LOW and H-MID groups were also significant in the LOW vs HIGH contrast but not in LOW vs L-MID contrast, indicating gradual abundance change from low to high emitters. Accounting for all contrasts and duplicated genera, 36 DA genera had statistically significant results. We classified these genera according to their respective overabundance (OA) in the LOW or HIGH emissions groups. Thus, 10 of them were more abundant in the LOW group (LOW-OA) and 1 in the L-MID group. The remaining 25 genera were OA in the HIGH groups (HIGH-OA): HIGH (12), HIGH and H-MID (11), or H-MID (2). HIGH-OA genera represented an overall RA of 4.15%, whereas LOW-OA genera accounted for 0.25% of total RA. The 2 genera overabundant in H-MID were *Dictyostelium* and *Unclassified Eimeriidae*, and the one associated to L-MID was classified as *Candidatus Izimaplasma* (Tenericutes). The log_2_FC values ranged between 0.7 and −0.7 in genera showing DA for methane emission levels, highlighting that the differences between groups were moderate.

Overall, DA results indicate that taxa associated with higher methane levels belong to the Eukaryota superkingdom, while those associated with lower emissions were bacteria. We found multiple Ciliophora genera associated with the HIGH group (mostly Parameciidae, Stentoridae, and Pseudocohnilembidae members) but also Amoebozoa and some Fungi or pseudo-fungi. Other bacterial genera associated with lower methane production were *Hespellia*, from Clostridiales, and *Sutterella*, an asaccharolytic genus from Betaproteobacteria.

### Microbial gene function associated with CH_4_ emissions

DA analysis was also performed for KEGG features on methane emission levels. A total of 192 KEGGs were DA between the LOW and HIGH emissions groups (Fig. 4B). Differences were also found between the LOW and H-MID groups (Supplementary Data S1). As in the taxonomy dataset, some of the KEGGs presented significant DA in both LOW vs HIGH and LOW vs H-MID contrasts. Accounting for these duplicates and all the contrasts, 182 were overabundant in the high-emissions groups (HIGH-OA), whereas 97 KEGGs were overabundant in low-emissions groups (LOW-OA). The overall RA for HIGH-OA KEGGs was 2.31% and 0.64% for LOW-OA KEGGs. Of these, 13 HIGH-OA KEGGs and 28 LOW-OA KEGGs were assigned to metabolic pathways. No KEGGs from the ko00680 pathway were found as HIGH-OA. KEGGs related to inositol-phosphate metabolism (K00889, K01110, K18082, and K20279), starch and sucrose metabolism (K01203), or several lipid metabolism pathways were present in the HIGH-OA group. According to LOW-OA KEGGs, some of them were involved in volatile fatty acid metabolism (e.g., K00209 enoyl-[acyl-carrier protein] reductase [EC:1.3.1.9], K01902 succinyl-CoA synthetase alpha subunit [EC:6.2.1.5], and K01682 aconitate hydratase 2 [EC:4.2.1.3]) and the K09251 putrescine aminotransferase (EC:2.6.1.82) related to putrescine and cadaverine degradation to 4-amino-butanoate (GABA) or 2-oxoglutarate. Also, several KEGGs in the LOW-OA group were related to nitrogen metabolism (K00370 and K00371 nitrate reductase subunits [EC:1.7.5.1]), oxidative phosphorylation (K03885 NADH dehydrogenase [EC:1.6.99.3]), and to carbohydrate, lipid, or vitamin metabolism pathways. The ko00680 KEGG K13788 was also overabundant in the LOW emissions group.

### Co-abundance of genera and KEGGs

Interaction networks were built using the previous results in order to visualize the association between taxa and genes using pairwise correlations between features. Pairwise proportionality correlation coefficients (*ρ*_p_) were calculated on the CLR-transformed datasets for phylum, genus, and KEGG features to mitigate the effect of spurious correlations that can potentially surge in compositional data [19].

**Figure 5:**
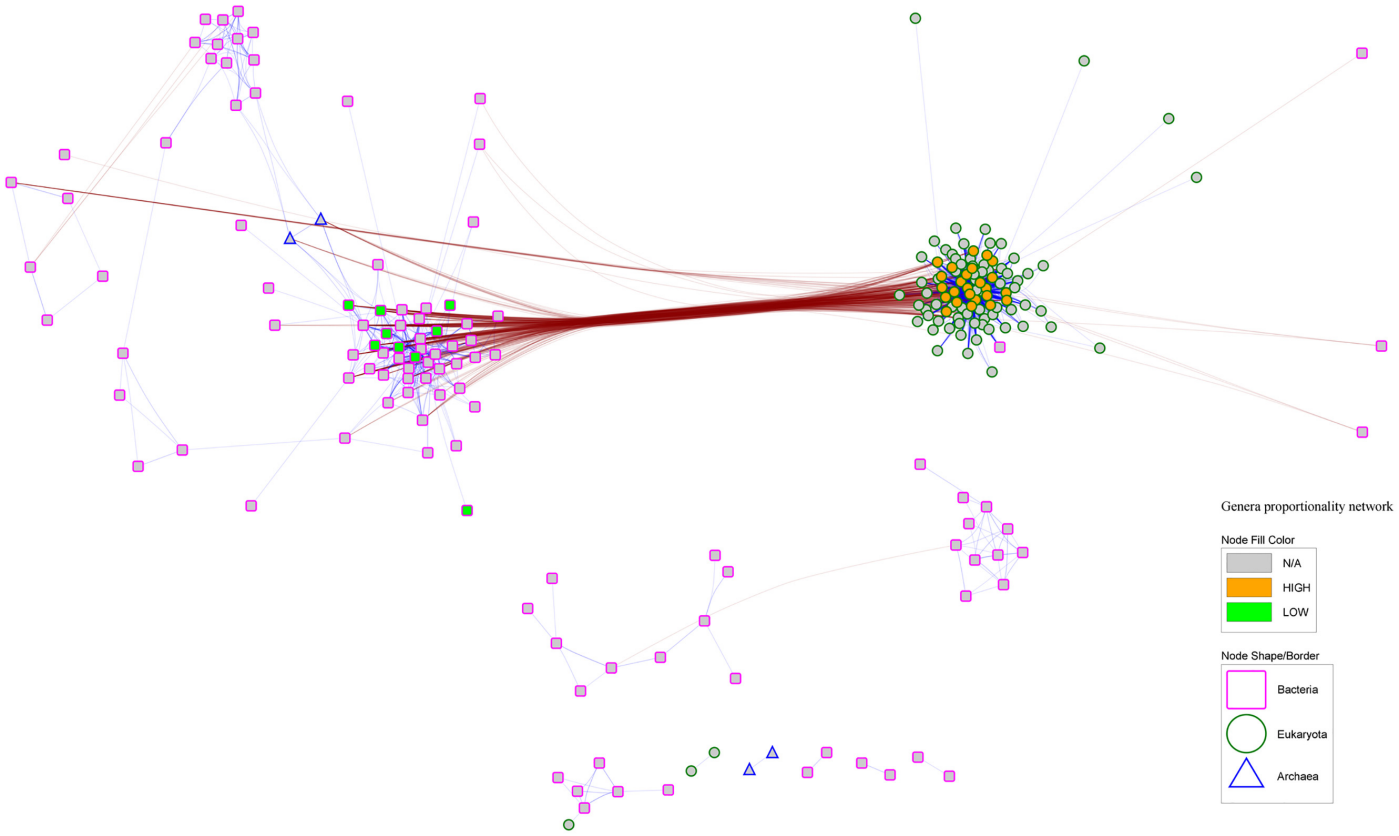
Taxonomy interaction network. Pairwise proportionalities between genera with |ρ_p_| ≥ 0.4. Node shapes and shape colour indicate superkingdom, and node fill colour, CH_4_ association. Blue links indicate direct proportionality (>0), and brown links, inverse proportionality (<0).

**Figure 6:**
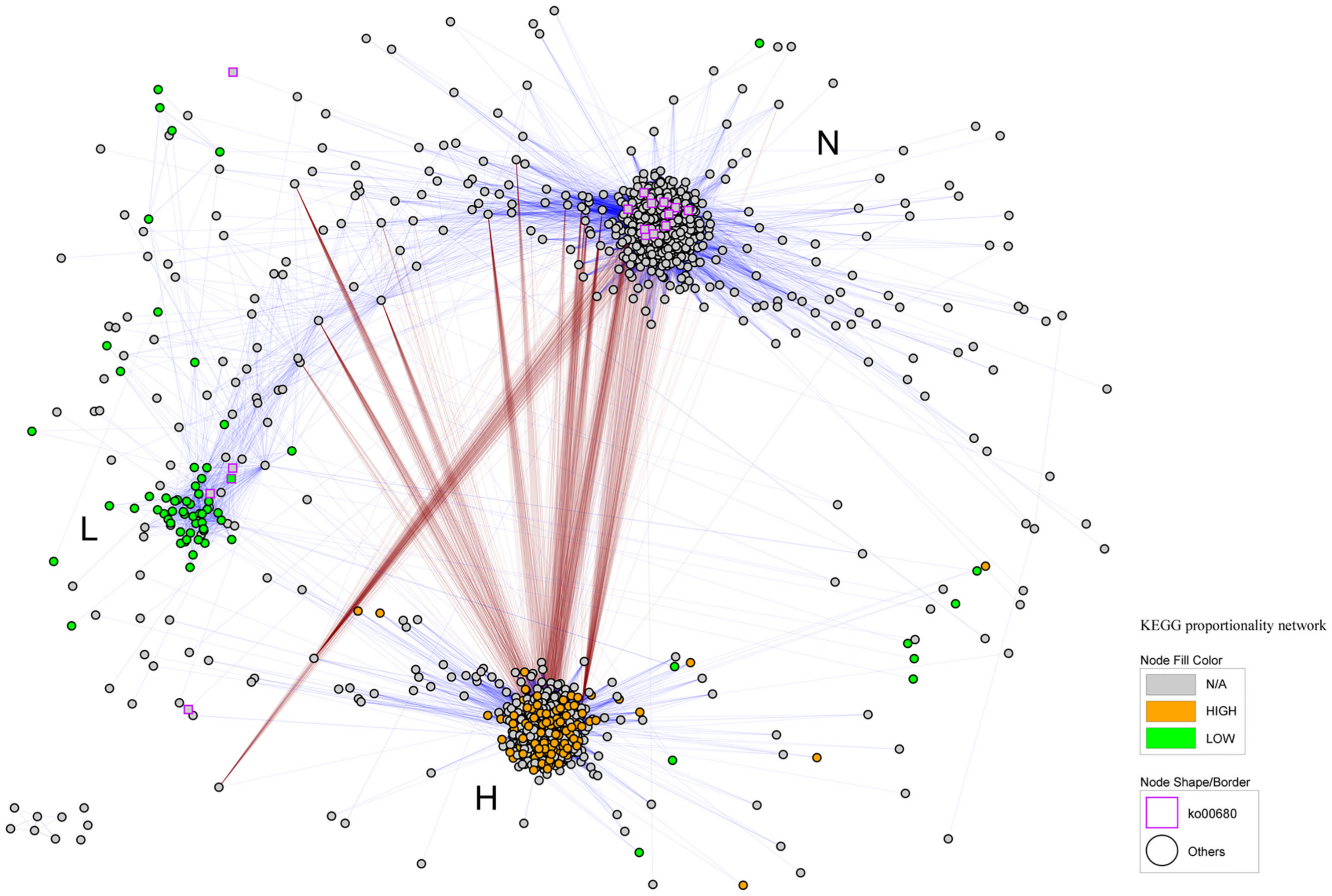
Functionality interaction network. Presented are pairwise proportionalities between KEGGs with |ρ_p_| ≥ 0.7. Node shapes and shape colour indicate participation in methane metabolism (ko00680 [direct or indirect participation] or other [no participation]), and node fill colour, CH_4_ association. Blue links indicate direct proportionality (>0), and brown links, inverse proportionality (<0). Clusters are indicated as L (KEGGs associated with LOW methane), H (KEGGs associated with HIGH methane), and N (KEGGs not related to methane emissions).

The most relevant pairwise proportionalities between genera and between KEGGs were visualized as interaction networks, classifying features as associated with high methane emissions (HIGH), low methane emissions (LOW), or not associated with methane emissions (N/A), according to the results from the differential abundance analyses. The interaction networks for genera and KEGGs are shown in Figs 5 and 6, respectively.

Eukaryotes clustered together in the network with large representation of the SAR supergroup and showed negative proportionality to bacteria. The genera that were associated with higher methane emissions belonged to the Eukaryota superkingdom (Ciliophora and Fungi), whereas Bacteria were associated with lower CH_4_ production. The strongest inverse proportionalities between both subpopulations connected several eukaryotes with Unclassified Veillonellaceae and *Oribacterium* ($- 0.64 < {\rho _p} < \,\, - 0.53$); i.e., microbiomes with lower abundance of *Oribacterium* or Veillonellaceae tend to present larger abundances of protozoa and fungi and were therefore associated with larger emissions. Unclassified microbes from Neocallimastigaceae, Oxytrichidae, and Vibrionaceae families showed the highest centrality and a large degree of connectivity.

The functional network showed 3 main clusters that grouped KEGGs associated with HIGH methane level (cluster H), KEGGs not related to methane emissions (cluster N), and a small one including KEGGs associated with lower emissions (cluster L). Connections between clusters were not symmetric: H cluster was connected to N cluster by inverse proportionalities between some of their components, but the L cluster seemed to be connected only to N cluster by direct proportionalities through non-clustered KEGGs. Also, most of the ko00680 KEGGs (i.e., directly involved in methanogenesis or participating in pathways leading to methanogenesis precursors) did not appear as DA between high-emission and low-emission cows.

### Distribution of genes among clades

**Figure 7:**
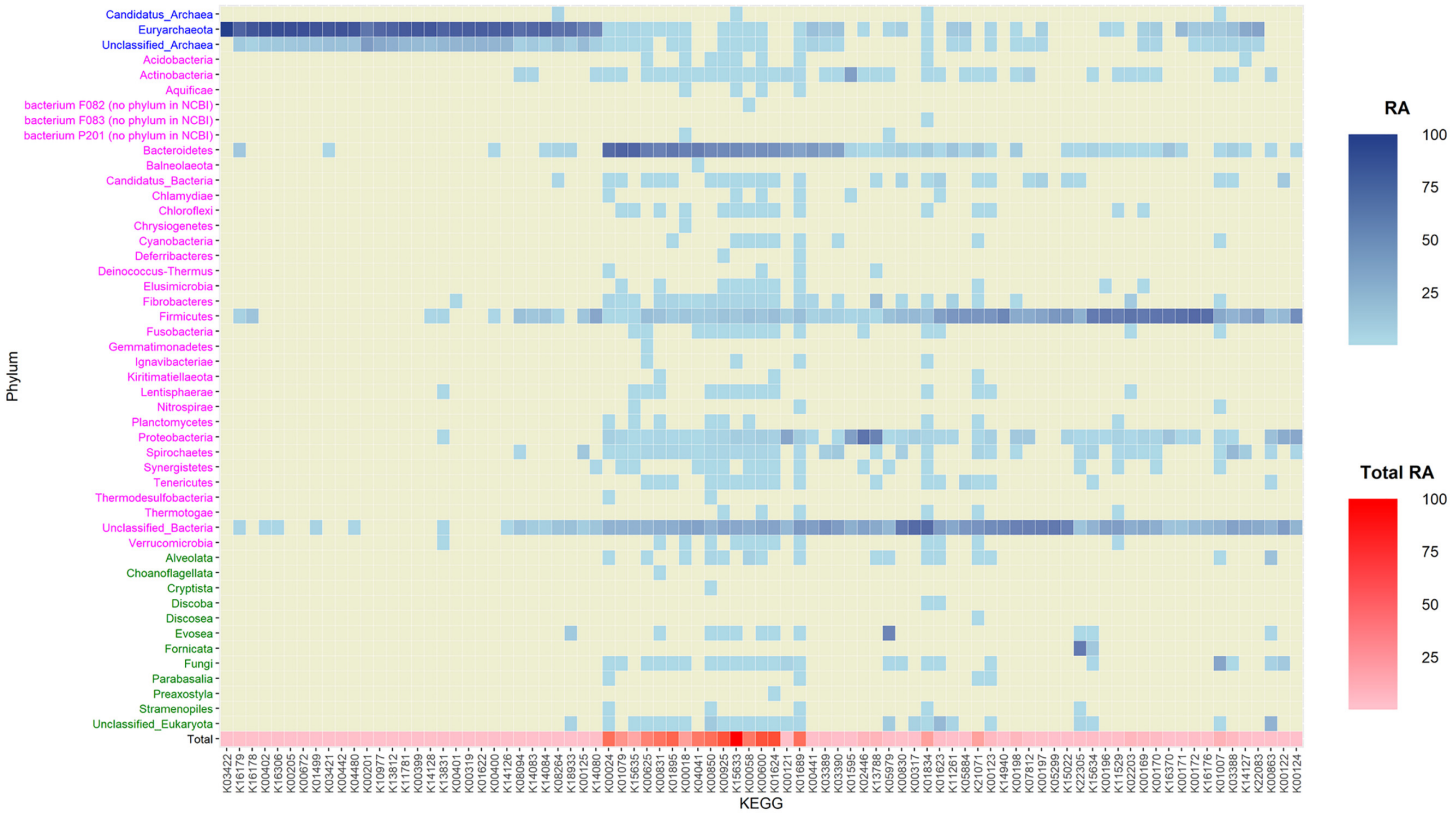
Taxonomy of ko00680 KEGGs. Relative abundance of KEGGs present in ko00680 pathway for each phylum in Archaea (blue), Bacteria (fuschia), and Eukaryota (green) superkingdoms. Relative abundance of each ko00680-KEGG with respect to the sum of reads mapped to all ko00680-KEGGs.

**Figure 8:**
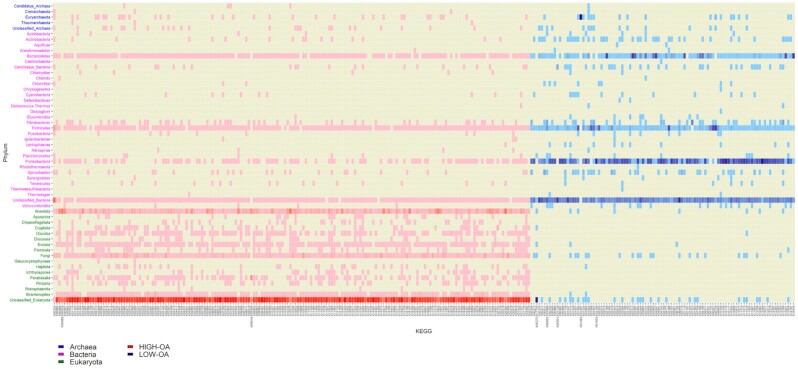
Taxonomic distribution of DA KEGGs. Red density scale represents KEGGs overabundant (OA) in HIGH emitters; Blue density scale represents KEGGs OA in LOW emitters. More intense colours mean a higher number of reads assigned to 1 phylum.

A traceback of genes’ taxonomy was carried out, separately for ko00680 KEGGs and for DA KEGGs. A total of 30 of the 85 ko00680 KEGGs were predominant in Archaea groups, 1 predominated in Eukaryota (K05979), and the rest were predominant in Bacteria (Fig. 7). Although the RA distribution of these KEGGs was normally between 60% and 100% in the predominant superkingdom, 4 KEGGs were more evenly distributed between clades: K01007 and K00863 had RA < 60% in Bacteria and showed RA > 30% in Eukaryota; K05979 was the KEGG predominating in Eukaryota, but with RA near 60% (38% in Bacteria and 12% in Archaea); and K14080 had RA of 57% in Archaea and 43% in Bacteria. Regarding the DA KEGGs, those from the LOW-OA group showed larger abundance in Bacteria, mostly in genera from Proteobacteria, Bacteroidetes, and Firmicutes phyla. Different groups of bacteria also carried KEGGs from the HIGH-OA group, although these KEGGs were more abundant in eukaryotes. The HIGH-OA KEGGs were mainly mapped to unclassified eukaryotes, but those that could be classified belonged most often to Fungi and SAR supergroup (Fig. 8).

## Discussion

In this study we assessed the composition of the ruminal microbiota using long reads from Nanopore sequencing technology. We observed predominance of Bacteroidetes, Firmicutes, and Fibrobacteres, as reported in previous studies [8,20]. Bacteroidetes and Firmicutes are common bacteria in all kind of ecosystems, including gut microbiota of multiple animals. The fraction of Bacteroidetes was mainly composed by *Prevotella*, a group of anaerobic gram-negative bacteria involved in saccharolytic processes [21]. Their large abundance in the digestive microbiota has been previously reported in ruminant [22–26] and monogastric species [27,28]. Firmicutes were less abundant, with a more diverse distribution of genera. Fibrobacteres, a small group of cellulose-degrading bacteria usually present in ruminant digestive systems [29], was mainly represented by the *Fibrobacter* genus. Eukaryotes also represented a relevant amount of the rumen core metagenome. This group has been reported to contribute up to 50% of total ruminal biomass [30]. The SAR supergroup and Fungi were the most relevant ones, which are found in a wide variety of ruminants and pseudoruminants [15,31]. Other eukaryotes included *Stentor* and *Paramecium*; the former are aquatic free-living heterotricheans that can be particle filtrators or predators of other protozoa and live symbiotically with some algae species [32,33], whereas the latter are well-known ciliates that predate bacteria and other microorganisms, including protozoa [34]. Archaeal fraction was mostly composed of strict methanogenic organisms from Methanomicrobia and Methanobacteria clades [35] but also included Thermoplasmata, which are methylotrophic-methanogenic acidophilic organisms [36].

The DA analysis showed that ciliates, fungi, and pseudo-fungi were more abundant in cows with higher levels of methane emissions. Microbes associated with lower methane emissions were saccharolytic members of class Gammaproteobacteria (*Anaerobiospirillum* [37], *Vibrio* [38], or *Pseudoalteromonas* [39]), as well as Negativicutes genera from Veillonellaceae (*Dialister, Megasphaera*) and Selenomonadaceae (*Mitsuokella*). *Dialister* produce succinate decarboxylation, and *Megasphaera* ferment carbohydrate and lactate [40], while *Mitsuokella* are saccharolytic bacteria [41]. The low-emissions ruminotype had larger abundance of Proteobacteria and Firmicutes genera. Other authors also reported higher abundances of these bacterial phyla in low methane emissions animals [8]. Lactate and succinate producers have been reported to be more abundant in low-emitters as well [42], supporting the higher abundance of *Anaerobiospirillum* or *Megasphaera* in LOW animals.

Despite this association between methane and large taxonomic groups, it is of interest to find out which specific clades and microbial genes are participating directly or indirectly in methanogenesis. The genera co-abundance network showed a clear cluster of eukaryotes, with many of them being significantly more abundant in the high-emissions group. Other authors have already established a positive correlation between fungi abundance and methane emissions [8], as well as a close interdependence of protists and fungi. Although correlation between methane emissions and protozoa abundances is still under discussion [43,44], current meta-analyses point to a linear relationship between protozoa abundance and methane emissions (*r* = 0.96) [45].

Interestingly, no taxonomic group of methanogenic archaea showed association with methane emissions. The relationship between Archaea and methane production in rumen is not consistent in the literature. Some authors reported either individual relationships between methane emissions and some archaeal species [46,47] or correlations between overall archaeal gene abundance and methane emissions level [43,48]. However, other studies showed no relationship between methanogenic Archaea and methane [47,49]. All studies to date showed a low relative abundance of archaea in the rumen, compared to eukaryotes and bacteria [50]. However, the association between the abundance of rumen eukaryotes and methane emissions has been demonstrated through defaunation experiments, both *in vitro*[51,52] and *in vivo* [44,53], with lower emissions in defaunated animals [54]. This has been attributed to the tight link existing between methanogenic archaea abundance and some fungi and protozoa [50]. Specifically, ciliates and some Chytridiomycota (e.g., *Neocallimastix* sp.) are known to symbiotically engulf a variety of methanogenic archaea. They provide the archaea with substrate for methane production from H_2_ produced in their hydrogenosomes, as well as protection against oxygen toxicity [30,55,56]. Thus, free-living methanogens might represent a low fraction of microbial population [45], and CH_4_ biosynthesis might be more influenced by endosymbiotic methanogens [55]. Hence, a larger methanogenesis activity is expected to be correlated with a larger abundance of eukaryotes, especially ciliates, which are more abundant and better represented. Another partial explanation for the low abundance of free archaea, and thereby for the lack of association between Archaea and methane emissions in previous studies [10], is that lysis of archaea cell walls often requires specific protocols during DNA extraction, and they might be under-represented in metagenomics studies [57].

In terms of Gene Ontology, the KEGGs were associated with several metabolic functions and cellular processes (nutrient metabolism and biosynthesis, cellular transport, cell growth, or genetic information processing). Pathways related to pathogenic activity were also found, in agreement with the RA of several genera that include known pathogenic species (e.g., *Vibrio, Haemophilus, Trypanosoma*, or *Staphylococcus*), although not every species from these genera is pathogenic, but opportunistic or commensal organisms. Besides, pathogenic activity presence in our dataset might be biased owing to a larger representation of human-related diseases in the databases. The KEGGs were classified according to their presence or absence in ko00680 pathway (methane metabolism), as a way to evaluate their direct involvement in methanogenesis or an indirect involvement in pathways leading to biosynthesis of precursor compounds. Although we found several ko00680 KEGGs, which are presumably involved in the biosynthesis of methanogenesis precursors, most of them were not associated with methane emissions (i.e., not differentially abundant between methane groups). Most of these KEGGs were mainly present in bacteria or eukaryotes and might be functioning in metabolic pathways not related to methanogenesis. For instance, some of the KEGGs inside the methane metabolism pathway can also be involved in glycine, serine, and threonine metabolism (e.g., K00058, K00831, K01079, and K00600), pyruvate and propanoate metabolism (e.g., K00625 and K13788), glycolysis (e.g., K01689, K15633, K01624, and K02446), or anaerobic carbon fixation (e.g., K00198) [16–18]. Another group of ko00680 KEGGs is exclusive from Archaea, but the under-representation of this clade in our dataset might obscure statistical significance.

Other detected KEGGs could be indirectly related to methanogenesis through biosynthesis of precursor compounds. For instance, K00209 and K13788 are involved in butyrate and propanoate biosynthesis, being essentially carried by primary fermentative bacteria [58]. Then the volatile fatty acids can be used by secondary fermenters to produce methanogenesis precursors such as H_2_, CO_2_, acetate, and formate [59,60]. In fact, K13788 is a phosphate acetyltransferase (EC:2.3.1.8) that can be involved in the biosynthesis of acetate from acetyl-CoA [61]. Also, K09251 is involved in biosynthesis of GABA and 2-oxoglutarate. GABA has been related to a volatile fatty acid concentration increment [62], while 2-oxoacid compounds can be used by Archaea to synthesize coenzyme M and coenzyme B, which are essential in methane production [63]. However, all these KEGGs were observed as overabundant in the LOW methane group, suggesting a strong presence of fermentative bacteria in these animals, not directly correlated with methane production.

Other KEGGs that were overabundant in LOW emitters might offer an explanation of the lower presence of active methanogenesis processes through competence mechanisms (e.g., LOW-OA KEGGs K01682, K01902, and K13788 are involved in citrate cycle and pyruvate metabolism, related to respiration). The K00370 and K00371 are nitrate oxidoreductase subunits playing a role in anaerobic respiration using nitrate as electron acceptor. This enzyme uses nitrate as electron acceptor, a process that has been reported as a competitive inhibitor of methanogenesis [64,65]. Nitrate supplementation has proven to be an useful strategy to mitigate methane emissions [66]. Nitrite produced by the nitrate-reductases has a known antimicrobial effect and toxicity to animal cells [67–69], which might also reduce the proportion of free archaea in LOW animals, although toxicity to archaea must be further studied [70]. However, the role of ciliates and fungi must be clarified because their abundance is also lower in LOW emitters. We hypothesize that the predatory nature of these eukaryotes might be a control mechanism for bacterial populations, and their lower relative abundance in LOW animals might allow overgrowth of related bacteria. Nevertheless, there is the possibility that a higher proportion of facultative anaerobes using nitrate as acceptor might affect ciliate populations by toxicity, thus reducing the presence of endosymbiotic methanogenic archaea.

The SqueezeMeta software [71] uses a last common ancestor (LCA) algorithm, which assigns to 1 read the lowest-level taxon common to all hits, using a stringent cut-off identity value for each taxonomic rank. On its part, functional assignments are done with the fun3 algorithm, which by default assigns the hit with the highest mean bitscore compared to the n first hits passing the e-value, identity, and coverage filters. This LCA approach ensures that reads have a large probability of being correctly classified, at the expense of a large number of reads remaining unclassified, which explains the larger number of reads assigned to a known KEGG than to taxa. Despite this strict requirement, this composition is consistent with other populations reported before [2,3,20]. Most studies to date report large abundance of Bacteroidetes and Firmicutes, with *Prevotella spp*. as the most prevalent genus. Some minor discrepancies with other studies were observed in the RA of the core subcomposition. For example, Wallace *et al*. [20] showed a higher presence of Proteobacteria and Euryarchaeota, although using amplicons instead of whole-metagenome sequencing.

Our statistical approach evidenced the difficulty of inferring a phenotypic association between microbiome composition and methane production, with an important role of environmental factors that mask the statistical signal. However, a meaningful relationship between the microbiome composition and methane emissions could be uncovered yet, emphasizing the role of the different phyla, with the Eukaryota superkingdom being of particular relevance. Previous studies also revealed a link between ruminal microbiota and methane production. Difford *et al*. [3] showed different clusters of high and low methane emitters according to their bacterial and archaeal subcomposition. Danielsson *et al*. [46] also found clustering for low and high methane emitters within prokaryotic rumen subcompositions. Wallace *et al*. [20] found that a core set of rumen microbiome was capable of explaining up to 30% of methane emissions variability, mostly formed by prokaryotes. The aforementioned studies used different methodologies, like amplicon analysis and operational taxonomic unit clustering, contrasting with our full-metagenome genus-clustering protocol, which increases the information entropy. Stewart *et al*. [72] used Nanopore sequencing and found significant differences between low and high methane emitter sheep, with clear clustering between groups, but using a lower number of microbial groups and animals in the same farm with similar management practices.

## Conclusions

The full metagenome compositional analysis used in this study provided novel insights in the association between the microbiota and CH_4_ emissions through differential abundance analysis, pairwise correlation, and interaction networks. Our approach evidenced a phenotypic association between microbiome composition and methane production, regardless of the challenges posed by the microbiome complexity and the compositional nature of the data. This association is mainly driven by the relative abundance of ciliates and fungi, which carry host-specific genetic functions providing substrate to the methanogenic archaea. On the other side, we detected some bacterial groups that performed a more efficient feed digestion, leaving less hydrogen available to archaea and hence associated with lower methane emissions.

This study generated the largest ruminal metagenomic dataset sequenced using ONT and grants free access to a publicly available dataset. The complexity of the rumen microbiome and the compositional nature of their sequencing data require proper statistical methods to allow disentangling the role of microbes and their genes in host complex traits such as methane emissions. Future nutritional and genetic strategies to reduce CH_4_ emissions should focus on reducing the relative abundance of Alveolata and Fungi in the rumen, without impairing other important metabolic processes for an efficient feed digestion in ruminants.

## Methods

### Animal housing and feeding

Our cohort included 439 Holstein lactating cows sampled at 14 different herds from northern Spain (Cantabria, Euskadi, Navarra, and Girona regions). The animals received total mixed ration diet differently formulated for each individual herd, although most of them were based on maize and grass silage plus concentrate. Cows were fed *ad libitum*, with concentrate supplementation in the automatic milking station (AMS) during milking.

### Methane measurement

Methane concentration was individually recorded through breath sampling during each cow visit to the AMS (3–7 times daily) in a period of 2–3 weeks. Eructation peaks were recorded using a non-dispersive infrared methane detector (Guardian NG infrared gas monitor, Edinburgh Sensors, Livingston, UK) as described by Rey *et al*. [73]. Each cow's peaks were then averaged to get a unique methane record per cow, as described by López-Paredes *et al*. [74]. Animals were distributed in groups according to number of lactation (NL) and stage of lactation (SL) criteria. Furthermore, quartile-based qualitative categories were created for CH_4_ recordings (ppm), resulting in a methane factor (CH4) with 4 levels (LOW, L-MID, H-MID, and HIGH methane emissions).

### Ruminal content sampling

Ruminal fluid was sampled using an oral tube (18 mm diameter and 160 mm long) connected to a 1,000-mL Erlenmeyer flask and continued to a mechanical pump (Vacubrand ME 2SI, Wertheim, Germany), with all the material contacting the cow being carefully cleaned between cows. Each animal was moved to an individual stall for this process. The solid fraction of the ruminal content was discarded by filtering through 4 layers of sterile cheesecloth, while the outcoming liquid fraction was instantly frozen using liquid nitrogen and then stored at −80°C until DNA extraction.

### DNA extraction and sequencing

Genomic DNA was extracted from 250 µL of each thawed and homogenized ruminal content sample, using the “DNeasy Power Soil” commercial kit (Qiagen, Valencia, CA, USA). Qubit fluorometer (ThermoFisher Scientific, Waltham, MA, USA) and Nanodrop ND-1000 UV/Vis spectrophotometer (Nanodrop Technologies Inc., Wilmington, DE, USA) were used to measure DNA concentration and purity. The 260/280 and 260/230 ratios were ∼1.8 and ∼2.0, respectively. Oxford Nanopore Technologies (ONT) SQK-LSK109 Ligation Sequencing kit was used for multiplexed sequencing in MinION automatic sequencer. The 1D Native barcoding ONT kit (EXP-NBD104 or EXP-NBD114) was used for multiplexing the samples, pooling barcoded DNA from 12 samples for each run. Pooling was done using a 1.5-mL DNA LoBind tube to perform adapter ligation and sequenced using a R9.4.1 flow cell.

### Read processing, mapping, and filtering

Guppy toolkit (ONT) was used for basecalling. A quality control was then applied removing sequences with QS <7 and length <150 bp. Sequence analysis was performed using SqueezeMeta (SQM) pipeline for long reads [71], which performs Diamond Blastx against GenBank nr taxonomic database and against COG and KEGG functional databases, then identifying and annotating open reading frames using the LCA method for taxonomy and the fun3 algorithm for functional annotation (based on e-value and identity scores). This tool is specifically designed to process long reads from ONT.

A total of 49,718,901 reads were processed in Blastx by SQM longreads pipeline. Blastx mapped 25,750,755 reads (51.79%) to taxonomy (NCBI-nr database) or function (KEGG database). All sequences mapped as non-microbial (i.e., virus, animals, and plants) were discarded. Microbial sequences were then filtered by prevalence to reduce data sparsity and sequencing errors (Supplementary Data S2). A first estimation of sample sparsity and reads distribution was assessed using R. Two animals were then withdrawn from the filtered dataset, one owing to low read coverage and another owing to lack of host information, leaving 437 animals in the final dataset.

Genera were divided into superkingdom groups (Archaea, Bacteria, or Eukaryota), and KEGGs were sorted by their involvement in methane metabolism (MP): KEGGs included in the KEGG orthology pathway ko00680 (Methane metabolism) were labeled as “ko00680,” while the rest were identified as “Other.”

### Compositional data

Considering the compositional nature of metagenomic data, a CLR method [75] was applied using the unweighted option of the CLR function from the easyCODA R package [76] as follows:

\begin{equation*} {\mathrm{\,\,}}{{\bf{x}}_{{\bf{clr}}}} = \,\,\left[ {\mathrm{ log}({x_1}/G\left( x \right)),\mathrm{ log}({x_2}/G\left( x \right)) \ldots \,\,\mathrm{ log}({x_D}/G\left( x \right))} \right], \end{equation*}

with $G\,\,( x ) = \,\,\sqrt[D]{{{x_1}*{x_2}* \ldots *{x_D}}}$.

Being **x** = [${x_1}$, ${x_2}$,…,$\,\,{x_D}$] a vector of counted features (taxa or KEGGs) in 1 sample and *G(x)* the geometric mean of **x**. Count zero values in the initial data frame were imputed through the Geometric Bayesian Multiplicative procedure, using the zCompositions R package [77] cmultRepl function, so that logarithms could be computed.

### β-diversity and PERMANOVA analysis

The CLR-transformed data (at phylum, class, order, family, genus, and KEGG levels) were used to explore β-diversity in the samples through PCA using the prcomp function in R. Fitted smooth surface of methane emissions corrected by SL and NL was included for principal components 1 and 2 using the ordisurf function from the vegan R package [78]. A generalized additive model smooth fitting (GAM) was used to elucidate non-linear distribution of samples in PCA according to methane emissions. Differences between centroid distances using methane as grouping variable (CH4) were determined through PERMANOVA [79,80] following this model and using the matrix of Aitchison distances between samples (i.e., the Euclidean distance on CLR-transformed data) as input variable:

\begin{equation*} {\mathrm{\,\,}}{D_{jklni}} = \,\,\mu + B_j + \mathrm{ SL}_k + \mathrm{ NL}_l + \mathrm{ CH}4_n + {e_{jklni}}, \end{equation*}

with ${B_j}$ being the farm-batch effect (*j* = 24 levels), $\mathrm{ SL}_k$ being the stage of lactation at the day of sampling (*k* = 3 levels), $\mathrm{ NL}_l$ the number of lactation (*l* = 2 levels), and $\mathrm{ CH}4_n$ the methane emission level (*n* = 4 levels: LOW, L-MID, H-MID, HIGH), and ${e_{jklni}}$ was the corresponding residual term.

### Association between microbiota and methane production

Differential abundance of genera and KEGGs between samples regarding the different methane emissions levels was addressed through linear regression using Limma [81]. Count normalization and log-transformation were addressed using CLR-transformed data as inputs. *P*-values were adjusted by the Benjamini-Hochberg method, to control the false discovery rate (FDR). Differential abundance threshold was set to | log_2_FC | ≥ 0.5 and the adjusted significance threshold was set to α = 0.05.

### Pairwise proportionality analysis

Pairwise correlations between phyla, genera, and KEGGs were calculated as described in the propr R package [82]. Proportionality coefficient *ρ*_p_ [83] under CLR data transformation was chosen. Thresholds were selected according to 2 conditions: (i) representing the maximum number of proportionalities avoiding computational issues; (ii) FDR <1%. The thresholds used were |*ρ*_p_| ≥ 0.4 for genera proportionalities and |*ρ*_p_| ≥ 0.7 for KEGG proportionalities.

### Microbial networks

Microbial networks for taxonomy (at the genus level) and functionality were built from the proportionality matrices described above. Input edges were defined from the cytoscape function in the propr package in R, which converts a propr object into a data frame of node connections compatible with Cytoscape software (v. 3.8.0). Results from the DA analyses were used to associate each feature (node) to high or low methane emissions levels. Significantly overabundant genera and KEGGs in the low methane emitters group (i.e., more abundant in LOW than in HIGH or H-MID groups) were designated as LOW-associated, while those on the contrary overabundant in high methane emitters were appointed as HIGH-associated. Non-DA features were classified as N/A (not associated). In addition, SK and MP factors were included as node attributes for genera and KEGGs, respectively. For graph visualization, Kamada-Kawai algorithm (edge-weighted spring embedded layout) was set [84], using *ρ*_p_ coefficient as force parameter.

## Data Availability

The raw data underlying this article are available at the ENA website and can be accessed with bioproject accession No. PRJNA789746 [85] . The filtered sequences in fastq format and their metadata are available from the *GigaScience* database [86]. Other data can be requested from the METALGEN project [87].

SqueezeMeta software is available at [88]. Guppy basecaller software was used to convert fast5 raw signals to fastq files [89]. The R environment and packages used are available from [90]. Correspondence and material requests should be addressed to O.G.R. Other data further supporting this work (including methane measurements) are openly available in the *GigaScience* repository, GigaDB [86].

## Supplementary Material

giab088_GIGA-D-21-00239_Original_Submission

giab088_GIGA-D-21-00239_Revision_1

giab088_GIGA-D-21-00239_Revision_2

giab088_Response_to_Reviewer_Comments_Original_Submission

giab088_Reviewer_1_Report_Original_Submission danielagaiagaio Gaio -- 9/5/2021 Reviewed

giab088_Reviewer_2_Report_Original_Submission Mick Watson -- 9/8/2021 Reviewed

giab088_Reviewer_2_Report_Revision_1 Mick Watson -- 10/21/2021 Reviewed

giab088_Reviewer_3_Report_Original_Submission Wei Fan -- 9/13/2021 Reviewed

giab088_Reviewer_3_Report_Revision_1 Wei Fan -- 10/18/2021 Reviewed

giab088_Supplemental_Files

## Additional Files

**Supplementary Figure S1. Feature counts distribution**.Features with zero counts, singletons, doubletons and 3 or more counts per sample. A) Count distribution in raw taxonomy table (87% sparsity); B) Count distribution in filtered taxonomy table (68% sparsity); C) Count distribution in raw KEGG table (72% sparsity); D) Count distribution in filtered KEGG table (39% sparsity). Filtering processes removed less than 1% of total reads in both datasets.

**Supplementary Figure S2. Phyla relative abundance per sample**. Samples are sorted from lowest to highest RA of Bacteroidetes.

**Supplementary Data S1. Differential Abundance of genera and KEGGs**. Tables containing significant differentially abundant (DA) genera and KEGGs for methane emissions levels. Contrast.AvsB column represents the CH4 levels for which the feature is significantly DA. Grp.A and Grp.B columns represent the average number of reads per contrast group. For KEGGs, Association column shows if the specific KEGG participates in methanogenesis pathway (k00680) (Methane) or not (Others).

**Supplementary Data S2. Additional methods**.Detailed information about the taxonomy and prevalence filters applied to mapped reads.

## Abbreviations

AMS: automatic milking station; BLAST: Basic Local Alignment Search Tool; bp: base pairs; CLR: centered log-ratio; DA: differential abundance; FC: fold change; FDR: false discovery rate; GAM: generalized additive model; KEGG: Kyoto Encyclopedia of Genes and Genomes; LCA: last common ancestor; NADH: nicotinamide adenine dinucleotide; NCBI: National Center for Biotechnology Information; OA: overabundance; ONT: Oxford Nanopore Technologies; PCA: principal component analysis; PERMANOVA: permutational analysis of variance; RA: relative abundance.
