# Supplementary material for: Fungal and ciliate protozoa are the main rumen microbes associated with methane emissions in dairy cattle
Source: Gigascience. 2022 Jan 25;11:giab088. doi: 10.1093/gigascience/giab088 (PMC8848325; doi:10.1093/gigascience/giab088)
Supplement: giab088_GIGA-D-21-00239_Original_Submission [file giab088_giga-d-21-00239_original_submission.pdf]

GigaScience

Fungal and ciliate protozoa are the main rumen microbes associated with methane emissions in dairy cattle

--Manuscript Draft--

**Manuscript Number:**	GIGA-D-21-00239
**Full Title:**	Fungal and ciliate protozoa are the main rumen microbes associated with methane emissions in dairy cattle
**Article Type:**	Research
**Funding Information:**	Ministerio de Economía y Competitividad (RTA2015-00022-C03-02) — Not applicable<br>INIA: Instituto Nacional de Investigacion y Tecnologia Agraria y Alimentaria (reference FPI-SGIT2016-06) — Mr Adrián López-García
**Abstract:**	Background: This study analyzed whole rumen metagenome using long reads and considering its compositional nature in order to disentangle the role of rumen microbes in methane emissions. Efficient and low-cost strategies must be developed to characterize the taxonomical and functional composition of the rumen microbiome.<br>Methods: Rumen samples from 437 Holstein cows were sequenced using nanopore technology. After filtering, data were treated as compositional using a centered log-ratio transformation before statistical analyses. The association between overall microbiota composition and methane emissions was evaluated with PERMANOVA analysis. Differential abundance analyses were implemented to detect microbial taxa and functions associated to methane production. These associations were depicted in microbial networks.<br>Results: The beta-diversity analyses suggested an association between methane production and overall microbiota composition ($0.01 < R^2 < 0.02$). Differential abundance analysis identified 36 genera and 279 KEGGs as significantly associated to methane production (P_{adj} <0.05). Those genera associated to high methane production were Eukaryota from Alveolata and Fungi clades, while Bacteria were associated to low methane emissions. The genus-level association network showed two clusters grouping Eukaryota and Bacteria, respectively. Regarding microbial gene functions, 41 KEGGs resulted to be differentially abundant and were mainly involved in metabolic pathways. No KEGGs included in the methane metabolism pathway (ko00680) were detected as associated to high methane emissions. The KEGG network showed three clusters grouping KEGGs associated to high emissions, low emissions and not differentially abundant in either of them. A deeper analysis of the differentially abundant KEGGs revealed that genes related with anaerobic respiration through nitrate degradation were more abundant in low emissions animals.<br>Conclusions: This experiment has generated the largest ONT ruminal metagenomic dataset currently available. Methane emissions are largely associated to the relative abundance of ciliate and fungi. The role of nitrate electron acceptors can be particularly important as this respiration mechanism directly competes with methanogenesis. Therefore, whole metagenome sequencing is necessary to jointly consider relative abundance of Bacteria, Archaea and Eukaryota in the statistical analyses. Nutritional and genetic strategies to reduce CH_4 emissions should focus on reducing the relative abundance of Alveolata and Fungi in the rumen.
**Corresponding Author:**	Oscar Gonzalez-Recio<br>INIA: Instituto Nacional de Investigacion y Tecnologia Agraria y Alimentaria<br>Madrid, Ma SPAIN
**Corresponding Author Secondary Information:**	
**Corresponding Author's Institution:**	INIA: Instituto Nacional de Investigacion y Tecnologia Agraria y Alimentaria
**Corresponding Author's Secondary Institution:**	
**First Author:**	Adrián López-García
**First Author Secondary Information:**	

Powered by Editorial Manager® and ProduXion Manager® from Aries Systems Corporation

**Order of Authors:**	Adrián López-García
	Alejandro Saborío-Montero
	Mónica Gutiérrez-Rivas
	Raquel Atxaerandio
	Idoia Goiri
	Aser García-Rodríguez
	José A. Jiménez-Montero
	Carmen González
	Javier Tamames
	Fernando Puente-Sánchez
	Magdalena Serrano
	Rafael Carrasco
	Cristina Óvilo
	Oscar Gonzalez-Recio
**Order of Authors Secondary Information:**	

**Additional Information:**	
**Question**	**Response**
Are you submitting this manuscript to a special series or article collection?	No
**Experimental design and statistics**<br><br>Full details of the experimental design and statistical methods used should be given in the Methods section, as detailed in our Minimum Standards Reporting Checklist. Information essential to interpreting the data presented should be made available in the figure legends.<br><br>Have you included all the information requested in your manuscript?	Yes
**Resources**<br><br>A description of all resources used, including antibodies, cell lines, animals and software tools, with enough information to allow them to be uniquely identified, should be included in the Methods section. Authors are strongly encouraged to cite Research Resource Identifiers (RRIDs) for antibodies, model organisms and tools, where possible.	Yes

Powered by Editorial Manager® and ProduXion Manager® from Aries Systems Corporation

Have you included the information requested as detailed in our Minimum Standards Reporting Checklist?	
**Availability of data and materials**<br><br>All datasets and code on which the conclusions of the paper rely must be either included in your submission or deposited in publicly available repositories (where available and ethically appropriate), referencing such data using a unique identifier in the references and in the “Availability of Data and Materials” section of your manuscript.<br><br>Have you have met the above requirement as detailed in our Minimum Standards Reporting Checklist?	Yes

Powered by Editorial Manager® and ProduXion Manager® from Aries Systems Corporation

Manuscript Click here to access/download;Manuscript;Manuscript.docx

Fungal and ciliate protozoa are the main rumen microbes associated with methane emissions in dairy cattle.

Adrián López-García[1], Alejandro Saborío-Montero[1,2], Mónica Gutiérrez-Rivas[1], Raquel Atxaerandio[3], Idoia Goiri[3], Aser García-Rodríguez[3], Jose A. Jiménez-Montero[4], Carmen González[1], Javier Tamames[5], Fernando Puente-Sánchez[5], Magdalena Serrano[1], Rafael Carrasco[6], Cristina Óvilo[1], Oscar González-Recio[1,7*]

[1]Departamento de Mejora Genética Animal. Instituto Nacional de Investigación y Tecnología Agraria y Alimentaria, Crta. de la Coruña km 7.5, 28040 Madrid, Spain.

[2]Escuela de Zootecnia y Centro de Investigación en Nutrición Animal, Universidad de Costa Rica, 11501, San José, Costa Rica

[3]NEIKER – Instituto Vasco de Investigación y Desarrollo Agrario. Basque Research and Technology Alliance (BRTA). Campus Agroalimentario de Arkaute s/n, 01192 Arkaute, Spain.

[4]Confederación de Asociaciones de Frisona Española (CONAFE). Ctra. de Andalucía km 23600 Valdemoro, 28340 Madrid, Spain.

[5]Departamento de Biología de Sistemas, Centro Nacional de Biotecnología, CSIC, Madrid, Spain

[6]Departamento de Periodismo y Nuevos Medios. Universidad Complutense de Madrid, Ciudad Universitaria s/n, 28040 Madrid, Spain.

[7]Departamento de Producción Agraria. Escuela Técnica Superior de Ingeniería Agronómica, Alimentaria y de Biosistemas. Universidad Politécnica de Madrid, Ciudad Universitaria s/n, 28040 Madrid, Spain.

*** Correspondence:**
Óscar González-Recio
gonzalez.oscar@inia.es

Keywords: dairy cattle, microbiome, rumen, methane, Nanopore, long reads.

1 Abstract

Background: This study analyzed whole rumen metagenome using long reads and considering its compositional nature in order to disentangle the role of rumen microbes in methane emissions. Efficient and low-cost strategies must be developed to characterize the taxonomical and functional composition of the rumen microbiome.

Methods: Rumen samples from 437 Holstein cows were sequenced using nanopore technology. After filtering, data were treated as compositional using a centered log-ratio transformation before statistical analyses. The association between overall microbiota composition and methane emissions was evaluated with PERMANOVA analysis. Differential abundance analyses were implemented to detect microbial taxa and functions associated to methane production. These associations were depicted in microbial networks.

Results: The beta-diversity analyses suggested an association between methane production and overall microbiota composition ($0.01 < R^2 < 0.02$). Differential abundance analysis identified 36 genera and 279 KEGGs as significantly associated to methane production (P_{adj}<0.05). Those genera associated to high methane production were Eukaryota from Alveolata and Fungi clades, while Bacteria were associated to low methane emissions. The genus-level association network showed two clusters grouping Eukaryota and Bacteria, respectively. Regarding microbial gene functions, 41 KEGGs resulted to be differentially abundant and were mainly involved in metabolic pathways. No KEGGs included in the methane metabolism pathway (ko00680) were detected as associated to high methane emissions. The KEGG network showed three clusters grouping KEGGs associated to high emissions, low emissions and not differentially abundant in either of them. A deeper analysis of the differentially abundant KEGGs

revealed that genes related with anaerobic respiration through nitrate degradation were more abundant in low emissions animals.

Conclusions: This experiment has generated the largest ONT ruminal metagenomic dataset currently available. Methane emissions are largely associated to the relative abundance of ciliate and fungi. The role of nitrate electron acceptors can be particularly important as this respiration mechanism directly competes with methanogenesis. Therefore, whole metagenome sequencing is necessary to jointly consider relative abundance of Bacteria, Archaea and Eukaryota in the statistical analyses. Nutritional and genetic strategies to reduce CH_4 emissions should focus on reducing the relative abundance of Alveolata and Fungi in the rumen.

2 Introduction

Next generation sequencing technologies have provided special relevance to microbial communities from different niches, as they allow identifying their taxonomic and functional profile. It has made possible to unravel the relationships between host and microbiota, as well as the complex interactions between microbes, with a special contribution to the role of digestive microbiome on complex traits both in humans [1] (e.g. type II diabetes, cancer, mental diseases) and in domestic animals [2,3] (e.g. feed efficiency, methane emissions, animal health).

Microbial communities are of special relevance in livestock. In ruminants, one of the main microbial communities lays in the rumen, due to its high diversity and large microbial mass [4] and its main role in feed fermentation to provide substrate to the animal, which is then transformed into product. Additionally, enteric methane is produced in the rumen by methanogenic microorganisms during feed fermentation [5] and is the main contributor of greenhouse gases (GHG) from livestock, with around 2,448 million tonnes

of CO_2-equivalent (CO_2e) per year [6,7]. The ongoing climate emergency urgently calls for efficient strategies to mitigate the carbon footprint from all sectors, including agriculture and livestock farming. Former studies have proven that complex traits in ruminants are usually influenced by global changes in microbial communities, more than by fluctuations in the abundance of specific microorganisms [8,9]. These global changes are usually due to the intricate interactions between different species in these communities (*i.e.*, predation, competition of ecological niche or co-dependency). Consequently, a better understanding of the interactions between microbial genes during methanogenesis is needed to propose strategies for reducing methane emissions. Promising strategies have been proposed to modulate the metagenome, nutrition and genetics [10].

Classical statistical approaches do not allow to accurately assess the results of microbiome studies. The high sparsity of these data and their compositional nature generate multiple problems in statistical analysis, including subcompositional incoherence, increase of false positive rates in differential abundance analyses and detection of spurious correlations [11].

As a consequence, new approaches considering both compositionality and multiple correlations are needed. It is also important to point out the advantages of whole metagenome sequencing over metataxonomic studies, because the latter cannot be used to determine functionality and because they pose some difficulties at simultaneously analyzing different superkingdoms [12], which is necessary to account for the total variability of microbiomes and the interactions among their components. Different amplicons must be used to correctly classify Bacteria, Archaea, Protozoa and Fungi, increasing the cost of the studies and involving additional bias due to PCR [13]. They pose the additional difficulty of a proper comparison between communities sequenced in different reactions with different primers. Nanopore sequencing offers a cost-efficient

sequencing strategy for metagenomics studies providing both taxonomical and functional information simultaneously and for microbes from all superkingdoms. This technology has been improved in recent years, allowing to perform taxonomic and functional assignments with an accuracy comparable to Illumina [14].

The objective of this study was to characterize the taxonomical and functional composition of rumen microbiota using long sequence reads obtained with Nanopore technology, and their relationship with enteric methane emission.

3 Results

3.1 Cohort description

Our cohort included 437 Holstein lactating cows sampled at 14 different herds from northern Spain (Cantabria, Euskadi, Navarra and Girona regions).

3.2 Taxonomy of microbial composition

After initial quality control, 6,394,671 reads with N50=4194 bp were classified in 3,921 taxonomical features up to genus level. A filtering strategy was implemented to filter out low abundance microbes while keeping the core microbiome relevant for methane emissions. This filtering excluded 48,517 reads (<1%) which reduced the sparsity of the metagenome from 87% to 68%, although a large number of singleton and doubleton features remained (**Figure 1A**). The final core subcomposition included a total of 6,318,344 reads, in 437 samples, classified in 1,240 taxonomical features: 967 known genera (722 bacteria, 13 archaea and 232 eukaryotes), and 273 that only reached family rank (*i.e.*, *Unclassified* denomination). Overall, 503 families, 277 orders, 158 classes and 86 different phyla (37 bacterial phyla, 3 archaeal phyla and 46 eukaryotic clades) were classified.

Predominant microorganisms in this core rumen subcomposition were bacteria (91.6% ± 6.93 of total average RA) from Bacteroidetes, Firmicutes and Fibrobacteres (**Figure 2**), representing an average relative abundance (RA) of 63%, 16% and 5%, respectively. The Bacteroidetes fraction was majorly composed by *Prevotella*, and was the main representative genus in the total community (19.4% average RA), along with other Prevotellaceae members. The Firmicutes group included a large number of genera. The order of Clostridiales dominated in terms of RA, with Lachnospiraceae and Ruminococcaceae families being the most representative ones. The remaining phyla (34) from the Bacteria superkingdom represented 7.6% averaged RA of the core metagenome. Eukaryotes represented a total average RA of 8.2% (±6.95) of the core subcomposition. Predominant eukaryotic clades were those included in the SAR supergroup (Stramenopiles-Alveolata-Rhizaria) [15], accounting for 6% of total average RA, followed by Fungi (1.3% of total average RA). Alveolata clade was the most abundant among the eukaryotes, with a high representation of unclassified Ophryoscolecidae, *Stentor* and *Paramecium*. Archaea representation in the core subcomposition (0.24% ± 0.25 of total average RA) consisted mostly of Methanomicrobia, Methanobacteria and Thermoplasmata members. Yet, a large number of reads could not be assigned to a known genus. The relative abundance per animal of the most relevant taxonomic groups is depicted in **Figure 3**.

3.3 Functionality of microbial composition

A total of 30,326,550 reads were assigned to KEGGs. After quality control and prevalence filtering, a total of 84,219 reads (0,28%) were removed and the sparsity was reduced from 72% to 39% (**Figure 1B**). The final KEGG table was composed by 30,145,459 reads from 437 samples, classified in 6,644 KEGGs. These KEGG pathways and BRITE hierarchies [16–18] were represented in a Treemap according to their average

RA (**Figure 4**). Most of the rumen metagenome functions were in pathways that represent the metabolism of carbohydrate, amino acid and other biological compounds, as well as of energy metabolism. In addition, functions involved in cellular generic processes (cell growth, transport, or genetic and environmental information processing) were also present. KEGG BRITE classification showed a high presence of proteins involved in cellular processes and metabolism.

3.4 Beta-diversity and PERMANOVA analysis

Beta-diversity was represented in PCA between samples at five different taxonomic levels (phylum, class, order, family and genus), as well as with KEGG, using centered log-ratio (CLR) transformed datasets. Then a permutational analysis of variance (PERMANOVA) was implemented (Gloor et al., 2017), sequentially adding the effect of farm-batch (B), stage of lactation (SL), number of lactation (NL) and level of methane emissions (CH4) discretized in four groups (LOW, L-MID, H-MID and HIGH). The visualization did not show a clear visual clustering of samples by methane emission levels (**Figure 5**). However, a generalized additive model (GAM) smooth fitting allowed visualizing non-linear distribution patterns of the microbial samples according to CH_4 emissions inside the ordination at all taxonomic levels. The non-linear pattern was more evident at the phylum, class and genus levels, although the proportion of methane variability explained was low (≃4.8% according to GAM model fitting). No relevant differences were visually appreciated using the KEGG information. Nonetheless, some differences in the overall rumen microbiome composition between animals with different methane emissions were evidenced by the PERMANOVA analysis, both for taxonomy and functionality (Table 1). The results showed significant differences for the centroid distance between methane emission groups at every taxonomic level and also for KEGGs, but they explained a low percentage of total variance ($0.01 < R^2 < 0.02$).

3.5 Rumen microbes associated to CH_4 emissions

The effect of taxonomical features on methane emission levels was evaluated through differential abundance analysis. Thirty-three genera were found as differentially abundant (DA) (P_{adj} <0.05) between LOW and HIGH emitters (**Figure 6A**), while 15 genera showed DA between LOW and H-MID emitters and one genus between LOW and L-MID emitters (**Supplementary Data 1**). Note that 13 out of the 15 genera showing DA (P_{adj} <0.05) between LOW and H-MID groups were also significant in the LOW vs HIGH contrast, but not in LOW vs L-MID contrast, indicating gradual abundance change from low to high emitters. Accounting for all contrasts and duplicated genera, 36 DA genera resulted significant. We classified these genera according to in which group they resulted overabundant (OA). Thus, 10 of them were more abundant in the LOW group (LOW-OA) and 1 in the L-MID group. The remaining 25 genera were OA in the HIGH groups (HIGH-OA): HIGH (12), HIGH and H-MID (11) or H-MID (2). HIGH-OA genera represented an overall RA of 4.15%, whereas LOW-OA genera accounted for 0.25% of total RA. The two genera over-abundant in H-MID were *Dictyostelium* and *Unclassified Eimeriidae*, and the one associated to L-MID was classified as *Candidatus Izimaplasma* (Tenericutes). The log_2FC values ranged between 0.7 and -0.7 in genera showing DA for methane emission levels, highlighting that the differences between groups were moderate.

Overall, DA results indicate that taxa associated to higher methane levels belong to the Eukaryota superkingdom, while those associated to lower emissions were bacteria. We found multiple Ciliophora genera associated to the HIGH group (mostly Parameciidae, Stentoridae and Pseudocohnilembidae members) but also Amoebozoa and some Fungi or Pseudo-fungi. Other bacterial genera associated to lower methane production were *Hespellia*, from Clostridiales, and *Sutterella*, an asaccharolytic genus from Betaproteobacteria.

3.6 Microbial gene function associated to CH_4 emissions

Differential abundance analysis was also performed for KEGG features on methane emission levels. A total of 192 KEGGs were DA between the LOW and HIGH emissions groups (**Figure 6B**). Differences were also found between the LOW and H-MID groups (**Supplementary Data 1**). As in the taxonomy dataset, some of the KEGGs presented significant DA in both LOW vs HIGH and LOW vs H-MID contrasts. Accounting for these duplicates and all the contrasts, 182 were over-abundant in the high emissions groups (HIGH-OA), whereas 97 KEGGs were over-abundant in low emissions groups (LOW-OA). The overall RA for HIGH-OA KEGGs was 2.31% and 0.64% for LOW-OA KEGGs. Of these, 13 HIGH-OA KEGGs and 28 LOW-OA KEGGs were assigned to metabolic pathways. No KEGGs from the ko00680 pathway were found as HIGH-OA. KEGGs related to inositol-phosphate metabolism (K00889, K01110, K18082 and K20279), starch and sucrose metabolism (K01203) or several lipid metabolism pathways were present in the HIGH-OA group. According to LOW-OA KEGGs, some of them were involved in volatile fatty acid (VFA) metabolism (e.g., K00209 enoyl-[acyl-carrier protein] reductase [EC:1.3.1.9], K01902 succinyl-CoA synthetase alpha subunit [EC:6.2.1.5] and K01682 aconitate hydratase 2 [EC:4.2.1.3]) and the K09251 putrescine aminotransferase [EC:2.6.1.82] related to putrescine and cadaverine degradation to 4-amino-butanoate (GABA) or 2-oxoglutarate. Also, several KEGGs in the LOW-OA group were related to N metabolism (K00370 and K00371 nitrate reductase subunits [EC:1.7.5.1]), oxidative phosphorylation (K03885 NADH dehydrogenase [EC:1.6.99.3]) and to carbohydrate, lipid or vitamin metabolism pathways. The ko00680 KEGG K13788 was also over-abundant in the LOW emissions group.

3.7 Interaction networks

Interaction networks were built using the previous results in order to visualize the association between taxa and genes using pairwise correlations between features. Pairwise proportionality correlation coefficients (ρ_p) were calculated on the CLR-transformed datasets for phylum, genus and KEGG features to avoid spurious correlations that can potentially surge in compositional data [19].

The most relevant pairwise proportionalities between genera and between KEGGs were visualized as interaction networks, classifying features as associated to high methane emissions (HIGH), low methane emissions (LOW) or not associated to methane emissions (N/A), according to the results from the differential abundance analyses. The interaction networks for genera and KEGGs are shown in **Figure 7** and **Figure 8**, respectively.

Eukaryotes clustered together in the network with large representation of the SAR supergroup, and showed negative proportionality to Bacteria. The genera that were associated to higher methane emissions belonged to the Eukaryota superkingdom (Ciliophora and Fungi), whereas Bacteria were associated to lower CH_4 production. The strongest inverse proportionalities between both subpopulations connected several eukaryotes with *Unclassified Veillonellaceae* and *Oribacterium* ($-0.64 < \rho_p < -0.53$), *i.e.*, microbiomes with lower abundance of *Oribacterium* or Veillonellaceae tend to present larger abundances of protozoa and Fungi, and were therefore associated to larger emissions. Unclassified microbes from Neocallimastigaceae, Oxytrichidae and Vibrionaceae families showed the highest centrality and a large connectivity degree.

The functional network showed three main clusters that grouped KEGGs associated to HIGH methane level (cluster H), KEGGs not related to methane emissions (cluster N), and a small one including KEGGs associated to lower emissions (cluster L). Connections

between clusters were not symmetric: H cluster was connected to N cluster by inverse proportionalities between some of their components, but the L cluster appeared connected only to N cluster by direct proportionalities through non-clustered KEGGs. Also, most of the ko00680 KEGGs (*i.e.*, directly involved in methanogenesis or participating in pathways leading to methanogenesis precursors) did not appear as differentially abundant between high-emission and low-emission cows.

3.8 Taxonomy of genes

A traceback of genes' taxonomy was carried out, separately for ko00680 KEGGs and for DA KEGGs. Thirty out of the 85 ko00680 KEGGs were predominant in Archaea groups, one predominated in Eukaryota (K05979) and the rest were predominant in Bacteria (**Figure 9**). Although the RA distribution of these KEGGs was normally between 60% and 100% in the predominant superkingdom, 4 KEGGs were more evenly distributed between clades: K01007 and K00863 had a RA < 60% in Bacteria and showed RA > 30% in Eukaryota; K05979 was the KEGG predominating in Eukaryota, but with a RA near to 60% (38% in Bacteria and 12% in Archaea); and K14080 had a RA of 57% in Archaea and 43% in Bacteria. Regarding the DA KEGGs, those from the LOW-OA group showed larger abundance in Bacteria, mostly in genera from Proteobacteria, Bacteroidetes and Firmicutes phyla. Different groups of bacteria also carried KEGGs from the HIGH-OA group although these KEGGs were more abundant in eukaryotes. The HIGH-OA KEGGs were mainly mapped to unclassified eukaryotes, but those which could be classified belonged majorly to Fungi and SAR supergroup (**Figure 10**).

4 Discussion

In this study we assessed the composition of the ruminal microbiota using long reads from Nanopore sequencing technology. We observed predominance of Bacteroidetes,

Firmicutes and Fibrobacteres in the rumen metagenome, as reported in previous studies [8,20]. Bacteroidetes and Firmicutes are common bacteria in all kind of ecosystems, including gut microbiota of multiple animals. The fraction of Bacteroidetes was mainly composed by *Prevotella*. This group includes anaerobic gram-negative bacteria involved in saccharolytic processes [21]. Their large abundance in the digestive microbiota has been previously reported in ruminant [22–25] and monogastric species [26,27]. A wide representation of polysaccharide fermenters is represented in the rumen communities [28]. Fibrobacteres comprises a small group of cellulose-degrading bacteria usually present in ruminant digestive system [29]. Eukaryotes also represented a relevant amount of the rumen core metagenome. This group has been reported to contribute up to 50% of total ruminal biomass [30]. The SAR supergroup and Fungi were the most relevant ones [15]. This group of Eukaryota is found in a wide variety of ruminants and pseudoruminants [31]. Other eukaryotes included *Stentor*, aquatic free-living heterotricheans which can be particle filtrators or predators of other protozoa, and usually live symbiotically with some algae species [32,33]. Also *Paramecium* are well-known ciliates which predate bacteria and other microorganisms, including protozoa [34]. Archaeal fraction was mostly composed by strict methanogenic organisms from Methanomicrobia and Methanobacteria clades [35], but also included Thermoplasmata, which are methylotrophic-methanogenic acidophilic organisms [36]. Gene Ontology associated found KEGGs to several metabolic functions as well as cellular processes. Additionally, pathways related to pathogenic activity were also present, in agreement with the RA of several genera that included some known pathogenic species. For instance, some species from genera such as *Vibrio*, *Haemophilus*, *Trypanosoma* or *Staphylococcus*, although not every species from these genera are pathogenic, but opportunistic or commensal organisms. In addition, pathogenic activity

presence in our dataset might be biased due to a larger representation of human related diseases in the databases.

The SqueezeMeta software applies a stringent threshold for taxonomy classification to ensure that reads have a large probability of being correctly classified, at expense of a large number of reads remaining unclassified, which explains the larger number of reads assigned to a known KEGG than to taxa. Despite this strict requirement, this composition is consistent with other populations reported before [2,3,20]. Most studies to date report large abundance of *Bacteroidetes* and *Firmicutes*, with *Prevotella spp*. as the most prevalent genus. Some minor discrepancies with other studies were observed in the RA of the core subcomposition. For example, Wallace et al. [20] showed a higher presence of Proteobacteria and Euryarchaeota, although using amplicons instead of whole metagenome sequencing.

We performed several statistical approaches to infer association between the rumen metagenome and methane emissions. Our approach evidenced the difficulty of inferring a phenotypic association between microbiome composition and methane production, with environmental factors covering the statistical signal. However, our compositional approach showed a meaningful relationship between the microbiome composition and methane emissions, emphasizing the role of the different phyla, with Eukaryota superkingdom being of particular relevance. Microbial networks contributed to detect genes associated to ko00680 pathway and elucidated some methane emission dynamics. These interactions configure the level of methane production and must be considered jointly at modulating the rumen microbiome. Former studies already revealed the link between ruminal microbiota and methane production. For instances, Difford et al. [3] observed clustering of high and low methane emitters within bacterial and archaeal subcommunities. Danielsson et al. [37] also found clustering for low and high methane

emitters within bacterial rumen subcompositions. Wallace et al. [20] found that a core set of rumen microbiome was capable of explaining up to 30% of methane emissions variability, this set mostly formed by prokaryotes. The aforementioned studies used different methodologies, like amplicon analysis and OTU clustering, contrasting with our full-metagenome genus-clustering protocol, which increases the information entropy. Stewart et al. [38] used Nanopore sequencing and found significant differences between low and high-methane emitter sheep, with clear clustering between groups but using a lower number of microbial groups, and animals in the same farm with similar management practices.

The DA analysis allowed us to define specific taxa that were different between high and low methane emitters. Ciliates, fungi and pseudo-fungi were more abundant in cows with higher levels of methane emissions. Microbes associated to lower methane emissions were saccharolytic members of class Gammaproteobacteria (*Anaerobiospirillum* [39], *Vibrio* [40] or *Pseudoalteromonas* [41]), as well as Negativicutes genera from Veillonellaceae (*Dialister*, *Megasphaera*) and Selenomonadaceae (*Mitsuokella*). *Dialister* produce succinate decarboxylation, and *Megasphaera* ferment carbohydrate and lactate [42], while *Mitsuokella* are saccharolytic bacteria [43]. Interestingly, no taxonomic group of methanogenic archaea showed association with methane emissions. The relationship between Archaea and methane production in rumen is not consistent in the literature. Some authors reported either individual relationships between methane emissions and some archaeal species [37,44] or correlations between overall archaeal gene abundance and methane emissions level [45,46]. However, other studies showed no relationship between methanogenic *Archaea* and methane [44,47]. Ciliates play a central role in the abundance of archaea, as many are known to symbiotically engulf a variety of methanogenic archaea [48]. The association between protozoa abundance in rumen and methane emissions is well

known, as protozoa have an impact in the archaeal population structure [49] and some protozoa defaunation experiments, both *in vitro* [50,51] and *in vivo* [52,53], have demonstrated a reduction in methane emissions [54]. This may explain the relevant association estimated between ciliates and fungi with methane.

Despite this association between methane and large taxonomic groups, it is of interest to infer which specific clades and microbial genes are participating directly or indirectly in methanogenesis. This may help at disentangling the role of the different microbes in feed fermentation and methanogenesis. Microbial networks contributed to elucidate methane emission dynamics and associate genes to pathways indirectly related to methane metabolism. They clearly clustered eukaryotes together, with many of them being significantly more abundant in the high emissions group. Other authors have already established a positive correlation between fungi abundance and methane emissions [8], as well as a close interdependence of protists and fungi. Although correlation between methane emissions and protozoa abundances is still a matter of discussion [45,53], current meta analyses point to a linear relationship between protozoan numbers and methane emissions ($r=0.96$) [55]. Among eukaryotes, anaerobic protozoa and some *Chytridiomycota* produce H_2 in their hydrogenosomes [56] (e.g. *Neocallimastix sp.*). These organelles supply endosymbiotic methanogenic archaea with substrate for methane production and provide protection against oxygen toxicity [30,48]. Methanogenic taxa and their enzymes were not highly represented in the high-emissions cluster. The relative abundance of archaea in the rumen is low compared to eukaryotes and bacteria. However, they are tightly linked to ciliate protozoa and fungi. Free-living methanogens represent a low-abundant population [55], and CH_4 biosynthesis might be more influenced by methanogens engulfed by protozoa, mostly ciliates [48]. Hence, a larger methanogenesis activity is expected to be correlated with a larger abundance of eukaryotes which are more abundant and better represented in

the network than archaea. Furthermore, lysis of archaea cell walls often requires specific protocols during DNA extraction, and they might be under-represented [57] in metagenomic studies, including this experiment. This could partially explain the lack of association between archaea abundance and methane in some previous studies [10]. On the other hand, the ruminotype in low-emissions animals has more abundance of *Proteobacteria* and *Firmicutes* genera. Other authors also reported higher abundances of these bacterial phyla in low methane emissions animals [8]. Also, lactate- and succinate-producers have been reported to be more abundant in low-emitters [58], supporting the higher abundance of *Anaerobiospirillum* or *Megasphaera* in LOW animals.

According to microbial genes, we firstly classified KEGGs according to their presence or absence in ko00680 pathway (methane metabolism), as a way to evaluate their direct involvement in methanogenesis or else their participation in pathways leading to biosynthesis of precursor compounds. Although we found several ko00680 KEGGs which are presumably involved in the biosynthesis of methanogenesis precursors, most of them were not associated to methane emissions (*i.e.*, not differentially abundant between methane groups), as it can be visualized in the KEGG network. The taxonomy distribution of these KEGGs showed that most of them are mainly present in bacteria or eukaryotes and might be functioning in metabolic pathways not related with methanogenesis. For instance, some of the KEGGs inside the methane metabolism pathway can also be involved in glycine, serine and threonine metabolism (e.g. K00058, K00831, K01079 and K00600), pyruvate and propanoate metabolism (e.g. K00625 and K13788), glycolysis (e.g. K01689, K15633, K01624 and K02446) or anaerobic carbon fixation (e.g. K00198) [16–18]. Furthermore, the presence of genes does not mean that they are transcribed. In a metagenomic analysis, all present genes are sequenced regardless whether they are transcribed or not. Also, bacterial epigenetic mechanisms affected by

the animal diet could play a role in this gene expression [59]. Another group of ko00680 KEGGs is exclusive from *Archaea*, but the under-representation of this clade in our dataset might obscure statistical significance.

Since no methanogenesis KEGGs were detected as associated to methane emissions, a deeper evaluation of individual DA genes allowed us to find some KEGGs which could be involved in the biosynthesis of methanogenesis precursors, or else influence the abundance of methanogenic archaea.

We found some KEGGs which could be indirectly related with methanogenesis through biosynthesis of precursor compounds. K00209 and K13788 are involved in butyrate and propanoate biosynthesis, being essentially carried by primary fermentative bacteria [60]. Their relationship with methanogenesis is indicated by the fact that VFA can be used by secondary fermenters to produce methanogenesis precursors such as H_2, CO_2, acetate and formate [61,62]. In fact, K13788 is a phosphate acetyltransferase [EC:2.3.1.8] that can be involved in the biosynthesis of acetate from acetyl-CoA [63]. Also, K09251 is involved in biosynthesis of GABA and 2-oxoglutarate. GABA has been related with a VFA concentration increment [64], while 2-oxoacid compounds can be used by archaea has been linked to synthesize coenzyme M and coenzyme B, which are essential in methane production in methanogens [65]. However, all these KEGGs were observed as over-abundant in LOW methane group, suggesting a strong presence of fermentative bacteria in these animals, not directly correlated with methane production.

Other KEGGs that were over-abundant in LOW emitters might offer an explanation to the lower presence of active methanogenesis processes through competence mechanisms. LOW-OA KEGGs K01682, K01902 and, once more, K13788, are involved in citrate cycle and pyruvate metabolism, related to respiration. Also, KEGGs K00370 and K00371

are nitrate oxidoreductase subunits having a role in anaerobic respiration using nitrate as electron acceptor. This enzyme uses nitrate as electron acceptor, a process that has been reported as competitive inhibitor of methanogenesis [66,67] and nitrate supplementation has proven a useful strategy to mitigate methane emissions [68]. Furthermore, nitrite produced by the nitrate-reductases has a known antimicrobial effect and toxicity to animal cells [69–71], which might as well reduce the proportion of free archaea in LOW animals, although toxicity to archaea must be further studied [72]. However, the role of ciliates and fungi must be clarified, as their abundance is also lower in LOW emitters. We hypothesize that the predatory nature of these eukaryotes might be a control mechanism for bacterial populations, and their lower relative abundance in LOW animals might allow overgrowth of related bacteria. Nevertheless, we cannot discard the possibility that a higher proportion of facultative anaerobes using nitrate as acceptor might affect ciliate populations by toxicity, thus reducing the presence of endosymbiotic methanogenic archaea.

5 Conclusions

This study grants a huge amount of metagenomic information, since we generated the largest publicly-available ruminal metagenomic dataset sequenced using ONT long reads approach. The complexity of the rumen microbiome and the compositional nature of their sequencing data require proper statistical methods to allow disentangling the role of microbes and their genes in host complex traits such as methane emissions. The full metagenome compositional analysis used in this study provided novel insights in the association between the microbiota and CH_4 emissions through differential abundance analysis, pairwise correlation and interaction networks.

Our approach evidenced a phenotypic association between microbiome composition and methane production, regardless of the challenges posed by the microbiome complexity and the compositional nature of the data. This association is mainly driven by the relative abundance of ciliates and fungi, which carry host specific genetic functions providing substrate to the methanogenic archaea. On the other side, we detected some bacterial groups that performed a more efficient feed digestion, leaving less hydrogen available to archaea and hence associated to lower methane emissions. Further studies must be carried out to determine proper nutritional and breeding strategies that modulate the microbiome composition towards lower emissions and larger feed efficiency.

6 Methods

6.1 Animal housing and feeding

The animals received total mixed ration (TMR) diet differently formulated on each individual herd, although most of them were based on maize and grass silage plus concentrate. Cows were fed ad-libitum, with concentrate supplementation in the automatic milking station (AMS) during milking.

6.2 Methane measuring

Methane concentration was individually recorded through breath sampling during each cow visit to the AMS (3-7 times daily) in a period of 2-3 weeks. Eructation peaks were recorded using a non-dispersive infrared methane detector (Guardian NG infrared gas monitor, Edinburgh Sensors, Scotland, UK) as described by Rey et al. (2019) [73]. Each cow's peaks were then averaged in order to get a unique methane record per cow, as described in López-Paredes et al. (2020) [74]. Animals were distributed in groups according to number of lactation (NL) and stage of lactation (SL) criteria. Furthermore, quartile-

based qualitative categories were created for CH_4 recordings (ppm), resulting in a methane factor (CH4) with 4 levels (LOW, L-MID, H-MID and HIGH methane emissions).

6.3 Ruminal content sampling

Ruminal fluid was sampled using an oral tube (18 mm diameter and 160 mm long) connected to a 1000 mL Erlenmeyer flask and continued to a mechanical pump (Vacubrand ME 2SI, Wertheim, Germany), with all the material contacting the cow being carefully cleaned between cows. Each animal was moved to an individual stall for this process. The solid fraction of the ruminal content was discarded by filtering through 4 layers of sterile cheesecloth, while the outcoming liquid fraction was instantly frozen using liquid nitrogen (LN_2) and then stored at -80 °C until DNA extraction.

6.4 DNA extraction and sequencing

Genomic DNA was extracted from 250 µl of each thawed and homogenized ruminal content sample, using the "DNeasy Power Soil" commercial kit (QIAGEN, Valencia, CA, USA). Qubit fluorometer (ThermoFisher Scientific, 150 Waltham, MA, USA) and Nanodrop ND-1000 UV/Vis spectrophotometer (Nanodrop Technologies Inc., DE, USA) were used to measure DNA concentration and purity. 260/280 and 260/230 ratios were around 1.8 and 2.0, respectively. Oxford Nanopore Technologies (ONT) SQK-LSK109 Ligation Sequencing kit was used for multiplexed sequencing in MinION automatic sequencer. The 1D Native barcoding ONT kit (EXP-NBD104 or EXP-NBD114) was used for multiplexing the samples, pooling barcoded DNA from 12 samples for each run. Pooling was done using a 1.5 ml DNA LoBind tube to perform adapter ligation and sequenced using a R9.4.1 flow cell.

6.5 Bioinformatics

Guppy toolkit (ONT) was used for basecalling. A quality control was then applied removing sequences with QS<7 and length<150 bp. Sequence analysis was performed using SqueezeMeta (SQM) pipeline for long reads [75], which performs Diamond Blastx against GenBank nr taxonomic database and against COG and KEGG functional databases, then identifying and annotating ORFs using the lca (last common ancestor) method for taxonomy and the fun3 algorithm for functional annotation (based on e-value and identity scores). This tool is specifically designed to process long reads from ONT.

49,718,901 reads were processed in Blastx by SQM longreads pipeline. Blastx mapped 25,750,755 reads (51.79%) to taxonomy (NCBI-nr database) or function (KEGG database). All sequences mapped as non-microbial (*i.e.*, virus, animals and plants) were discarded. Microbial sequences were then filtered by prevalence to reduce data sparsity and sequencing errors (**Supplementary Data 2**). A first estimation of sample sparsity and reads distribution was assessed using R. Two animals were then withdrawn from the filtered dataset, one due to low read coverage and other due to lack of host information, leaving 437 animals in the final dataset.

Genera were divided into superkingdom groups (Archaea, Bacteria or Eukaryota) and KEGGs were sorted by their involvement in methane metabolism (MP): KEGGs included in the KEGG orthology pathway ko00680 (Methane metabolism) were labeled as “ko00680”, while the rest were identified as “Other”.

6.6 Compositional data

Considering the compositional nature of metagenomic data, a CLR method [76] was applied using the unweighted option of the *CLR* function from the *easyCODA* R package [77] as follows:

$$\mathbf{x}_{\mathbf{clr}} = [log(x_1/G(x)), log(x_2/G(x)) \ldots log(x_D/G(x))],$$

with $G(x) = \sqrt[D]{x_1 * x_2 * \ldots * x_D}$.

Being **x** = $[x_1, x_2, \ldots, x_D]$ a vector of counted features (taxa or KEGGs) in one sample and *G(x)* the geometric mean of **x**. Count zero values in the initial data frame were imputed through the Geometric Bayesian Multiplicative (GBM) procedure, using the *zCompositions* R package [78] *cmultRepl* function, so that logarithms could be computed.

6.7 Beta-diversity and PERMANOVA analysis

The CLR-transformed data (at phylum, class, order, family, genus and KEGG levels) were used to explore beta-diversity in the samples through PCA using the *prcomp* function in R. Fitted smooth surface of methane emissions corrected by SL and NL was included for principal components 1 and 2 using *ordisurf* function from the vegan R package [79]. A generalized additive model smooth fitting (GAM) was used in order to elucidate non-linear distribution of samples in PCA according to methane emissions. Differences between centroid distances using methane as grouping variable (*CH4*) were determined through Permutational Multivariate Analysis of Variance (PERMANOVA) [80,81] following this model and using the matrix of Aitchison distances between samples (*i.e.*, the Euclidean distance on CLR-transformed data) as input variable:

$$D_{jklni} = \mu + B_j + SL_k + NL_l + CH4_n + e_{jklni}$$

with B_j being the farm-batch effect (j = 24 levels), SL_k being the stage of lactation at the day of sampling (k = 3 levels), NL_l the number of lactation (l = 2 levels) and $CH4_n$ the methane emission level (n = 4 levels: LOW, L-MID, H-MID, HIGH), and e_{jklni} was the corresponding residual term.

6.8 Association between microbiota and methane production

Differential abundance of genera and KEGGs between samples regarding the different methane emissions levels was addressed through linear regression using Limma [82]. Count normalization and log-transformation were addressed using CLR-transformed data as inputs. P-values were adjusted by Benjamini-Hochberg method, to control false discovery rate. Differential abundance threshold was set to $| \log_2 FC | \geq 0.5$ and the adjusted significance threshold was set to $\alpha = 0.05$.

6.9 Pairwise proportionality analysis

Pairwise correlations between phyla, genera and KEGGs were calculated as described in the *propr* R package [83]. Proportionality coefficient ρ_p [84] under CLR data transformation was chosen. Thresholds were selected according to two conditions: 1) representing the maximum number of proportionalities avoiding computational issues; 2) FDR lower than 1%. Used threshold were $|\rho_p| \geq 0.4$ for genera proportionalities and $|\rho_p| \geq 0.7$ for KEGG proportionalities.

6.10 Microbial networks

Microbial networks for taxonomy (at the genus level) and functionality were built from the proportionality matrices described above. Input edges were defined from the cytoscape function in *propr* package in R, which converts a propr object into a data frame of node connections compatible with Cytoscape software (v. 3.8.0). Results from the DA

analyses were used to associate each feature (node) to high or low methane emissions levels. Significantly over-abundant genera and KEGGs in the low methane emitters group (*i.e.*, more abundant in LOW than in HIGH or H-MID groups) were designated as LOW-associated, while those contrary over-abundant in high methane emitters were appointed as HIGH-associated. Non-DA features were classified as N/A (not associated). In addition, SK and MP factors were included as node attributes for genera and KEGGs, respectively. For graph visualization, Kamada-Kawai algorithm (Edge-weighted spring embedded layout) was set [85], using ρ_p coefficient as force parameter.

7 Ethical statement

This study was conducted in accordance with Spanish Royal Decree 53/2013 for the protection of animals used for experimental and other scientific purposes and was approved by the Basque Institute for Agricultural Research and Development Ethics Committee (Neiker-OEBA-2017-004) on March 28, 2017.

8 Conflict of Interest

The authors have not stated any conflicts of interest.

9 Author Contributions

A.L.G. and A.S.M. filtered and prepared the data, implemented the statistical analyses and prepared the first draft of the manuscript. M.G.R and C.G. performed the DNA extraction and sequencing. O.G.R. supervised the DNA sequencing and contributed to the statistical analyses. R.C. contributed to develop interaction networks. O.G.R., A.G.R, R.A., I.G. conceived the study and designed the experiments. J.T. and F.P.S developed the computational pipelines for the metagenome and assisted on its analyses. A.L.G.,

A.S.M. and O.G.R. wrote the manuscript. All authors helped writing and configuring the last version of the manuscript.

10 Funding

This research was financed by RTA2015-00022-C03-02 (METALGEN) project from the national plan of research, development and innovation 2013-2020 and the Department of Economic Development and Competitiveness (Madrid, Spain).

11 Acknowledgments

We thank the regional Holstein Associations and farmers collaborating in the project. Computational support from the High-Performance Computing Centre in Galicia (Spain) is acknowledged. A.L.G. was funded by FPI-INIA grant with reference FPI-SGIT2016-06.

12 Consent for publication

Not applicable.

13 Data and material

The data set supporting the results of this article are available in the European Nucleotide Archive (ENA) repository, with accession number PRJEB44278.

14 Availability of source code and requirements

Project name: SqueezeMeta

Project home page: https://github.com/jtamames/SqueezeMeta

Operating system(s): x86_64 Linux OS

Programming language: C, C++, Python, Perl, R, Roff

Other requirements: Conda

License: GNU GPL v3

Project name: Guppy

Project home page: https://community.nanoporetech.com/downloads

Operating system(s): Platform independent

License: N/A

Project name: CoreOme

Project home page: https://github.com/alopgar/CoreOme

Operating system(s): Platform independent

Programming language: R, shell, awk

Other requirements: R 3.6.1 or higher

License: GNU GPL v3

Project name: R-metagenomics

Project home page: https://github.com/alopgar/R-metagenomics

Operating system(s): Platform independent

Programming language: R

Other requirements: R 3.6.1 or higher

License: GNU GPL v3

15 References

1. Wang, B., Yao, M., Lv, L., Ling, Z. & Li, L. The Human Microbiota in Health and Disease. *Engineering* **3**, 71–82 (2017).

2. Delgado, B. *et al.* Whole rumen metagenome sequencing allows classifying and predicting feed efficiency and intake levels in cattle. *Sci. Rep.* **9**, 1–13 (2018).

3. Difford, G. F. *et al.* Host genetics and the rumen microbiome jointly associate with methane emissions in dairy cows. *PLOS Genet.* **14**, e1007580 (2018).

4. Weimer, P. J. Redundancy, resilience, and host specificity of the ruminal microbiota: Implications for engineering improved ruminal fermentations. *Front. Microbiol.* **6**, 296 (2015).

5. Knapp, J. R., Laur, G. L., Vadas, P. A., Weiss, W. P. & Tricarico, J. M. Invited review: Enteric methane in dairy cattle production: Quantifying the opportunities and impact of reducing emissions. *J. Dairy Sci.* **97**, 3231–3261 (2014).

6. Gerber *et al.* Tackling Climate Change Through Livestock. A global assessment of emissions and mitigation opportunities. 139 (2013) doi:www.fao.org/publications.

7. FAO. Global Livestock Environmental Assessment Model (GLEAM). *Food and Agriculture Organization* http://www.fao.org/gleam/results/en/ (2019).

8. Martínez-Álvaro, M. *et al.* Identification of Complex Rumen Microbiome Interaction Within Diverse Functional Niches as Mechanisms Affecting the Variation of Methane Emissions in Bovine. *Front. Microbiol.* **11**, 659 (2020).

9. Malmuthuge, N. & Guan, L. L. Understanding host-microbial interactions in rumen: searching the best opportunity for microbiota manipulation. *J. Anim. Sci. Biotechnol.* **8**, 8 (2017).

10. Negussie, E. *et al.* Invited review: Large-scale indirect measurements for enteric methane emissions in dairy cattle: A review of proxies and their potential for use in management and breeding decisions. *J. Dairy Sci.* **100**, 2433–2453 (2017).

11. Gloor, G. B., Macklaim, J. M., Pawlowsky-Glahn, V. & Egozcue, J. J. Microbiome datasets are compositional: And this is not optional. *Front. Microbiol.* **8**, 2224 (2017).

12. Liu, Y. X. *et al.* A practical guide to amplicon and metagenomic analysis of microbiome data. *Protein Cell* (2020) doi:10.1007/s13238-020-00724-8.

13. Bonk, F., Popp, D., Harms, H. & Centler, F. PCR-based quantification of taxa-specific abundances in microbial communities: Quantifying and avoiding common pitfalls. *J. Microbiol. Methods* **153**, 139–147 (2018).

14. Brandt, C., Bongcam-Rudloff, E. & Müller, B. Abundance Tracking by Long-Read Nanopore Sequencing of Complex Microbial Communities in Samples from 20 Different Biogas/Wastewater Plants. *Appl. Sci.* **10**, 7518 (2020).

15. Burki, F. *et al.* Phylogenomics Reshuffles the Eukaryotic Supergroups. *PLoS One* **2**, e790 (2007).

16. Kanehisa, M. & Goto, S. KEGG: Kyoto Encyclopedia of Genes and Genomes. *Nucleic Acids Res.* **28**, 27–30 (2000).

17. Kanehisa, M. Toward understanding the origin and evolution of cellular organisms. *Protein Sci.* **28**, 1947–1951 (2019).

18. Kanehisa, M., Sato, Y., Furumichi, M., Morishima, K. & Tanabe, M. New approach for understanding genome variations in KEGG. *Nucleic Acids Res.* **47**, D590–D595 (2019).

19. Lovell, D., Pawlowsky-Glahn, V., Egozcue, J. J., Marguerat, S. & Bähler, J. Proportionality: A Valid Alternative to Correlation for Relative Data. *PLOS Comput. Biol.* **11(3)**, e1004075, 1–12 (2015).

20. Wallace, R. J. *et al.* A heritable subset of the core rumen microbiome dictates dairy cow productivity and emissions. *Sci. Adv.* **5**, 1–12 (2019).

21. Shah, H. N. & Collins, D. M. Prevotella, a new genus to include Bacteroides melaninogenicus and related species formerly classified in the genus Bacteroides. *Int. J. Syst. Bacteriol.* **40**, 205–208 (1990).

22. Pitta, D. W. *et al.* Rumen bacterial diversity dynamics associated with changing from bermudagrass hay to grazed winter wheat diets. *Microb. Ecol.* **59**, 511–522 (2010).

23. Lee, H. J. *et al.* Comparative survey of rumen microbial communities and metabolites across one caprine and three bovine groups, using bar-coded pyrosequencing and 1H nuclear magnetic resonance spectroscopy. *Appl. Environ. Microbiol.* **78**, 5983–5993 (2012).

24. Lopes, L. D. *et al.* Exploring the sheep rumen microbiome for carbohydrate-active enzymes. *Antonie van Leeuwenhoek, Int. J. Gen. Mol. Microbiol.* **108**, 15–30 (2015).

25. Li, J. *et al.* A catalog of microbial genes from the bovine rumen unveils a specialized and diverse biomass-degrading environment. *Gigascience* **9**, 1–15 (2020).

26. Crespo-Piazuelo, D. *et al.* Characterization of bacterial microbiota compositions along the intestinal tract in pigs and their interactions and functions. *Sci. Rep.* **8**, 1–12 (2018).

27. Han, G. G. *et al.* Tracing of the fecal microbiota of commercial pigs at five growth stages from birth to shipment. *Sci. Rep.* **8**, 1–9 (2018).

28. Seshadri, R. *et al.* Cultivation and sequencing of rumen microbiome members from the Hungate1000 Collection. *Nat. Biotechnol.* **36**, 359–367 (2018).

29. Ransom-Jones, E., Jones, D. L., McCarthy, A. J. & McDonald, J. E. The Fibrobacteres: An Important Phylum of Cellulose-Degrading Bacteria. *Microb. Ecol.* **63**, 267–281 (2012).

30. Newbold, C. J., De La Fuente, G., Belanche, A., Ramos-Morales, E. & McEwan, N. R. The Role of Ciliate Protozoa in the Rumen. *Front. Microbiol.* **6**, 1–14 (2015).

31. Williams, A. G. & Coleman, G. S. *The Rumen Protozoa*. (Springer New York, 1992). doi:10.1007/978-1-4612-2776-2.

32. Johnson, H. P. A contribution to the morphology and biology of the Stentors. *J.*

Morphol. **8**, 467–562 (1893).

33. Tartar, V. *The Biology of Stentor*. (Pergamon Press, 1961). doi:10.1016/c2013-0-01654-4.

34. Wichterman, R. *The Biology of Paramecium*. (Springer US, 1986). doi:10.1007/978-1-4757-0372-6.

35. Cavalier-Smith, T. The neomuran origin of archaebacteria, the negibacterial root of the universal tree and bacterial megaclassification. *Int. J. Syst. Evol. Microbiol.* **52**, 7–76 (2002).

36. Poulsen, M. *et al.* Methylotrophic methanogenic Thermoplasmata implicated in reduced methane emissions from bovine rumen. *Nat. Commun.* **4**, 1–7 (2013).

37. Danielsson, R. *et al.* Methane Production in Dairy Cows Correlates with Rumen Methanogenic and Bacterial Community Structure. *Front. Microbiol.* **8**, 226 (2017).

38. Stewart, R. D. *et al.* Compendium of 4,941 rumen metagenome-assembled genomes for rumen microbiome biology and enzyme discovery. *Nat. Biotechnol.* **37**, 953–961 (2019).

39. Santos, E. de O. & Thompson, F. The family Succinivibrionaceae. in *The Prokaryotes: Gammaproteobacteria* (eds. Rosenberg, E., DeLong, E. F., Lory, S., Stackebrandt, E. & Thompson, F.) vol. 9783642389 639–648 (Springer, Berlin, Heidelberg, 2014).

40. Gomez-Gil, B. *et al.* The Family Vibrionaceae. in *The Prokaryotes: Gammaproteobacteria* (eds. Rosenberg, E., DeLong, E. F., Lory, S., Stackebrandt, E. & Thompson, F.) 659–747 (Springer, Berlin, Heidelberg, 2014). doi:10.1007/978-3-642-38922-1_225.

41. Ivanova, E. P., Ng, H. J. & Webb, H. K. The family Pseudoalteromonadaceae. in *The Prokaryotes: Gammaproteobacteria* (eds. Rosenberg, E., DeLong, E. F., Lory, S., Stackebrandt, E. & Thompson, F.) 575–582 (Springer, Berlin, Heidelberg, 2014). doi:10.1007/978-3-642-38922-1_229.

42. Marchandin, H. & Jumas-Bilak, E. The family Veillonellaceae. in *The Prokaryotes: Firmicutes and Tenericutes* (eds. Rosenberg, E., DeLong, E. F., Lory, S., Stackebrandt, E. & Thompson, F.) vol. 9783642301 433–453 (Springer, Berlin, Heidelberg, 2014).

43. Shah, H. N. & Collins, M. D. Reclassification of Bacteroides multiacidus (Mitsuoka, Terada, Watanabe and Uchida) in a new genus Mitsuokella, as Mitsuokella multiacidus comb. nov. *Zentralblatt fur Bakteriol. Angew. und Okol. Microbiol. Abt.1 Orig.C Hyg.* **3**, 491–494 (1982).

44. Shi, W. *et al.* Methane yield phenotypes linked to differential gene expression in the sheep rumen microbiome. *Genome Res.* **24**, 1517–1525 (2014).

45. Wallace, R. J. *et al.* Archaeal abundance in post-mortem ruminal digesta may help predict methane emissions from beef cattle. *Sci. Rep.* **4**, (2014).

46. Wallace, R. J. *et al.* The rumen microbial metagenome associated with high methane production in cattle. *BMC Genomics* **16**, 1–14 (2015).

47. Aguinaga Casañas, M. A. *et al.* Methyl-coenzyme M reductase A as an indicator to

estimate methane production from dairy cows. *J. Dairy Sci.* **98**, 4074–4083 (2015).

48. Belanche, A., de la Fuente, G. & Newbold, C. J. Study of methanogen communities associated with different rumen protozoal populations. *FEMS Microbiol. Ecol.* **90**, 663–677 (2014).

49. Janssen, P. H. & Kirs, M. Structure of the archaeal community of the rumen. *Appl. Environ. Microbiol.* **74**, 3619–3625 (2008).

50. Newbold, C. J., Lassalas, B. & Jouany, J. P. The importance of methanogens associated with ciliate protozoa in ruminal methane production in vitro. *Lett. Appl. Microbiol.* **21**, 230–234 (1995).

51. Qin, W. Z., Li, C. Y., Kim, J. K., Ju, J. G. & Song, M. K. Effects of defaunation on fermentation characteristics and methane production by rumen microbes in vitro when incubated with starchy feed sources. *Asian-Australasian J. Anim. Sci.* **25**, 1381–1388 (2012).

52. Schönhusen, U. *et al.* Effects of protozoa on methane production in rumen and hindgut of calves around time of weaning. *Arch. Anim. Nutr.* **57**, 279–295 (2003).

53. Morgavi, D. P., Martin, C., Jouany, J. P. & Ranilla, M. J. Rumen protozoa and methanogenesis: Not a simple cause-effect relationship. *Br. J. Nutr.* **107**, 388–397 (2012).

54. Guyader, J. *et al.* Influence of rumen protozoa on methane emission in ruminants: a meta-analysis approach. *Animal* **8**, 1816–1825 (2014).

55. Tapio, I., Snelling, T. J., Strozzi, F. & Wallace, R. J. The ruminal microbiome associated with methane emissions from ruminant livestock. *Journal of Animal Science and Biotechnology* vol. 8 1–11 (2017).

56. Hackstein, J. H. P. & de Graaf, R. M. Anaerobic Ciliates and Their Methanogenic Endosymbionts. in *(Endo)symbiotic Methanogenic Archaea* (ed. Hackstein, J. H. P.) 13–23 (Springer Berlin, 2018). doi:10.1007/978-3-319-98836-8_2.

57. Roopnarain, A., Mukhuba, M., Adeleke, R. & Moeletsi, M. Biases during DNA extraction affect bacterial and archaeal community profile of anaerobic digestion samples. *Biotech* **7:375**, 1–12 (2017).

58. Kittelmann, S. *et al.* Two Different Bacterial Community Types Are Linked with the Low-Methane Emission Trait in Sheep. *PLoS One* **9**, e103171 (2014).

59. Casadesús, J. & Low, D. Epigenetic Gene Regulation in the Bacterial World. *Microbiol. Mol. Biol. Rev.* **70**, 830–856 (2006).

60. Esquivel-Elizondo, S., Ilhan, Z. E., Garcia-Peña, E. I. & Krajmalnik-Brown, R. Insights into Butyrate Production in a Controlled Fermentation System via Gene Predictions. *mSystems* **2**, (2017).

61. Crable, B. R., Plugge, C. M., McInerney, M. J. & Stams, A. J. M. Formate formation and formate conversion in biological fuels production. *Enzyme Res.* **2011**, 1–8 (2011).

62. Hao, L. *et al.* Novel syntrophic bacteria in full-scale anaerobic digesters revealed by genome-centric metatranscriptomics. *ISME J.* **14**, 906–918 (2020).

63. Brown, T. D. K., Jones-Mortimer, M. C. & Kornberg, H. L. The enzymic interconversion of acetate and acetyl-coenzyme A in Escherichia coli. *J. Gen. Microbiol.* **102**, 327–336 (1977).

64. Xie, M., Chen, H. H., Nie, S. P., Yin, J. Y. & Xie, M. Y. Gamma-aminobutyric acid increases the production of short-chain fatty acids and decreases pH values in mouse colon. *Molecules* **22**, (2017).

65. Graham, D. E. 2-oxoacid metabolism in methanogenic CoM and CoB biosynthesis. in *Methods in Enzymology* vol. 494 301–326 (Academic Press Inc., 2011).

66. Klüber, H. D. & Conrad, R. Effects of nitrate, nitrite, NO and N2O on methanogenesis and other redox processes in anoxic rice field soil. *FEMS Microbiol. Ecol.* **25**, 301–318 (2006).

67. Yang, C., Rooke, J. A., Cabeza, I. & Wallace, R. J. Nitrate and inhibition of ruminal methanogenesis: Microbial ecology, obstacles, and opportunities for lowering methane emissions from ruminant livestock. *Front. Microbiol.* **7**, 132 (2016).

68. Lee, C. & Beauchemin, K. A. Une revue de l'ajout de nitrate dans l'alimentation des ruminants : Toxicité aux nitrates, émissions de méthane et performance de production. *Can. J. Anim. Sci.* **94**, 557–570 (2014).

69. Cui, X. The bacteriocidal effects of transition metal complexes containing the NO+ group on the food-spoilage bacterium Clostridium sporogenes. *FEMS Microbiol. Lett.* **98**, 67–70 (1992).

70. Zumft, W. G. The biological role of nitric oxide in bacteria. *Arch. Microbiol.* **160**, 253–264 (1993).

71. Reed, B. *et al.* Alterations in mammalian cells induced by inactivation of vitamin B12 with nitrous oxide. in *Vitamin B12* (eds. Zagalak, B. & Friedrich, W.) 1061–1064 (De Gruyter, 1979). doi:10.1515/9783111510828-120.

72. Klüber, H. D. & Conrad, R. Inhibitory effects of nitrate, nitrite, NO and N2O on methanogenesis by Methanosarcina barkeri and Methanobacterium bryantii. *FEMS Microbiol. Ecol.* **25**, 331–339 (1998).

73. Rey, J. *et al.* Comparison Between Non-Invasive Methane Measurement Techniques in Cattle. *Animals* **9**, 1–9 (2019).

74. López-Paredes, J. *et al.* Mitigation of greenhouse gases in dairy cattle via genetic selection: 1. Genetic parameters of direct methane using noninvasive methods and proxies of methane. *J. Dairy Sci.* **103**, 7199–7209 (2020).

75. Tamames, J. & Puente-Sánchez, F. SqueezeMeta, A Highly Portable, Fully Automatic Metagenomic Analysis Pipeline. *Front. Microbiol.* **9**, 1–10 (2019).

76. Aitchison, J. The Statistical Analysis of Compositional Data. *J. R. Stat. Soc. Ser. B* **44**, 139–160 (1982).

77. Greenacre, M. Package 'easyCODA'. Compositional Data Analysis in Practice. 35 (2019).

78. Palarea-Albaladejo, J. & Martín-Fernández, J. A. ZCompositions - R package for

multivariate imputation of left-censored data under a compositional approach. *Chemometrics and Intelligent Laboratory Systems* vol. 143 85–96 (2015).

79. Oksanen, J. *et al.* vegan: Community Ecology Package. R package version 2.5-6. (2020).

80. Anderson, M. J. A new method for non-parametric multivariate analysis of variance. *Austral Ecol.* **26**, 32–46 (2001).

81. McArdle, B. H. & Anderson, M. J. Fitting multivariate models to community data: A comment on distance-based redundancy analysis. *Ecology* **82**, 290–297 (2001).

82. Ritchie, M. E. *et al.* limma powers differential expression analyses for RNA-sequencing and microarray studies. *Nucleic Acids Res.* **43**, (2015).

83. Quinn, T. P., Richardson, M. F., Lovell, D. & Crowley, T. M. Propr: An R-package for Identifying Proportionally Abundant Features Using Compositional Data Analysis. *Sci. Rep.* **7**, 1–9 (2017).

84. Erb, I. & Notredame, C. How should we measure proportionality on relative gene expression data? *Theory Biosci.* **135**, 21–36 (2016).

85. Kamada, T. & Kawai, S. An algorithm for drawing general undirected graphs. *Inf. Process. Lett.* **31**, 7–15 (1989).

16 Tables

Table 1: F statistic and P-values for stage of lactation (SL), number of lactation (NL) and methane emission (CH4) variables (added sequentially) and P-values from PERMANOVA of the entire dataset (i.e., including all superkingdoms).

		F statistic	R^2	*P*-value
	SL	6.1	0.014	<0.01*
Phylum	*NL*	1.4	0.003	0.11
	CH4	2.8	0.019	<0.01*
	SL	5.6	0.013	<0.01*
Class	*NL*	1.5	0.003	0.07
	CH4	2.4	0.016	<0.01*
	SL	5.4	0.012	<0.01*
Order	*NL*	1.7	0.004	0.03*
	CH4	2.3	0.016	<0.01*
	SL	4.9	0.011	<0.01*
Family	*NL*	1.6	0.004	0.03*
	CH4	2.1	0.014	<0.01*
	SL	4.0	0.009	<0.01*
Genus	*NL*	1.4	0.003	0.03*
	CH4	1.7	0.012	<0.01*
	SL	5.3	0.012	<0.01*
KEGG	*NL*	2.0	0.004	0.02*
	CH4	2.4	0.016	<0.01*

**P-value* < 0.05

17 Figure captions

Figure 1: **Feature counts distribution**. Features with zero counts, singletons, doubletons and 3 or more counts per sample in the final taxonomy table (A) and in the final functionality table (B).

Figure 2: Average relative abundance of genera. A) All features, including those classified only to family level (i.e., unclassified genera); B) Features classified to genus level (unclassified genera removed).

Figure 3: **Phyla relative abundance per sample**. Samples are sorted from lowest to highest methane emissions.

Figure 4: **Metagenome functionality**. TreeMap distribution of functionality abundances classified as KEGG pathways (left) and BRITE hierarchies (right) associated with core KEGG subcomposition.

Figure 5. Fitted surface representation of Principal Component Analysis. Dots represent the samples using euclidean distances of CLR-transformed taxa abundances, colored by CH4 levels. CH_4 emissions (ppm) corrected by number and stage of lactation are represented as smooth fitting following a generalized additive model (GAM) (–). *Dev. Explained*: variability explained by GAM; *P-val*: approximate significance of the smooth terms being zero (α=0.05).

Figure 6. Volcano plots. Volcano plot representing the differential abundance (DA) of genera (A) and KEGGs (B) between LOW and HIGH groups from limma. Significance thresholds were established at *adj.P-val* = 0.05 and $\log_2$FC = ±0.5. • Significant features with DA above the fold change (FC) threshold. • Significant features with DA below the

FC threshold. • Non-significant features with DA above the FC threshold. • Non-significant features with DA below the FC threshold.

Figure 7. **Taxonomy interaction network**. Pairwise proportionalities between genera with $|\rho_p| \geq 0.4$. Superkingdom: △ Archaea; □ Bacteria; ○ Eukaryota. / CH_4 association: ▬ HIGH CH_4; ▬ LOW CH_4; ▬ No CH_4 associated. / Proportionality sense: ↔ direct (> 0); ↔ inverse (< 0).

Figure 8. Functionality interaction network. Presented pairwise proportionalities between KEGGs with $|\rho_p| \geq 0.7$/ Participation in methane metabolism: □ ko00680 (direct or indirect part.); ○ Other (no part.) / CH_4 association: ▬ HIGH CH_4; ▬ LOW CH_4; ▬ No CH_4 associated. / Proportionality sense: ↔ direct (> 0); ↔ inverse (< 0). Clusters are indicated as L (KEGGs associated to LOW methane), H (KEGGs associated to HIGH methane) and N (KEGGs not related to methane emissions).

Figure 9: **Taxonomy of ko00680 KEGGs**. Relative abundance of KEGGs present in ko00680 pathway for each phylum in Archaea (▬), Bacteria (▬) and Eukaryota (▬) superkingdoms. Relative abundance of each ko00680-KEGG respect to the sum of reads mapped to all ko00680-KEGGs.

Figure 10. Taxonomic distribution of DA KEGGs. Red density scale represents KEGGs over-abundant (OA) in HIGH emitters; Blue density scale represents KEGGs OA in LOW emitters. More intense colors mean a higher number of reads assigned to one phylum. Superkingdom: • Archaea; • Bacteria; • Eukaryota.

Figure 1

Click here to access/download;Figure;Fig1.tif

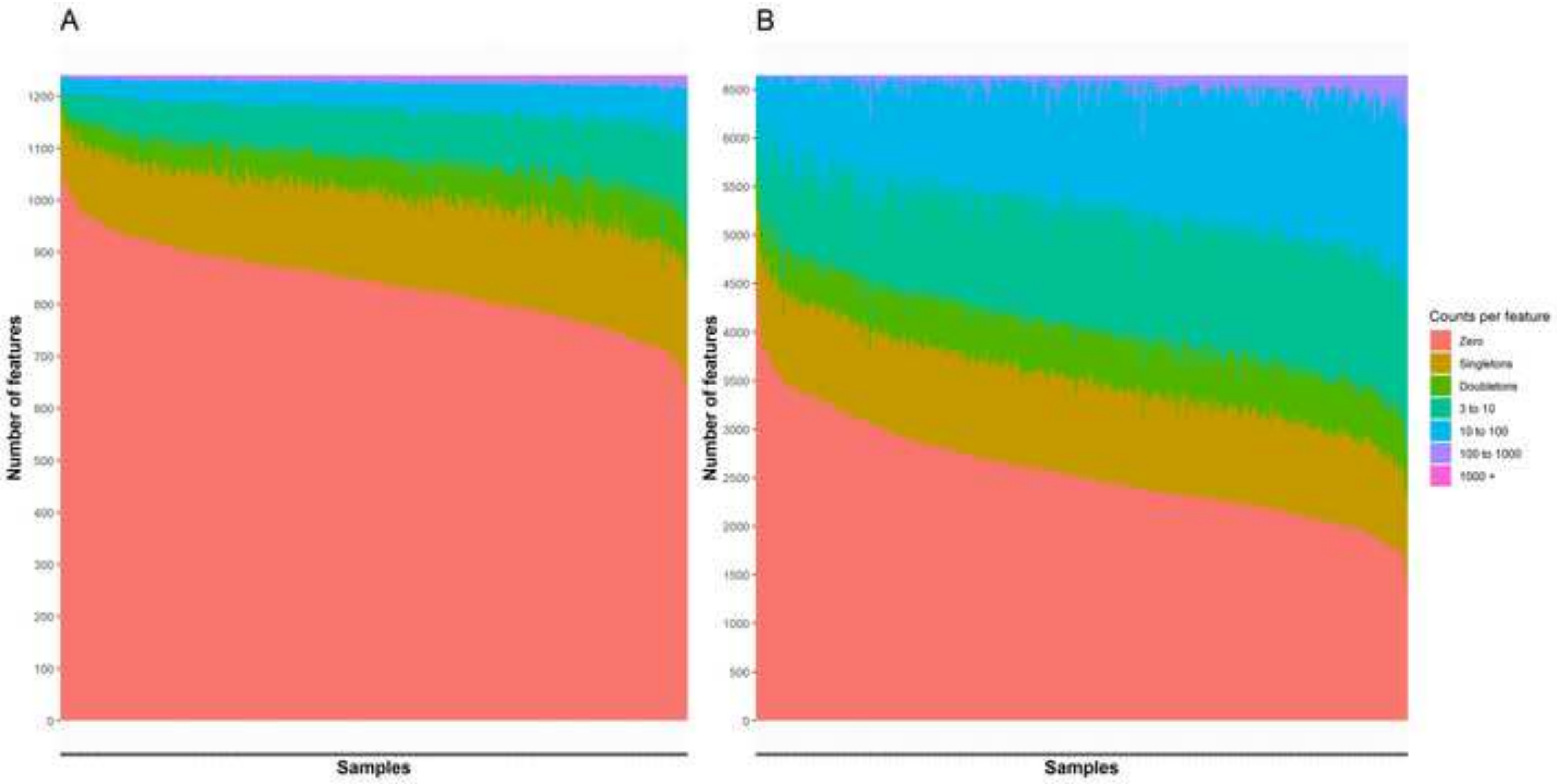

Figure 2

Click here to access/download;Figure;Fig2.tif

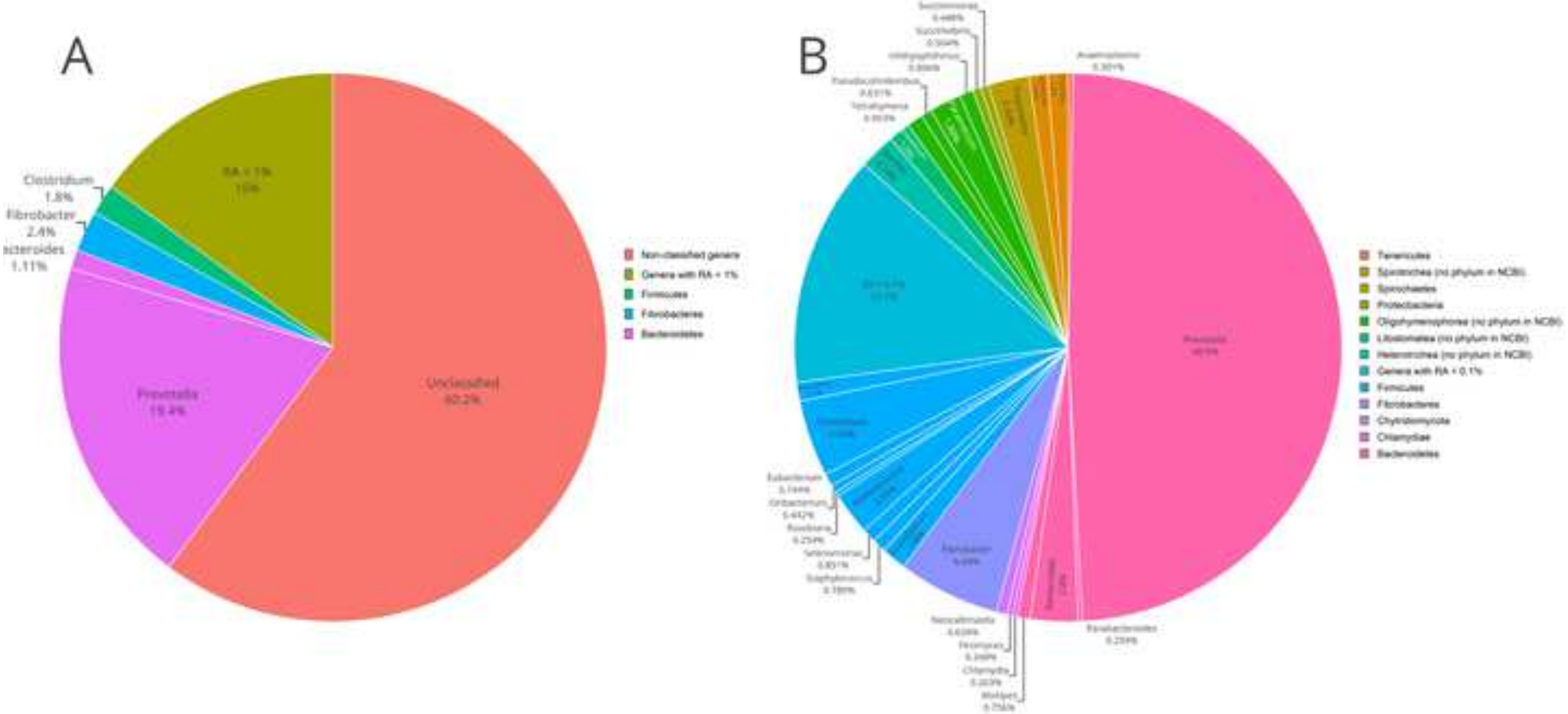

Figure 3

Click here to access/download;Figure;Fig3.tif

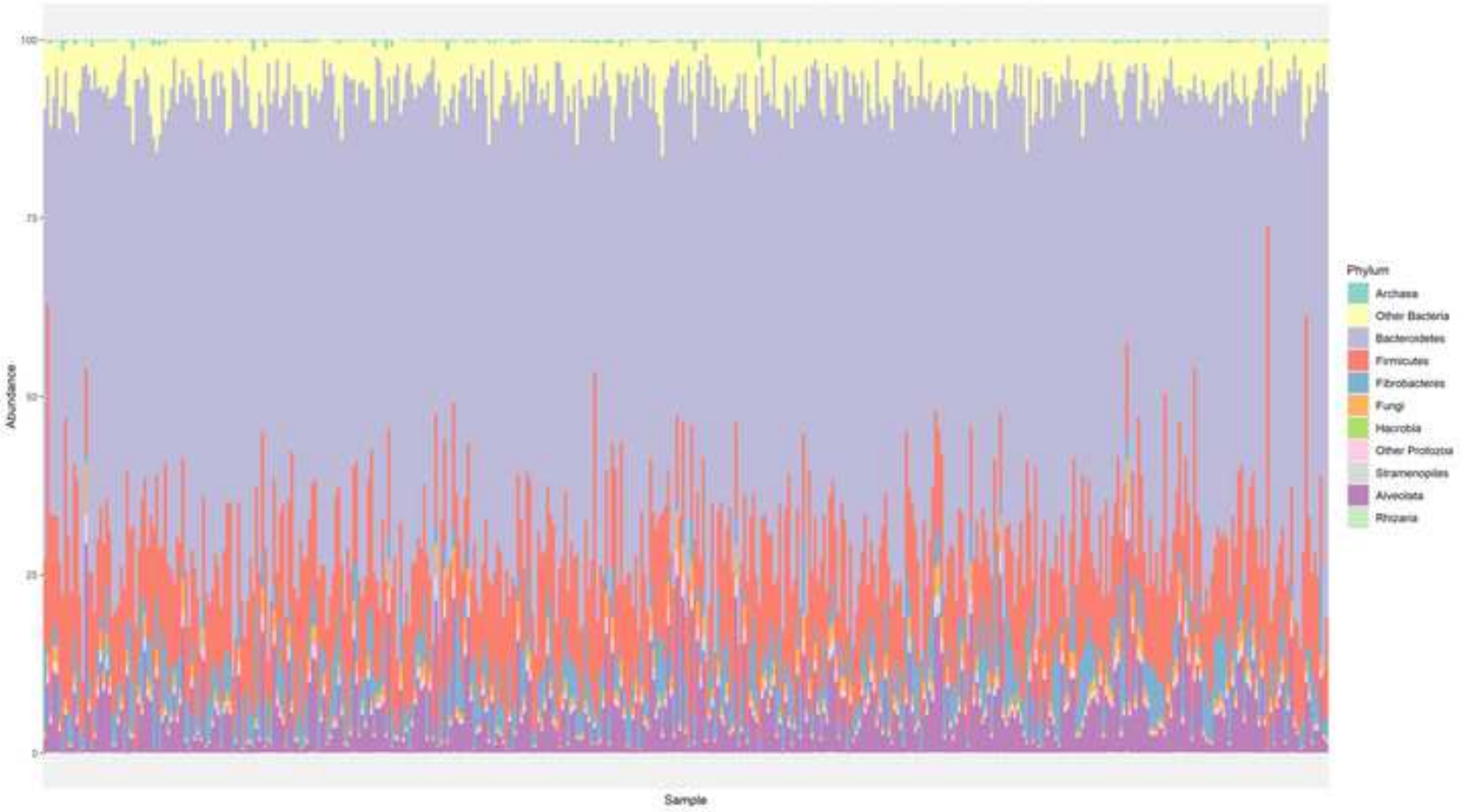

Figure 4

Click here to access/download;Figure;Fig4.tif

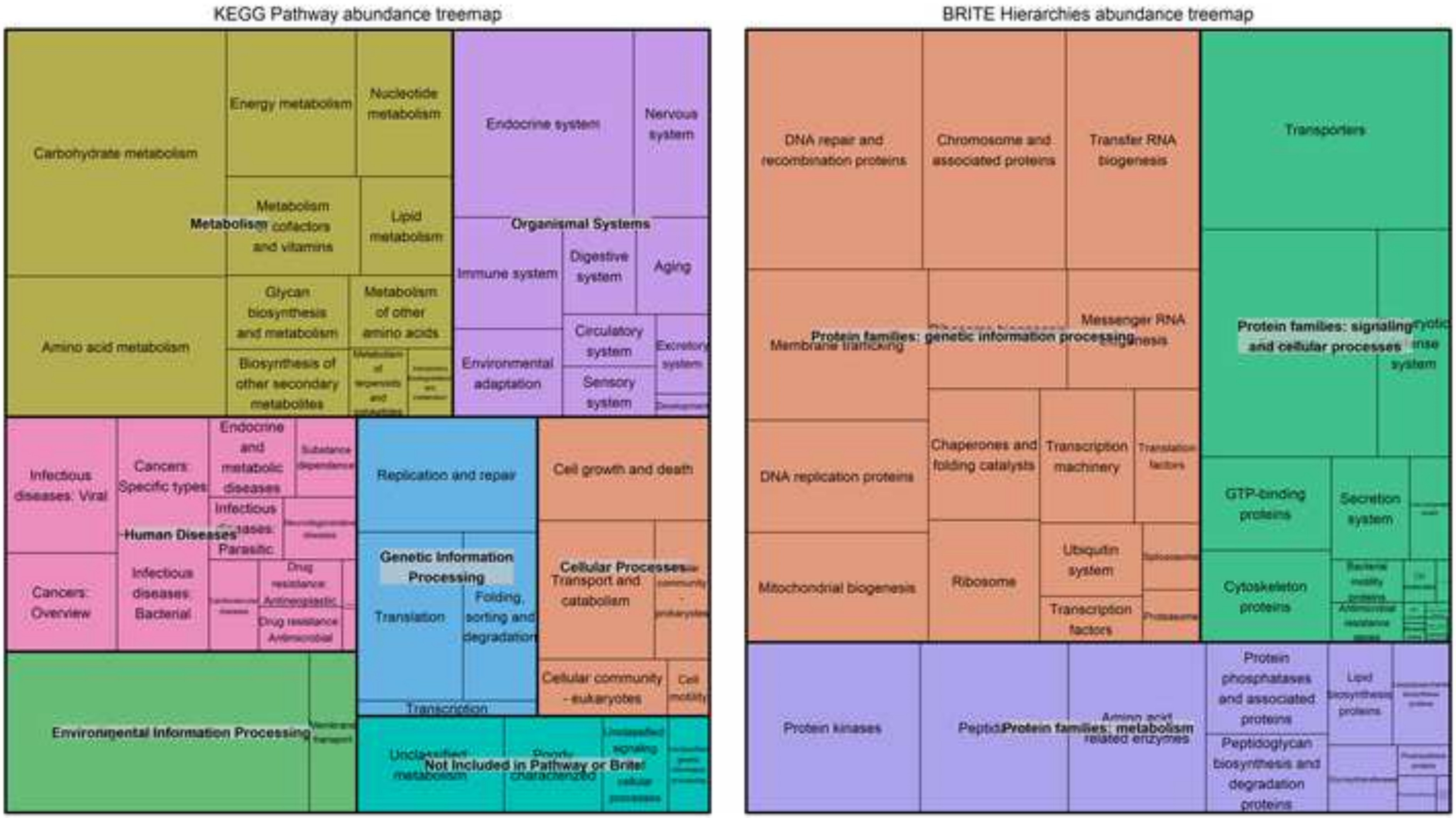

Figure 5

Click here to access/download;Figure;Fig5.tif

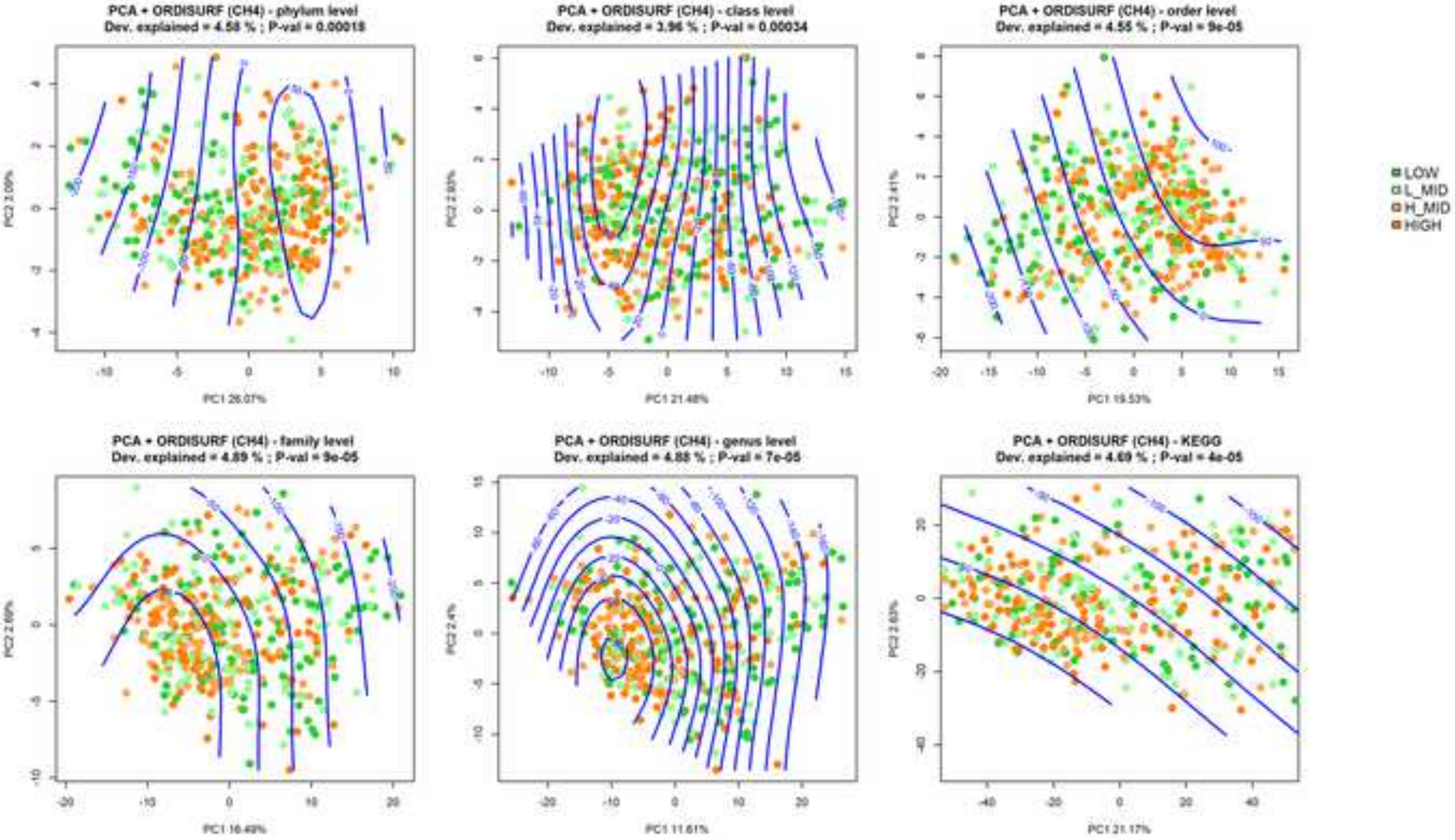

Figure 6

Click here to access/download;Figure;Fig6.tif

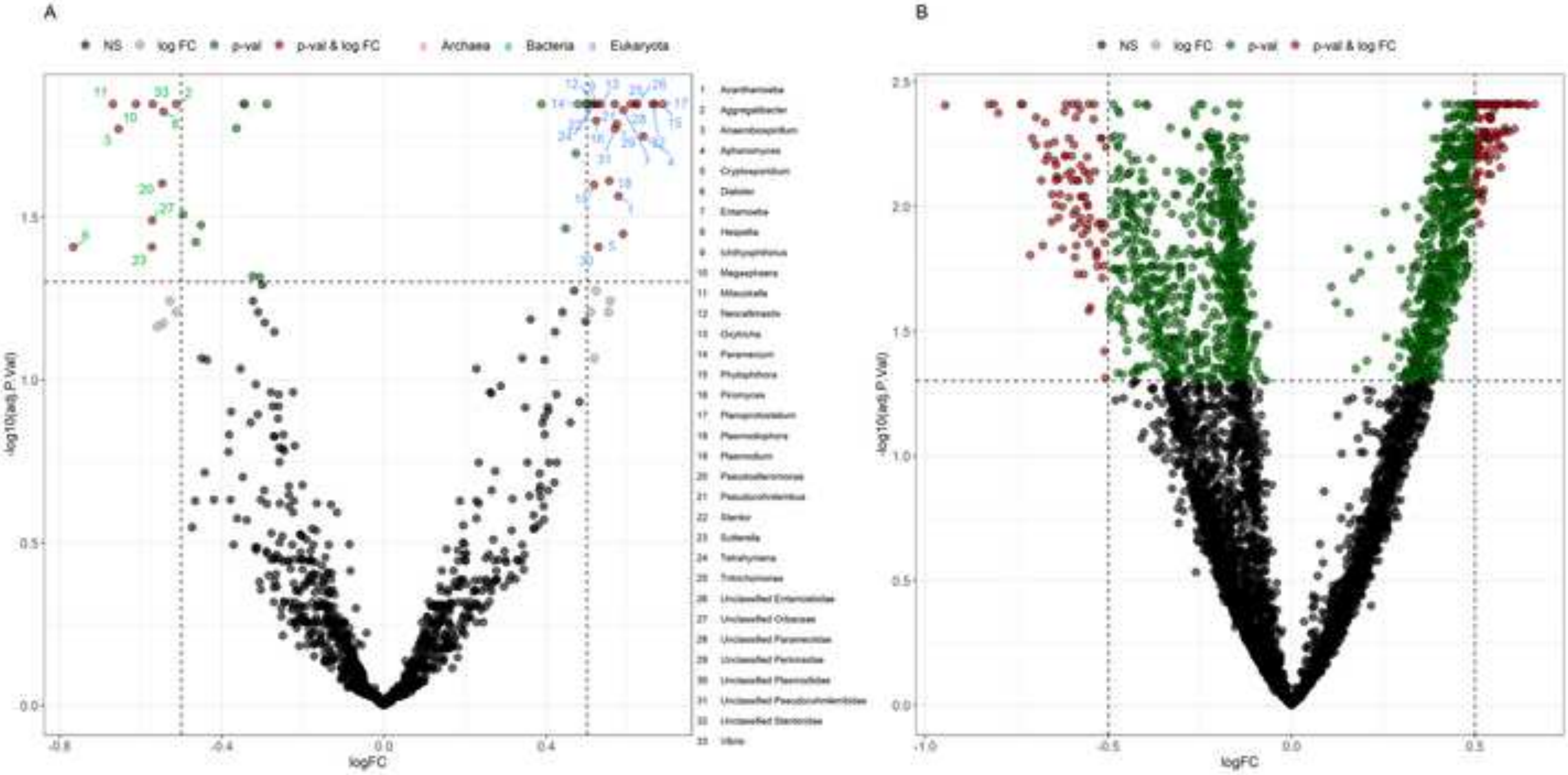

Figure 7

Click here to access/download;Figure;Fig7.tif

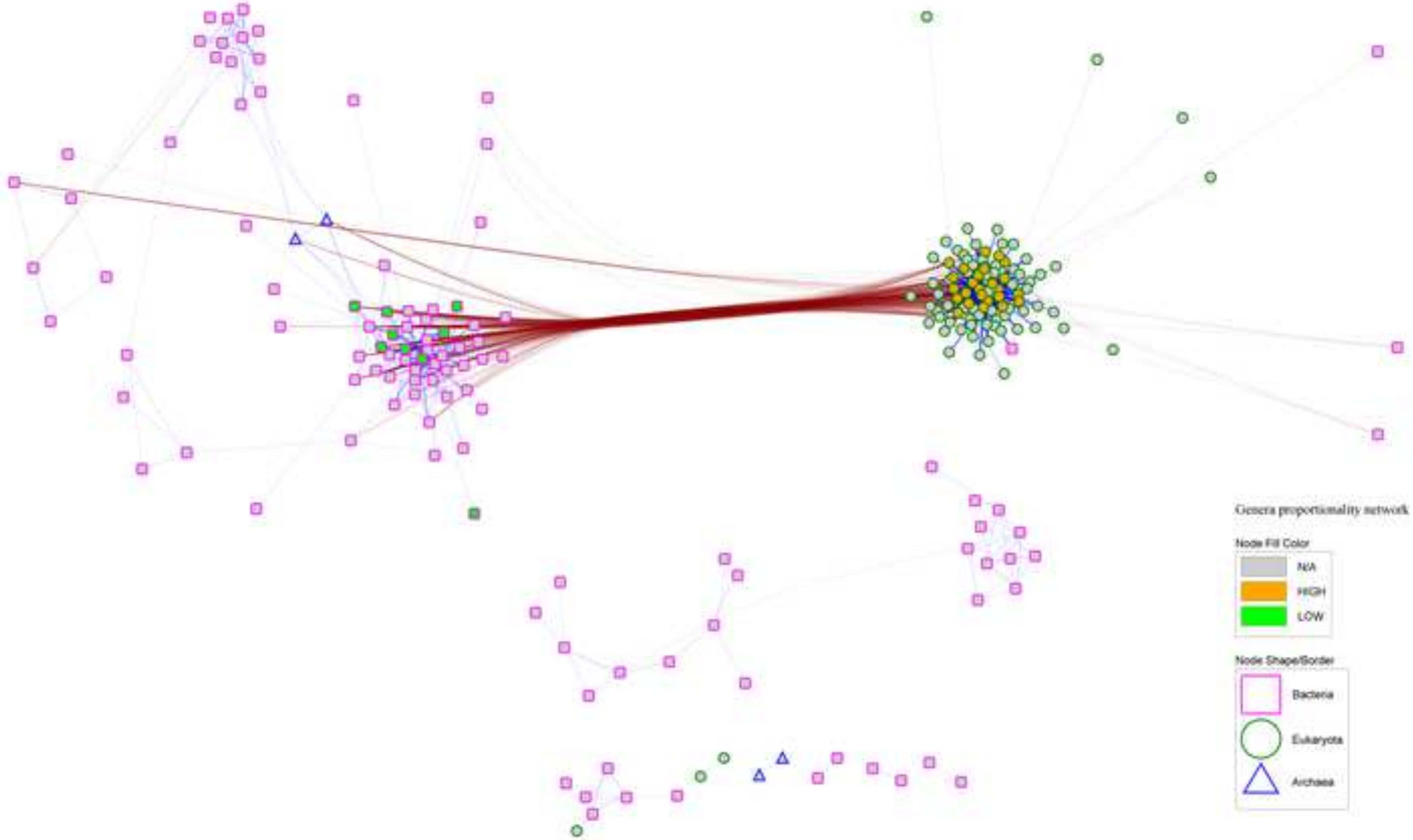

Figure 8

Click here to access/download;Figure;Fig8.tif

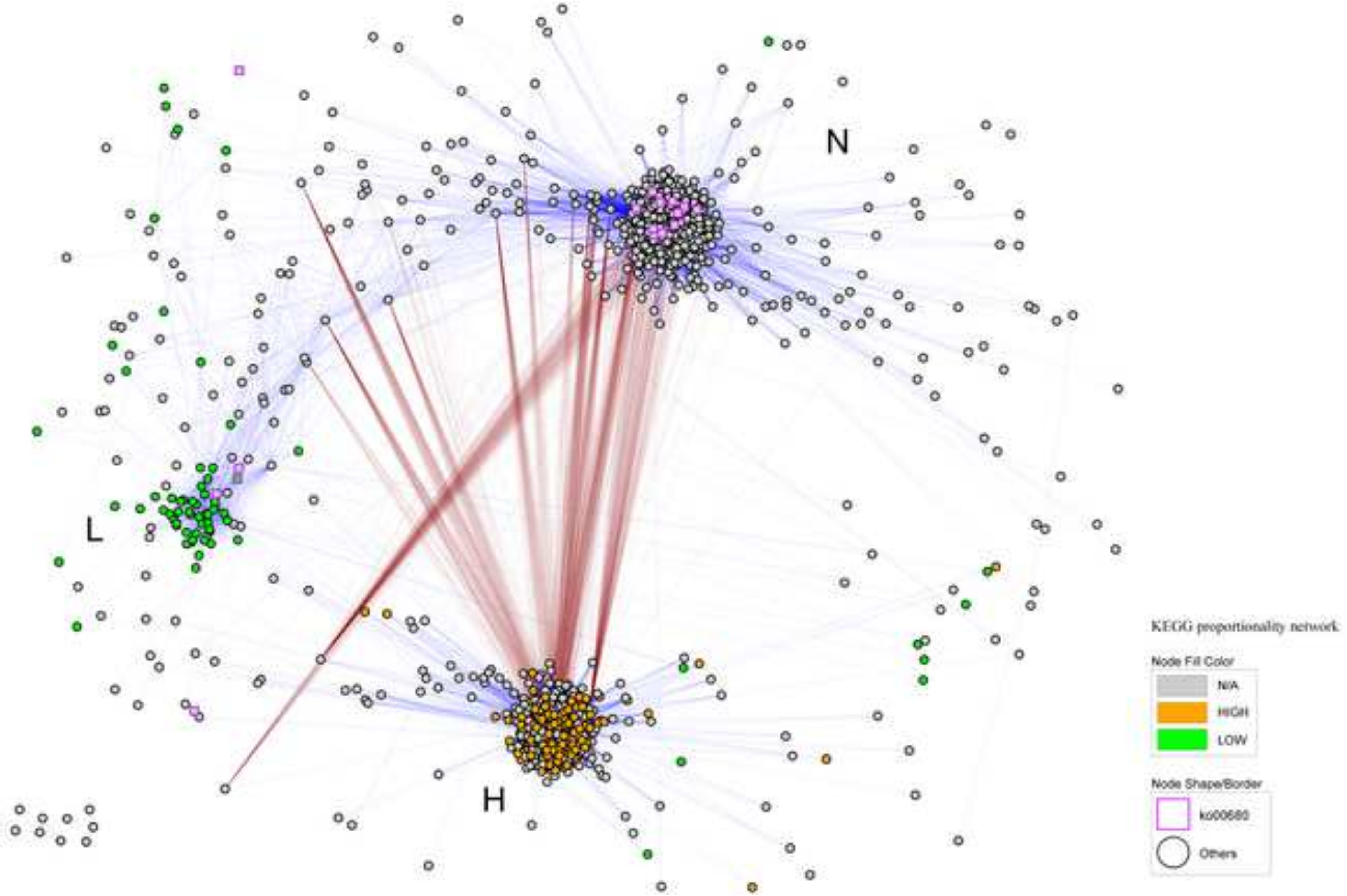

Figure 9

Click here to access/download;Figure;Fig9.tif

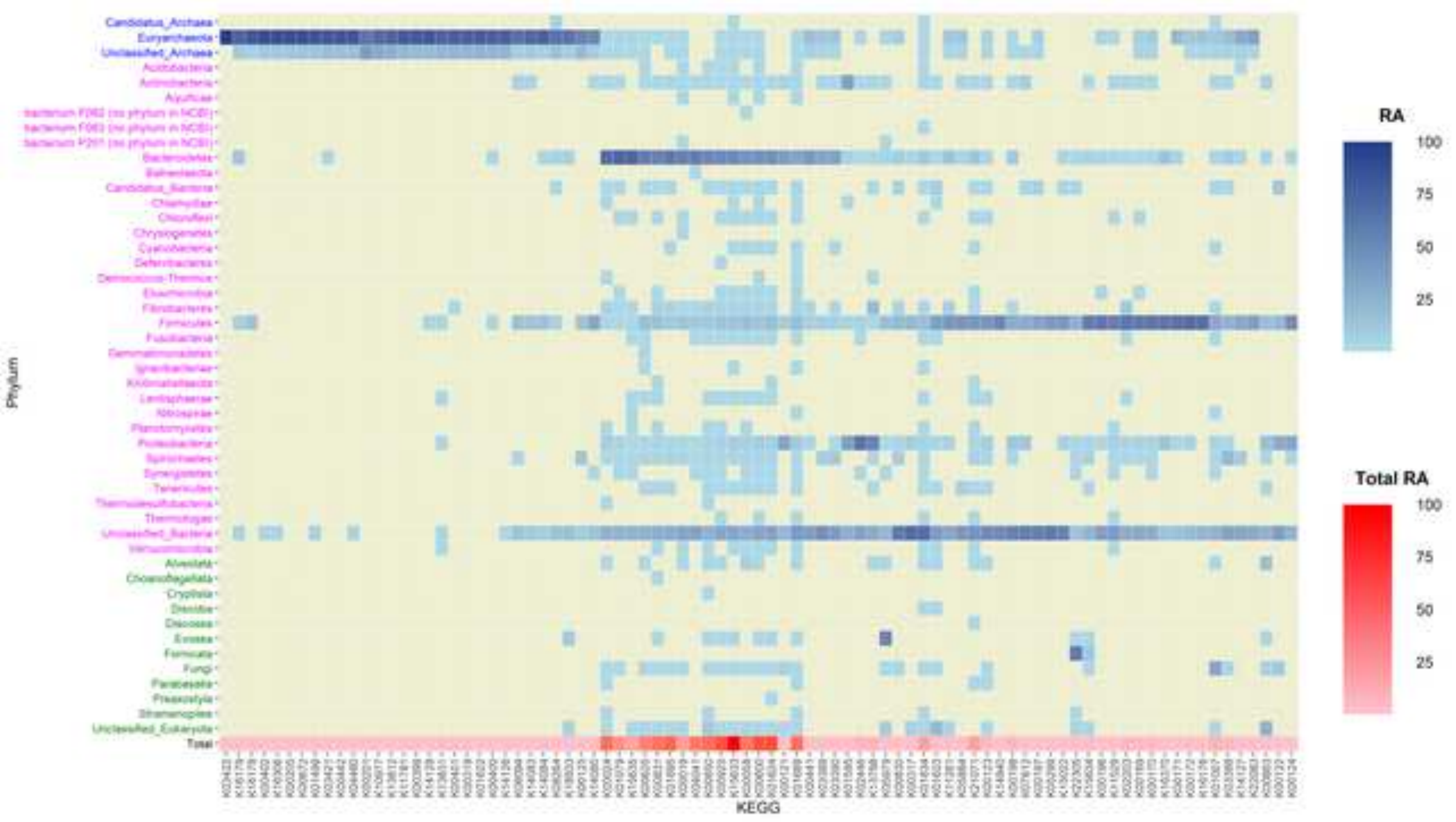

Figure 10

Click here to access/download;Figure;Fig10.tif

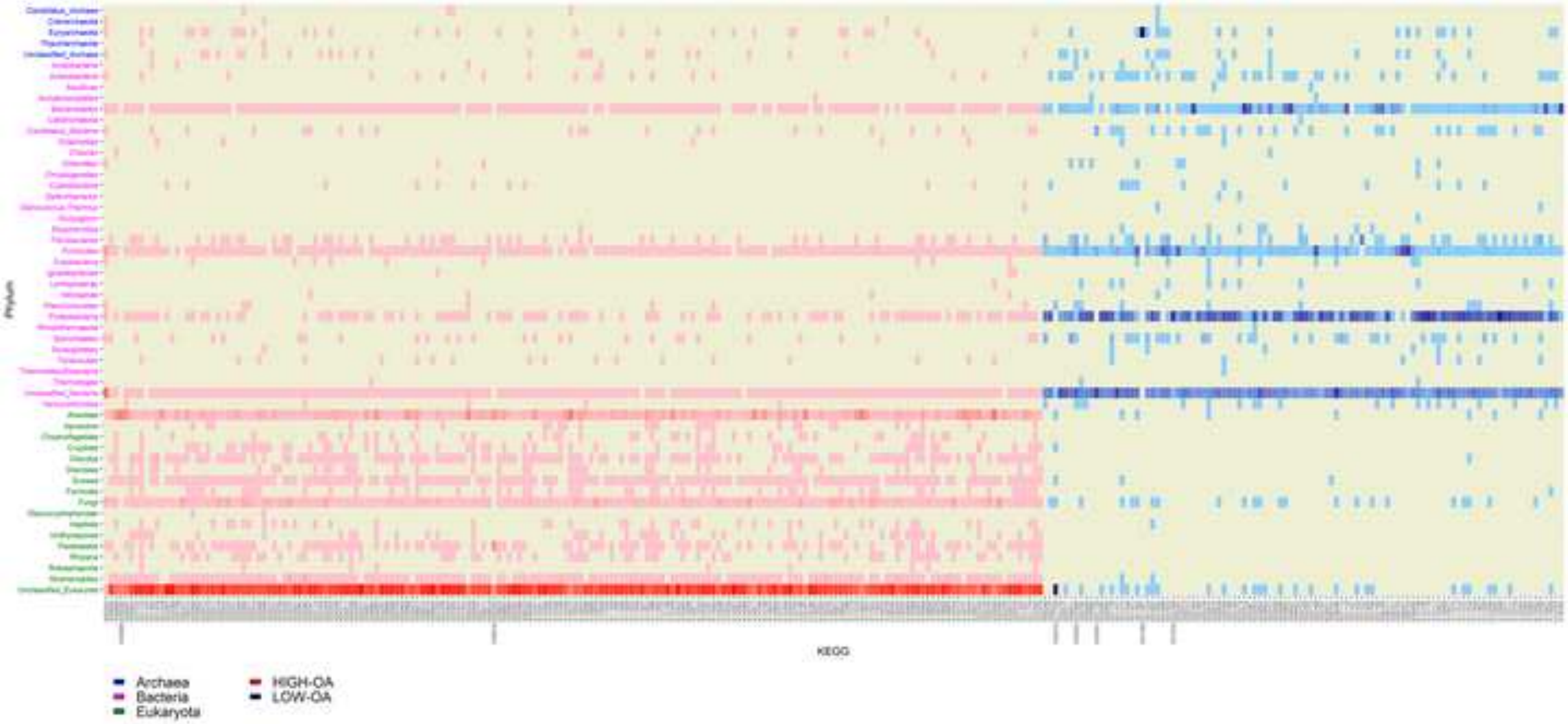

Additional file 1

Click here to access/download

Supplementary Material

Additional_file_1.xlsx

Additional file 2

Click here to access/download

Supplementary Material

Additional_file_2.docx

Cover Letter

Click here to access/download;Personal Cover;Cover_letter.docx

GOBIERNO DE ESPAÑA

INIA
Instituto Nacional de Investigación y Tecnología Agraria y Alimentaria

DEPARTAMENTO DE MEJORA GENETICA ANIMAL

Madrid, 02nd august, 2021

Dear Editor,

herewith, we submit our manuscript entitled "FUNGAL AND CILIATE PROTOZOA ARE THE MAIN RUMEN MICROBES ASSOCIATED WITH METHANE EMISSIONS IN DAIRY CATTLE", to be considered for publication in GigaScience.

The manuscript we are enclosing is a full research paper on animal microbiome, which reports results of an experiment performed using 437 Holstein cows. We explored the differences in the ruminal microbiota composition between cows with different methane emission levels, using nanopore technologies for long-reads metagenome sequencing and subsequent compositional data analysis. The uniqueness of the current research is that we generated one of the largest nanopore long-read ruminal metagenome datasets currently available, providing a comprehensive approach to rumen microbial ecosystem, accounting representative microbial taxonomy within all superkingdoms, as well as their potential metabolic activity related to methane emissions. Our results contribute to a better understanding of the complex interactions between rumen microbiota and host phenotype.

All authors are aware of the paper submission and agreed to be listed as co-authors, as affirmed by my signature as corresponding author, and there is no conflict of interest that would prejudice the information offered in the paper.

It is our understanding that this manuscript clearly fits in the journal scope and that it provides new, coherent, and sound addition to scientific knowledge. We sincerely hope you will find the paper a useful contribution to the field and adequate for publication in the journal.

Data are already uploaded in ENA, and they will be made available upon acceptance of the study.

Thank you in advance for considering this manuscript.

Sincerely,

Dr. Óscar González-Recio
Senior Research Scientist,
SGIT – INIA

Adrián López-García
PhD student,
SGIT – INIA

CORREO ELECTRÓNICO:
adrian.lopez@inia.es

Ctra. de La Coruña, Km. 7,5
28040 MADRID
TEL: 91 347 14 92
FAX: 91 347 87 43
