# Supplementary material for: Fungal and ciliate protozoa are the main rumen microbes associated with methane emissions in dairy cattle
Source: Gigascience. 2022 Jan 25;11:giab088. doi: 10.1093/gigascience/giab088 (PMC8848325; doi:10.1093/gigascience/giab088)
Supplement: giab088_GIGA-D-21-00239_Revision_1 [file giab088_giga-d-21-00239_revision_1.pdf]

GigaScience

Fungal and ciliate protozoa are the main rumen microbes associated with methane emissions in dairy cattle

--Manuscript Draft--

**Manuscript Number:**	GIGA-D-21-00239R1
**Full Title:**	Fungal and ciliate protozoa are the main rumen microbes associated with methane emissions in dairy cattle
**Article Type:**	Research
**Funding Information:**	Ministerio de Ciencia, Innovación y Universidades (RTI2018-096487-RC33) — Not applicable<br>Ministerio de Economía y Competitividad (RTA2015-00022-C03-02) — Not applicable<br>INIA: Instituto Nacional de Investigacion y Tecnologia Agraria y Alimentaria (reference FPI-SGIT2016-06) — Mr Adrián López-García
**Abstract:**	Background: Mitigating the effects of global warming has become the main challenge for humanity in the last decades. Livestock farming contributes to greenhouse gas emissions, with an important output of methane from enteric fermentation processes, mostly in ruminants. As ruminal microbiota is directly involved in digestive fermentation processes and methane biosynthesis, understanding the ecological relationships between rumen microorganisms and their active metabolic pathways is essential for reducing emissions. This study analyzed whole rumen metagenome using long reads and considering its compositional nature in order to disentangle the role of rumen microbes in methane emissions.<br>Results: The beta-diversity analyses suggested an association between methane production and overall microbiota composition (0.01 < R2 < 0.02). Differential abundance analysis identified 36 genera and 279 KEGGs as significantly associated to methane production (Padj<0.05). Those genera associated to high methane production were Eukaryota from Alveolata and Fungi clades, while Bacteria were associated to low methane emissions. The genus-level association network showed two clusters grouping Eukaryota and Bacteria, respectively. Regarding microbial gene functions, 41 KEGGs resulted to be differentially abundant between low and high emission animals, and were mainly involved in metabolic pathways. No KEGGs included in the methane metabolism pathway (ko00680) were detected as associated to high methane emissions. The KEGG network showed three clusters grouping KEGGs associated to high emissions, low emissions and not differentially abundant in either of them. A deeper analysis of the differentially abundant KEGGs revealed that genes related with anaerobic respiration through nitrate degradation were more abundant in low emissions animals.<br>Conclusions: Methane emissions are largely associated to the relative abundance of ciliate and fungi. The role of nitrate electron acceptors can be particularly important as this respiration mechanism directly competes with methanogenesis. Therefore, whole metagenome sequencing is necessary to jointly consider relative abundance of Bacteria, Archaea and Eukaryota in the statistical analyses. Nutritional and genetic strategies to reduce CH4 emissions should focus on reducing the relative abundance of Alveolata and Fungi in the rumen. This experiment has generated the largest ONT ruminal metagenomic dataset currently available.
**Corresponding Author:**	Oscar Gonzalez-Recio<br>INIA: Instituto Nacional de Investigacion y Tecnologia Agraria y Alimentaria<br>Madrid, Ma SPAIN
**Corresponding Author Secondary Information:**	
**Corresponding Author's Institution:**	INIA: Instituto Nacional de Investigacion y Tecnologia Agraria y Alimentaria
**Corresponding Author's Secondary Institution:**	

Powered by Editorial Manager® and ProduXion Manager® from Aries Systems Corporation

**First Author:**	Adrián López-García
**First Author Secondary Information:**	
**Order of Authors:**	Adrián López-García
	Alejandro Saborío-Montero
	Mónica Gutiérrez-Rivas
	Raquel Atxaerandio
	Idoia Goiri
	Aser García-Rodríguez
	José A. Jiménez-Montero
	Carmen González
	Javier Tamames
	Fernando Puente-Sánchez
	Magdalena Serrano
	Rafael Carrasco
	Cristina Óvilo
	Oscar Gonzalez-Recio
**Order of Authors Secondary Information:**	
**Response to Reviewers:**	Reviewer #1:<br>The manuscript by Lopez-Garcia et al "Fungal and ciliate protozoa are the main rumen microbes associated with methane emissions in dairy cattle" describes a study where a large dataset of nanopore data was generated from rumen samples. It clearly describes the various steps that were taken to analyse the data. Various aspects of importance were taken into consideration such as abundance normalisation, and significance adjustment for multiple hypothesis testing. This study gives insights into previously unknown correlations between methane emission and taxa (particularly eukaryotic organisms), and between methane emission and genes.<br>Provided that some suggestions are implemented, this research paper should be considered for publication.<br>Minor comments:<br>Figures: almost all figures need some improvement:<br>All figures: axes titles font, and particularly, axes labels font needs to be larger.<br>All figures should be pdf/vectorised files. I (reviewer) see them all pixelated and I was not able to read most of the words.<br>AU: Thank you for your comments, we have considered them carefully. They were really useful and helped improving the manuscript in a nice manner. We would like to point out that the journal’s built-in pdf shows the figures in lower quality than the expected, but downloading them makes possible to visualize them with high quality. We believe that the format of the figures will be adapted during the editing process keeping the high quality of the figures provided.<br>Figure 1: this figure could be removed as it is little informative. If kept, a title on top of each panel could be added to highlight the different nature of the features.<br>AU: Thank you. This figure has been moved to supplementary material and completed with titles and pre-filtering count distribution. New Fig.: Supplementary Figure 1.<br>Figure 2: 2A is little informative. I suggest keeping 2B, but visualized as a treemap instead, much more readable than a pie chart.<br>AU: Changes applied. New Fig.: Figure 1.<br>Figure 3: is there a message in this figure that has not already been made broadly explicit in the manuscript? If not, I suggest removing it or keep it as a supplementary figure.<br>AU: We believe that this figure visualizes nicely what is described in the text. However, we moved it to supplementary material and re-ordered samples by increasing RA of Bacteroidetes for a better difference appreciation. Please see new Fig.: Supplementary Figure 2.<br>A figure to report the core microbiome composition (bact, euk, and arch) as reported

Powered by Editorial Manager® and ProduXion Manager® from Aries Systems Corporation

	between line 131-138 would be more informative than the current figures 1,2A, and 3.<br>AU: The composition percentages in the text are the ones represented in the former figure.<br>Figure 6: unreadable text right of 6A.<br>AU: The font size was slightly increased, but as said before, downloading the figures might allow to visualize it correctly. Please see new Fig.: Figure 4.<br>Figure 7: there are node fill colours that do not match the colours shown in the legend. Shouldn't the node fill colours be orange, green, grey (high, low, ns)?<br>AU: The nodes are filled correctly. Not every node in the clusters belong to the HIGH or LOW categories, as significant DA was not detected for every represented genus, but every HIGH or LOW node belong to the same cluster. Note that co-abundance proportionalities (i.e., edges) range from 0.4 to 1 in absolute values, as stated in methods (line 552), so edges connecting an orange node (i.e., over-abundant in HIGH group) and a grey node (i.e., not significantly DA: p-val > 0.05 & | log2FC | < 0.5) might be co-abundant but in a lesser grade. Please see new Fig.: Figure 5.<br>Line 46: differentially abundant between low and high emission animals? Needs to be made explicit.<br>AU: Changes applied (Line 44).<br>Line 124: RA acronym has not been introduced before<br>AU: Change applied by adding a sentence before the first RA occurrence (line 117).<br>Line 143: What is the N50 of these reads? N50 and L50 could be reported.<br>AU: We added N50 values for both taxonomy-mapped and KEGG-mapped reads (lines 107 and 138).<br>Line 149: Could "most" here be made explicit in numbers (i.e. percentages)?<br>AU: Changes applied (line 143): “A 26% of the rumen metagenome functions […]”.<br>Line 150: Explicit what falls within cellular generic processes (or sub groups here of) in numbers (i.e. percentages)?<br>AU: Changes applied by adding percentages to each type of cellular process (lines 145-147).<br>Line 155: PCA acronym first time appearance; needs introduction.<br>AU: Change applied.<br>Line 170: I assume p-values in Table 1 have been adjusted for FDR with the BH method as described in the methods. If not, adjusted p-values should (also) be reported. Also, p-value adjustment method could be mentioned either here or in the Table caption.<br>AU: In this case, each PERMANOVA (phylum, class, order, etc.) was run separately with differently grouped datasets. Note that in each analyses the dependent variable are the microbiota composition dissimilarities between samples, at each respective taxonomic level. Independent variables are only three, (SL, NL and CH4), so BH correction is not required. P-values are outputted by vegan::adonis function in an ANOVA-like table, as described in documentation [Anderson, 2001; McArdle & Anderson, 2001].<br>Line 171-172: correlation is reported between parentheses when a statement about variance is made. Parentheses containing the correlation metric should be reported before the comma, while variance should be reported at the end of the sentence.<br>AU: The sentence has been clarified. P-values for methane effect are now reported before the comma and R2 indicating the proportion of variance explained is kept at the end of the sentence.<br>Line 179-181: very interesting findings!<br>AU: Thank you!<br>Line 182-183: sentence structure needs revisiting.<br>AU: Changes applied (lines 178-179): “We classified these genera according to their respective overabundance (OA) in the LOW or HIGH emissions groups”.<br>Line 225-226: CLR "helps" but does not avoid compositional artefacts, and therefore it does not avoid spurious correlations. See Quinn et al (2021) "A Critique of Differential Abundance Analysis, and Advocacy for an Alternative" and his previous works<br>AU: Indeed, the word “avoid” was not correct in this sentence, changes have been made to suggest helping instead of total elimination.<br>Line 291-293: sentence structure needs revisiting.<br>AU: Changes applied (lines 332-335): “Pathways related to pathogenic activity were also found, in agreement with the RA of several genera that include known pathogenic species (e.g. Vibrio, Haemophilus, Trypanosoma or Staphylococcus) although not every species from these genera are pathogenic, but opportunistic or commensal organisms”.

Powered by Editorial Manager® and ProduXion Manager® from Aries Systems Corporation

Line 296: As the methods section comes later, SqueezeMeta software needs citation and (possibly) a short intro.
AU: Changes applied, a new section has been added from line 382.
Line 316: sentence structure needs revisiting.
AU: Changes applied (lines 402-403): “Former studies also revealed a link between ruminal microbiota and methane production. Difford et al. showed different clusters of high and low methane emitters within prokaryotic rumen subcompositions”.
Line 369-371 + 396: Good acknowledgement of limitations :)
AU: Thanks again!
Line 378: sentence structure needs revisiting.
AU: Sentence has been revisited (lines 295-296): “Lactate and succinate-producers have been reported to be more abundant in low-emitters as well […]”.
Line 404: VFA acronym has been introduced once 200 lines earlier, could be just spelled out.
AU: Acronym has been removed, as VFA are only mentioned three times.
Line 426-431: Interesting hypotheses!
AU: Thanks again!
Line 484: I am not sure if the data from nanopore sequencing suffers much from the consequences of batch effects, but in other metagenomic sequencing techniques batch effects are unfortunately often cause of trouble. Here batches were used of 12 samples at a time per run. Was the batch effect controlled/tested for? Even though probably the samples were randomised (were they?), a batch effect could still be present. This is worth checking. A batch correction might even improve the signal!
AU: We already controlled batch effect in other experiments using ONT sequencing using this and other datasets. No significant batch effect was detected, nor PCA showed sample discrimination by batches. This effect might be important if sequencing runs experience problems, but our runs have performed appropriately, keeping ONT performance and quality standards, so we did not include batch in our final models.
Line 500: what package was used to assess sparsity?
AU: The details on the packages and methods used for prevalence filtering, i.e., reducing matrix sparsity, can be found in Supplementary Data 2. We did not use any package for sparsity estimation, we counted the density of zeros in the matrix. We also visualized it using zCompositions::zerocount function, but this figures are not included in the paper.
Supplementary Table 1:p>0.05 hits should be included.
AU: We think that this request is not possible to manage, as including p>0.05 hits means to include the entire genera dataset (1,240 genera) for every contrast done (HIGH vs LOW, HIGH vs L-MID, HIGH vs H-MID, L-MID vs H-MID, L-MID vs LOW and H-MID vs LOW). That would become a table of 7,440 genera each repeated 6 times, making it difficult to appreciate the genera significantly DA.

Reviewer #2:
My main point of concern is the suitability of the SqueezeMeta pipeline, and the choice of databases (nr and KEGG) for rumen data.
For example, many groups have now published data showing rumen metagenome genes are very dissimilar to those found in public databases e.g. RefSeq.
The most up-to-date rumen microbiome datasets are:
-the Hungate collection https://www.nature.com/articles/nbt.4110
-Anaerobic fungi from JGI https://mycocosm.jgi.doe.gov/neocallimastigomycetes/neocallimastigomycetes.info.html
-MAG collections (many summarised here: https://www.biorxiv.org/content/10.1101/2021.04.02.438222v1.full.pdf)
-more MAGs and a rumen gene catalogue here: https://microbiomejournal.biomedcentral.com/articles/10.1186/s40168-021-01078-x

As this analysis is the fundamental basis of most of the rest of the manuscript, I need to be reassured it is producing accurate results, and without including the latest rumen genomes and gene catalogues, I am unsure that it is.
AU: SqueezeMeta pipeline is described elsewhere: e.g. https://www.frontiersin.org/articles/10.3389/fmicb.2018.03349/full, https://bmcbioinformatics.biomedcentral.com/articles/10.1186/s12859-020-03703-2. Detailed descriptions can be found in the manual (https://github.com/jtamames/SqueezeMeta/blob/master/SqueezeMeta_manual_v1.4.0.

Powered by Editorial Manager® and ProduXion Manager® from Aries Systems Corporation

pdf). A non-comprehensive list of previous manuscripts using SqueezeMeta can be found at https://github.com/jtamames/SqueezeMeta/wiki/Some-papers-using-SqueezeMeta-(non-comprehensive-list). This software has been tested previously with satisfactory results.
The reference database used is the non-redundant NCBI database, which is the most comprehensive database up to date. Certainly, there may be reads that could align to the MAGs in those data sets, however they can still align at some taxonomical level, and be classified for instances as Unknown Firmicutes. We believe that classifying those reads to a MAG instead to an unknown genus from a certain class does not invalidate our results and there is little added value to the manuscript. We believe that detecting our own MAGs and compare them to these MAGs data bases would be more interesting, and can be tackle in future studies. The resulting microbial composition is similar to previous published results, as we stated from lines 389 to 395.
Nonetheless, we have included the Hungate collection in our database and test the analyses in a couple of samples. The results showed that less than 7% of the reads mapped against KEGGs from such a collection instead to the nrNCBI. Hence, we believe this should not invalidate the results obtained with the reference database used. Indeed, we can benefit from assembling our own MAGs, and combine them with other specific databases as the reviewer suggested. We are working on this objective, but elaborating a more comprehensive database is a different scope from the current study, and we hope that the reviewer understands that it should not preclude the validity of the current study, as we have demonstrated that the reference database used is highly accurate.
We hope that this convinces the reviewer, otherwise the referee needs to specify why he thinks that the bioinformatics analysis is not producing satisfactory results.

Reviewer #3:
This study has generated the largest ONT ruminal metagenomic dataset currently available, and found that ciliates and fungi are closely associated with methane emission. The findings is novel and have wide interest, but the manuscript need revision to be a good manuscript.
1. In all the Figures, the text are not clear. For me, I can't distinguish most of the characters, words, so it must be resolved.
AU: Thank you for your comments. We have considered the carefully. They were really useful and helped improving the manuscript in a nice manner. We would like to point out that the journal's built-in pdf shows the figures in lower quality than the expected, but downloading them is possible to visualize them with high quality. We believe that the format of the figures will be adapted during the editing process keeping the high quality of the figures provided.
2. In the Abstract Part:
"Background: This study analysed whole rumen metagenome using long reads and considering its compositional nature in order to disentangle the role of rumen microbes in methane emissions." Is this really background? I can't see any background and logic.
AU: This is a good point. Thank you. Changes in 'background' were implemented accordingly.
Methods: Line 33-38, can be removed from Abstract. Abstract needs concise, methods is not necessary here.
AU: The methods section from the abstract has been removed, as suggested.
3. In the Results Part:
"3.1 Cohort description: Our cohort included 437 Holstein lactating cows sampled at 14 different herds from northern Spain (Cantabria, Euskadi, Navarra and Girona regions)." This paragraph is very short and only have one sentence. I suggest to put it into methods part, and remove it from the results part. It can be merged with Methods 6.1 "Animal housing and feeding".
AU: Changes made accordingly (lines 434-435).
"3.7 Interaction networks" and "3.8 Taxonomy of genes" do not like sub-title names, they should be revised, to focus on the novel discoveries, which be more meaningful for the readers.
AU: The sub-titles were changed to "Co-abundance of genera and KEGGs" and "Distribution of genes among clades".
4. The Discussion Part:
The biggest problem is that it is too long. I suggest either make them concise, or move some into the results part. Those discussion contents that are closely with the results

	can be merged into the results part, and further discussions can be kept in the Discussion part.<br>AU: We revised the discussion and made some changes, mostly re-organizing parts that were difficult to follow and shortening some paragraphs. We hope that the discussion is more readable now.<br>5. In the Methods Part:<br>"6.6 Bioinformatics" this sub-title is not meaningful, better change it to "Reads filtering, taxonomic and functional assignment" or others.<br>AU: Change applied. New subtitle: "6.5 Read processing, mapping and filtering".<br>6. The last suggestion: try to assemble the ONT long reads, generate gene set, and chromosome results by binning or other technologies. Maybe this will get a lot more useful sequence data.<br>AU: This is a good suggestion for future works, we are currently working in this approach. We think that this is not critical under the current approach of detecting association between ruminal methane production and known microbial gene functions and taxa.
**Additional Information:**	
**Question**	**Response**
Are you submitting this manuscript to a special series or article collection?	No
**Experimental design and statistics**<br><br>Full details of the experimental design and statistical methods used should be given in the Methods section, as detailed in our Minimum Standards Reporting Checklist. Information essential to interpreting the data presented should be made available in the figure legends.<br><br>Have you included all the information requested in your manuscript?	Yes
**Resources**<br><br>A description of all resources used, including antibodies, cell lines, animals and software tools, with enough information to allow them to be uniquely identified, should be included in the Methods section. Authors are strongly encouraged to cite Research Resource Identifiers (RRIDs) for antibodies, model organisms and tools, where possible.<br><br>Have you included the information requested as detailed in our Minimum Standards Reporting Checklist?	Yes

Powered by Editorial Manager® and ProduXion Manager® from Aries Systems Corporation

**Availability of data and materials**<br><br>All datasets and code on which the conclusions of the paper rely must be either included in your submission or deposited in publicly available repositories (where available and ethically appropriate), referencing such data using a unique identifier in the references and in the "Availability of Data and Materials" section of your manuscript.<br><br>Have you have met the above requirement as detailed in our Minimum Standards Reporting Checklist?	Yes

Manuscript with DOI
Click here to access/download;Manuscript;Manuscript_R1 with DOI.docx

Fungal and ciliate protozoa are the main rumen microbes associated with methane emissions in dairy cattle.

Adrián López-García[1], Alejandro Saborío-Montero[1,2], Mónica Gutiérrez-Rivas[1], Raquel Atxaerandio[3], Idoia Goiri[3], Aser García-Rodríguez[3], Jose A. Jiménez-Montero[4], Carmen González[1], Javier Tamames[5], Fernando Puente-Sánchez[5], Magdalena Serrano[1], Rafael Carrasco[6], Cristina Óvilo[1], Oscar González-Recio[1,7*]

[1]Departamento de Mejora Genética Animal. Instituto Nacional de Investigación y Tecnología Agraria y Alimentaria, Crta. de la Coruña km 7.5, 28040 Madrid, Spain.

[2]Escuela de Zootecnia y Centro de Investigación en Nutrición Animal, Universidad de Costa Rica, 11501, San José, Costa Rica

[3]NEIKER – Instituto Vasco de Investigación y Desarrollo Agrario. Basque Research and Technology Alliance (BRTA). Campus Agroalimentario de Arkaute s/n, 01192 Arkaute, Spain.

[4]Confederación de Asociaciones de Frisona Española (CONAFE). Ctra. de Andalucía km 23600 Valdemoro, 28340 Madrid, Spain.

[5]Departamento de Biología de Sistemas, Centro Nacional de Biotecnología, CSIC, Madrid, Spain

[6]Departamento de Periodismo y Nuevos Medios. Universidad Complutense de Madrid, Ciudad Universitaria s/n, 28040 Madrid, Spain.

[7]Departamento de Producción Agraria. Escuela Técnica Superior de Ingeniería Agronómica, Alimentaria y de Biosistemas. Universidad Politécnica de Madrid, Ciudad Universitaria s/n, 28040 Madrid, Spain.

*** Correspondence:**
Óscar González-Recio
gonzalez.oscar@inia.es

Keywords: dairy cattle, microbiome, rumen, methane, Nanopore, long reads.

1 Abstract

Background: Mitigating the effects of global warming has become the main challenge for humanity in the last decades. Livestock farming contributes to greenhouse gas emissions, with an important output of methane from enteric fermentation processes, mostly in ruminants. As ruminal microbiota is directly involved in digestive fermentation processes and methane biosynthesis, understanding the ecological relationships between rumen microorganisms and their active metabolic pathways is essential for reducing emissions. This study analyzed whole rumen metagenome using long reads and considering its compositional nature in order to disentangle the role of rumen microbes in methane emissions.

Results: The beta-diversity analyses suggested an association between methane production and overall microbiota composition ($0.01 < R^2 < 0.02$). Differential abundance analysis identified 36 genera and 279 KEGGs as significantly associated to methane production ($P_{adj}<0.05$). Those genera associated to high methane production were Eukaryota from Alveolata and Fungi clades, while Bacteria were associated to low methane emissions. The genus-level association network showed two clusters grouping Eukaryota and Bacteria, respectively. Regarding microbial gene functions, 41 KEGGs resulted to be differentially abundant between low and high emission animals, and were mainly involved in metabolic pathways. No KEGGs included in the methane metabolism pathway (ko00680) were detected as associated to high methane emissions. The KEGG network showed three clusters grouping KEGGs associated to high emissions, low emissions and not differentially abundant in either of them. A deeper analysis of the differentially abundant KEGGs revealed that genes related with anaerobic respiration through nitrate degradation were more abundant in low emissions animals.

Conclusions: Methane emissions are largely associated to the relative abundance of ciliate and fungi. The role of nitrate electron acceptors can be particularly important as this respiration mechanism directly competes with methanogenesis. Therefore, whole metagenome sequencing is necessary to jointly consider relative abundance of Bacteria, Archaea and Eukaryota in the statistical analyses. Nutritional and genetic strategies to reduce CH_4 emissions should focus on reducing the relative abundance of Alveolata and Fungi in the rumen. This experiment has generated the largest ONT ruminal metagenomic dataset currently available.

2 Introduction

Next generation sequencing technologies have provided special relevance to microbial communities from different niches, as they allow identifying their taxonomic and functional profile. It has made possible to unravel the relationships between host and microbiota, as well as the complex interactions between microbes, with a special contribution to the role of digestive microbiome on complex traits both in humans [1] (e.g. type II diabetes, cancer, mental diseases) and in domestic animals [2,3] (e.g. feed efficiency, methane emissions, animal health).

Microbial communities are of special relevance in livestock. In ruminants, one of the main microbial communities lays in the rumen, due to its high diversity and large microbial mass [4] and its main role in feed fermentation to provide substrate to the animal, which is then transformed into product. Additionally, enteric methane is produced in the rumen by methanogenic microorganisms during feed fermentation [5] and is the main contributor of greenhouse gases (GHG) from livestock, with 2.8 to 3.5 gigatonnes of CO_2-equivalent (CO_2e) per year [6,7]. The ongoing climate emergency urgently calls for efficient strategies to mitigate the carbon footprint from all sectors, including

agriculture and livestock farming. Former studies have proven that complex traits in ruminants are usually influenced by global changes in ruminal microbial communities, more than by fluctuations in the abundance of specific microorganisms [8,9]. These global changes are usually due to the intricate interactions between different species in these communities (*i.e.*, predation, competition of ecological niche or co-dependency). Consequently, a better understanding of the interactions between microbial genes during methanogenesis is needed to propose strategies for reducing methane emissions. Promising strategies have been proposed to modulate the metagenome, nutrition and genetics [10].

Classical statistical approaches do not allow to accurately assess the results of microbiome studies. The high sparsity of these data and their compositional nature generate multiple problems in statistical analysis, including subcompositional incoherence, increase of false positive rates in differential abundance analyses and detection of spurious correlations [11].

As a consequence, new approaches considering both compositionality and multiple correlations are needed. It is also important to point out the advantages of whole metagenome sequencing over metataxonomic studies, because the latter cannot be used to determine functionality and because they pose some difficulties at simultaneously analyzing different superkingdoms [12], which is necessary to account for the total variability of microbiomes and the interactions among their components. Different amplicons must be used to correctly classify *Bacteria*, *Archaea*, *Protozoa* and *Fungi*, increasing the cost of the studies and involving additional bias due to PCR [13]. They pose the additional difficulty of a proper comparison between communities sequenced in different reactions with different primers. Nanopore sequencing offers a cost-efficient sequencing strategy for metagenomics studies providing both taxonomical and functional

information simultaneously and for microbes from all superkingdoms. This technology has been improved in recent years, allowing to perform taxonomic and functional assignments with an accuracy comparable to Illumina [14].

The objective of this study was to characterize the taxonomical and functional composition of rumen microbiota using long sequence reads obtained with Nanopore technology, and their relationship with enteric methane emission.

3 Results

3.1 Taxonomy of microbial composition

After initial selection of core taxonomy, 6,394,671 reads with N50 = 4,022 bp were classified in 3,921 taxonomical features up to genus level. A filtering strategy was implemented to exclude low abundance microbes while keeping the core microbiome relevant for methane emissions. This process removed 48,517 reads (<1%) which reduced the sparsity of the metagenome from 87% to 68%, although a large number of singleton and doubleton features remained (**Supplementary Figure 1**). The final core subcomposition included a total of 6,318,344 reads, in 437 samples, classified in 1,240 taxonomical features: 967 known genera (722 bacteria, 13 archaea and 232 eukaryotes), and 273 that only reached family rank (*i.e.*, *Unclassified* denomination). Overall, 503 families, 277 orders, 158 classes and 86 different phyla (37 bacterial phyla, 3 archaeal phyla and 46 eukaryotic clades) were classified. Relative abundance (RA) distribution by superkingdoms and phyla is summarized below.

Predominant microorganisms in this core rumen subcomposition were bacteria (91.6% ± 6.93 of total average RA) from *Bacteroidetes*, *Firmicutes* and *Fibrobacteres* (**Figure 1**), representing an average RA of 63%, 16% and 5%, respectively. The *Bacteroidetes*

fraction was majorly composed by *Prevotella*, and was the main representative genus in the total community (19.4% average RA), along with other *Prevotellaceae* members. The *Firmicutes* group included a large number of genera. The order of *Clostridiales* dominated in terms of RA, with *Lachnospiraceae* and *Ruminococcaceae* families being the most representative ones. The remaining phyla (34) from the *Bacteria* superkingdom represented 7.6% averaged RA of the core metagenome. Eukaryotes represented a total average RA of 8.2% (±6.95) of the core subcomposition. Predominant eukaryotic clades were those included in the *SAR* supergroup (*Stramenopiles-Alveolata-Rhizaria*) [15], accounting for 6% of total average RA, followed by *Fungi* (1.3% of total average RA). *Alveolata* clade was the most abundant among the eukaryotes, with a high representation of unclassified *Ophryoscolecidae*, *Stentor* and *Paramecium*. *Archaea* representation in the core subcomposition (0.24% ± 0.25 of total average RA) consisted mostly of *Methanomicrobia*, *Methanobacteria* and *Thermoplasmata* members. Yet, a large number of reads could not be assigned to a known genus. The relative abundance per animal of the most relevant taxonomic groups is depicted in **Supplementary Figure 2**.

3.2 Functionality of microbial composition

A total of 30,326,550 reads with N50 = 5,720 bp were assigned to KEGGs. After prevalence filtering, a total of 84,219 reads (0.28%) were removed and the sparsity was reduced from 72% to 39% (**Supplementary Figure 1**). The final KEGG table was composed by 30,145,459 reads from 437 samples, classified in 6,644 KEGGs. These KEGG pathways and BRITE hierarchies [16–18] were represented in a Treemap according to their average RA (**Figure 2**). A 26% of the rumen metagenome functions were in pathways that represent the metabolism of carbohydrate, amino acid and other biological compounds, as well as of energy metabolism. In addition, 11% of functions were involved in cellular generic processes (cell growth (3%), transport and catabolism

(4%), or genetic and environmental information processing (23%)). KEGG BRITE classification showed a high presence of proteins involved in cellular processes (36%) and metabolism (26%).

3.3 Beta-diversity and PERMANOVA analysis

Beta-diversity was represented in Principal Component Analysis (PCA) between samples at five different taxonomic levels (phylum, class, order, family and genus), as well as with KEGG, using centered log-ratio (CLR) transformed datasets. Then a permutational analysis of variance (PERMANOVA) was implemented [11], sequentially adding the effect of farm-batch (B), stage of lactation (SL), number of lactation (NL) and level of methane emissions (CH4) discretized in four groups (LOW, L-MID, H-MID and HIGH). The visualization did not show a clear visual clustering of samples by methane emission levels (**Figure 3**). However, a generalized additive model (GAM) smooth fitting allowed visualizing non-linear distribution patterns of the microbial samples according to CH_4 emissions inside the ordination at all taxonomic levels. The non-linear pattern was more evident at the phylum, class and genus levels, although the proportion of methane variability explained was low (≃4.8% according to GAM model fitting). No relevant differences were visually appreciated using the KEGG information. Nonetheless, some differences in the overall rumen microbiome composition between animals with different methane emissions were evidenced by the PERMANOVA analysis, both for taxonomy and functionality (Table 1). The results showed significant differences for the centroid distance between methane emission groups at every taxonomic level and also for KEGGs ($P < 0.01$), but they explained a low percentage of total variance ($0.01 < R^2 < 0.02$).

3.4 Rumen microbes associated to CH_4 emissions

The effect of taxonomical features on methane emission levels was evaluated through differential abundance analysis. Thirty-three genera were found as differentially abundant (DA) (P_{adj} <0.05) between LOW and HIGH emitters (**Figure 4A**), while 15 genera showed DA between LOW and H-MID emitters and one genus between LOW and L-MID emitters (**Supplementary Data 1**). Note that 13 out of the 15 genera showing DA (P_{adj} <0.05) between LOW and H-MID groups were also significant in the LOW vs HIGH contrast, but not in LOW vs L-MID contrast, indicating gradual abundance change from low to high emitters. Accounting for all contrasts and duplicated genera, 36 DA genera resulted significant. We classified these genera according to their respective overabundance (OA) in the LOW or HIGH emissions groups. Thus, 10 of them were more abundant in the LOW group (LOW-OA) and 1 in the L-MID group. The remaining 25 genera were OA in the HIGH groups (HIGH-OA): HIGH (12), HIGH and H-MID (11) or H-MID (2). HIGH-OA genera represented an overall RA of 4.15%, whereas LOW-OA genera accounted for 0.25% of total RA. The two genera over-abundant in H-MID were *Dictyostelium* and *Unclassified Eimeriidae*, and the one associated to L-MID was classified as *Candidatus Izimaplasma* (*Tenericutes*). The log_2FC values ranged between 0.7 and -0.7 in genera showing DA for methane emission levels, highlighting that the differences between groups were moderate.

Overall, DA results indicate that taxa associated to higher methane levels belong to the *Eukaryota* superkingdom, while those associated to lower emissions were bacteria. We found multiple *Ciliophora* genera associated to the HIGH group (mostly *Parameciidae*, *Stentoridae* and *Pseudocohnilembidae* members) but also *Amoebozoa* and some *Fungi* or pseudo-fungi. Other bacterial genera associated to lower methane production were *Hespellia*, from *Clostridiales*, and *Sutterella*, an asaccharolytic genus from *Betaproteobacteria*.

3.5 Microbial gene function associated to CH_4 emissions

Differential abundance analysis was also performed for KEGG features on methane emission levels. A total of 192 KEGGs were DA between the LOW and HIGH emissions groups (**Figure 4B**). Differences were also found between the LOW and H-MID groups (**Supplementary Data 1**). As in the taxonomy dataset, some of the KEGGs presented significant DA in both LOW vs HIGH and LOW vs H-MID contrasts. Accounting for these duplicates and all the contrasts, 182 were over-abundant in the high emissions groups (HIGH-OA), whereas 97 KEGGs were over-abundant in low emissions groups (LOW-OA). The overall RA for HIGH-OA KEGGs was 2.31% and 0.64% for LOW-OA KEGGs. Of these, 13 HIGH-OA KEGGs and 28 LOW-OA KEGGs were assigned to metabolic pathways. No KEGGs from the ko00680 pathway were found as HIGH-OA. KEGGs related to inositol-phosphate metabolism (K00889, K01110, K18082 and K20279), starch and sucrose metabolism (K01203) or several lipid metabolism pathways were present in the HIGH-OA group. According to LOW-OA KEGGs, some of them were involved in volatile fatty acid metabolism (e.g., K00209 enoyl-[acyl-carrier protein] reductase [EC:1.3.1.9], K01902 succinyl-CoA synthetase alpha subunit [EC:6.2.1.5] and K01682 aconitate hydratase 2 [EC:4.2.1.3]) and the K09251 putrescine aminotransferase [EC:2.6.1.82] related to putrescine and cadaverine degradation to 4-amino-butanoate (GABA) or 2-oxoglutarate. Also, several KEGGs in the LOW-OA group were related to N metabolism (K00370 and K00371 nitrate reductase subunits [EC:1.7.5.1]), oxidative phosphorylation (K03885 NADH dehydrogenase [EC:1.6.99.3]) and to carbohydrate, lipid or vitamin metabolism pathways. The ko00680 KEGG K13788 was also over-abundant in the LOW emissions group.

3.6 Co-abundance of genera and KEGGs

Interaction networks were built using the previous results in order to visualize the association between taxa and genes using pairwise correlations between features. Pairwise proportionality correlation coefficients (ρ_p) were calculated on the CLR-transformed datasets for phylum, genus and KEGG features to mitigate the effect of spurious correlations that can potentially surge in compositional data [19].

The most relevant pairwise proportionalities between genera and between KEGGs were visualized as interaction networks, classifying features as associated to high methane emissions (HIGH), low methane emissions (LOW) or not associated to methane emissions (N/A), according to the results from the differential abundance analyses. The interaction networks for genera and KEGGs are shown in **Figure 5** and **Figure 6**, respectively.

Eukaryotes clustered together in the network with large representation of the *SAR* supergroup, and showed negative proportionality to bacteria. The genera that were associated to higher methane emissions belonged to the *Eukaryota* superkingdom (*Ciliophora* and *Fungi*), whereas *Bacteria* were associated to lower CH_4 production. The strongest inverse proportionalities between both subpopulations connected several eukaryotes with *Unclassified Veillonellaceae* and *Oribacterium* ($-0.64 < \rho_p < -0.53$), *i.e.*, microbiomes with lower abundance of *Oribacterium* or *Veillonellaceae* tend to present larger abundances of protozoa and fungi, and were therefore associated to larger emissions. Unclassified microbes from *Neocallimastigaceae*, *Oxytrichidae* and *Vibrionaceae* families showed the highest centrality and a large connectivity degree.

The functional network showed three main clusters that grouped KEGGs associated to HIGH methane level (cluster H), KEGGs not related to methane emissions (cluster N), and a small one including KEGGs associated to lower emissions (cluster L). Connections

between clusters were not symmetric: H cluster was connected to N cluster by inverse proportionalities between some of their components, but the L cluster appeared connected only to N cluster by direct proportionalities through non-clustered KEGGs. Also, most of the ko00680 KEGGs (*i.e.*, directly involved in methanogenesis or participating in pathways leading to methanogenesis precursors) did not appear as differentially abundant between high-emission and low-emission cows.

3.7 Distribution of genes among clades

A traceback of genes' taxonomy was carried out, separately for ko00680 KEGGs and for DA KEGGs. Thirty out of the 85 ko00680 KEGGs were predominant in Archaea groups, one predominated in *Eukaryota* (K05979) and the rest were predominant in *Bacteria* (**Figure 7**). Although the RA distribution of these KEGGs was normally between 60% and 100% in the predominant superkingdom, 4 KEGGs were more evenly distributed between clades: K01007 and K00863 had a RA < 60% in *Bacteria* and showed RA > 30% in *Eukaryota*; K05979 was the KEGG predominating in *Eukaryota*, but with a RA near to 60% (38% in *Bacteria* and 12% in *Archaea*); and K14080 had a RA of 57% in *Archaea* and 43% in *Bacteria*. Regarding the DA KEGGs, those from the LOW-OA group showed larger abundance in *Bacteria*, mostly in genera from *Proteobacteria*, *Bacteroidetes* and *Firmicutes* phyla. Different groups of bacteria also carried KEGGs from the HIGH-OA group although these KEGGs were more abundant in eukaryotes. The HIGH-OA KEGGs were mainly mapped to unclassified eukaryotes, but those which could be classified belonged majorly to *Fungi* and *SAR* supergroup (**Figure 8**).

4 Discussion

In this study we assessed the composition of the ruminal microbiota using long reads from Nanopore sequencing technology. We observed predominance of *Bacteroidetes*,

Firmicutes and *Fibrobacteres*, as reported in previous studies [8,20]. *Bacteroidetes* and *Firmicutes* are common bacteria in all kind of ecosystems, including gut microbiota of multiple animals. The fraction of *Bacteroidetes* was mainly composed by *Prevotella*, a group of anaerobic gram-negative bacteria involved in saccharolytic processes [21]. Their large abundance in the digestive microbiota has been previously reported in ruminant [22–26] and monogastric species [27,28]. *Firmicutes* were less abundant, with a more diverse distribution of genera. *Fibrobacteres*, a small group of cellulose-degrading bacteria usually present in ruminant digestive system [29], was mainly represented by the *Fibrobacter* genus. Eukaryotes also represented a relevant amount of the rumen core metagenome. This group has been reported to contribute up to 50% of total ruminal biomass [30]. The *SAR* supergroup and *Fungi* were the most relevant ones, which are found in a wide variety of ruminants and pseudoruminants [15,31]. Other eukaryotes included *Stentor* and *Paramecium*, the former are aquatic free-living heterotricheans which can be particle filtrators or predators of other protozoa and live symbiotically with some algae species [32,33], whereas the latter are well-known ciliates which predate bacteria and other microorganisms, including protozoa [34]. Archaeal fraction was mostly composed by strict methanogenic organisms from *Methanomicrobia* and *Methanobacteria* clades [35], but also included *Thermoplasmata*, which are methylotrophic-methanogenic acidophilic organisms [36].

The DA analysis showed that ciliates, fungi and pseudo-fungi were more abundant in cows with higher levels of methane emissions. Microbes associated to lower methane emissions were saccharolytic members of class *Gammaproteobacteria* (*Anaerobiospirillum* [37], *Vibrio* [38] or *Pseudoalteromonas* [39]), as well as *Negativicutes* genera from *Veillonellaceae* (*Dialister*, *Megasphaera*) and *Selenomonadaceae* (*Mitsuokella*). *Dialister* produce succinate decarboxylation, and

Megasphaera ferment carbohydrate and lactate [40], while *Mitsuokella* are saccharolytic bacteria [41]. The low-emissions ruminotype had larger abundance of *Proteobacteria* and *Firmicutes* genera. Other authors also reported higher abundances of these bacterial phyla in low methane emissions animals [8]. Lactate and succinate-producers have been reported to be more abundant in low-emitters as well [42], supporting the higher abundance of *Anaerobiospirillum* or *Megasphaera* in LOW animals.

Despite this association between methane and large taxonomic groups, it is of interest to find out which specific clades and microbial genes are participating directly or indirectly in methanogenesis. The genera co-abundance network showed a clear cluster of eukaryotes, with many of them being significantly more abundant in the high emissions group. Other authors have already established a positive correlation between fungi abundance and methane emissions [8], as well as a close interdependence of protists and fungi. Although correlation between methane emissions and protozoa abundances is still under discussion [43,44], current meta-analyses point to a linear relationship between protozoa abundance and methane emissions (r=0.96) [45].

Interestingly, no taxonomic group of methanogenic archaea showed association with methane emissions. The relationship between Archaea and methane production in rumen is not consistent in the literature. Some authors reported either individual relationships between methane emissions and some archaeal species [46,47] or correlations between overall archaeal gene abundance and methane emissions level [43,48]. However, other studies showed no relationship between methanogenic *Archaea* and methane [47,49]. All studies to date showed a low relative abundance of archaea in the rumen, compared to eukaryotes and bacteria [50]. However, the association between the abundance of rumen eukaryotes and methane emissions has been demonstrated through defaunation experiments, both in vitro [51,52] and in vivo [44,53], with lower emissions in defaunated

animals [54]. This has been attributed to the tight link existing between methanogenic archaea abundance and some fungi and protozoa [50]. Specifically, ciliates and some *Chytridiomycota* (e.g. *Neocallimastix* sp.) are known to symbiotically engulf a variety of methanogenic archaea. They provide the archaea with substrate for methane production from H_2 produced in their hydrogenosomes, as well as protection against oxygen toxicity [30,55,56]. Thus, free-living methanogens might represent a low fraction of microbial population [45], and CH_4 biosynthesis might be more influenced by endosymbiotic methanogens [55]. Hence, a larger methanogenesis activity is expected to be correlated with a larger abundance of eukaryotes, especially ciliates, which are more abundant and better represented. Another partial explanation for the low abundance of free archaea, and thereby for the lack of association between Archaea and methane emissions in previous studies [10], is that lysis of archaea cell walls often requires specific protocols during DNA extraction, and they might be under-represented in metagenomics studies [57].

In terms of Gene Ontology, the KEGGs were associated to several metabolic functions and cellular processes (nutrient metabolism and biosynthesis, cellular transport, cell growth or genetic information processing). Pathways related to pathogenic activity were also found, in agreement with the RA of several genera that include known pathogenic species (e.g. *Vibrio*, *Haemophilus*, *Trypanosoma* or *Staphylococcus*) although not every species from these genera are pathogenic, but opportunistic or commensal organisms. Besides, pathogenic activity presence in our dataset might be biased due to a larger representation of human related diseases in the databases. The KEGGs were classified according to their presence or absence in ko00680 pathway (methane metabolism), as a way to evaluate their direct involvement in methanogenesis or an indirect involvement in pathways leading to biosynthesis of precursor compounds. Although we found several ko00680 KEGGs which are presumably involved in the biosynthesis of methanogenesis

precursors, most of them were not associated to methane emissions (*i.e.*, not differentially abundant between methane groups). Most of these KEGGs were mainly present in bacteria or eukaryotes and might be functioning in metabolic pathways not related to methanogenesis. For instance, some of the KEGGs inside the methane metabolism pathway can also be involved in glycine, serine and threonine metabolism (e.g. K00058, K00831, K01079 and K00600), pyruvate and propanoate metabolism (e.g. K00625 and K13788), glycolysis (e.g. K01689, K15633, K01624 and K02446) or anaerobic carbon fixation (e.g. K00198) [16–18]. Another group of ko00680 KEGGs is exclusive from *Archaea*, but the under-representation of this clade in our dataset might obscure statistical significance.

Other detected KEGGs could be indirectly related with methanogenesis through biosynthesis of precursor compounds. For instances, K00209 and K13788 are involved in butyrate and propanoate biosynthesis, being essentially carried by primary fermentative bacteria [58]. Then the volatile fatty acids can be used by secondary fermenters to produce methanogenesis precursors such as H_2, CO_2, acetate and formate [59,60]. In fact, K13788 is a phosphate acetyltransferase [EC:2.3.1.8] that can be involved in the biosynthesis of acetate from acetyl-CoA [61]. Also, K09251 is involved in biosynthesis of GABA and 2-oxoglutarate. GABA has been related with a volatile fatty acid concentration increment [62], while 2-oxoacid compounds can be used by *Archaea* to synthesize coenzyme M and coenzyme B, which are essential in methane production [63]. However, all these KEGGs were observed as over-abundant in LOW methane group, suggesting a strong presence of fermentative bacteria in these animals, not directly correlated with methane production.

Other KEGGs that were over-abundant in LOW emitters might offer an explanation to the lower presence of active methanogenesis processes through competence mechanisms

(e.g. LOW-OA KEGGs K01682, K01902 and K13788, are involved in citrate cycle and pyruvate metabolism, related to respiration). The K00370 and K00371 are nitrate oxidoreductase subunits playing a role in anaerobic respiration using nitrate as electron acceptor. This enzyme uses nitrate as electron acceptor, a process that has been reported as competitive inhibitor of methanogenesis [64,65]. Nitrate supplementation has proven to be an useful strategy to mitigate methane emissions [66]. Nitrite produced by the nitrate-reductases has a known antimicrobial effect and toxicity to animal cells [67–69], which might also reduce the proportion of free archaea in LOW animals, although toxicity to archaea must be further studied [70]. However, the role of ciliates and fungi must be clarified, as their abundance is also lower in LOW emitters. We hypothesize that the predatory nature of these eukaryotes might be a control mechanism for bacterial populations, and their lower relative abundance in LOW animals might allow overgrowth of related bacteria. Nevertheless, there is the possibility that a higher proportion of facultative anaerobes using nitrate as acceptor might affect ciliate populations by toxicity, thus reducing the presence of endosymbiotic methanogenic archaea.

The SqueezeMeta software [71] uses a last common ancestor (LCA) algorithm, which assigns to one read the lowest-level taxon common to all hits, using a stringent cutoff identity value for each taxonomic rank. On its part, functional assignments are done with fun3 algorithm, which by default assigns the hit with the highest average bitscore compared to the n first hits passing the e-value, identity and coverage filters. This LCA approach ensures that reads have a large probability of being correctly classified, at expense of a large number of reads remaining unclassified, which explains the larger number of reads assigned to a known KEGG than to taxa. Despite this strict requirement, this composition is consistent with other populations reported before [2,3,20]. Most studies to date report large abundance of *Bacteroidetes* and *Firmicutes*, with *Prevotella*

spp. as the most prevalent genus. Some minor discrepancies with other studies were observed in the RA of the core subcomposition. For example, Wallace *et al.* [20] showed a higher presence of *Proteobacteria* and *Euryarchaeota*, although using amplicons instead of whole metagenome sequencing.

Our statistical approach evidenced the difficulty of inferring a phenotypic association between microbiome composition and methane production, with an important role of environmental factors that mask the statistical signal. However, a meaningful relationship between the microbiome composition and methane emissions could be uncovered yet, emphasizing the role of the different phyla, with the *Eukaryota* superkingdom being of particular relevance. Former studies also revealed a link between ruminal microbiota and methane production. Difford *et al.* [3] showed different clusters of high and low methane emitters according to their bacterial and archaeal subcomposition. Danielsson *et al.* [46] also found clustering for low and high methane emitters within prokaryotic rumen subcompositions. Wallace *et al.* [20] found that a core set of rumen microbiome was capable of explaining up to 30% of methane emissions variability, mostly formed by prokaryotes. The aforementioned studies used different methodologies, like amplicon analysis and OTU clustering, contrasting with our full-metagenome genus-clustering protocol, which increases the information entropy. Stewart *et al.* [72] used Nanopore sequencing and found significant differences between low and high-methane emitter sheep, with clear clustering between groups, but using a lower number of microbial groups and animals in the same farm with similar management practices.

5 Conclusions

The full metagenome compositional analysis used in this study provided novel insights in the association between the microbiota and CH_4 emissions through differential

abundance analysis, pairwise correlation and interaction networks. Our approach evidenced a phenotypic association between microbiome composition and methane production, regardless of the challenges posed by the microbiome complexity and the compositional nature of the data. This association is mainly driven by the relative abundance of ciliates and fungi, which carry host specific genetic functions providing substrate to the methanogenic archaea. On the other side, we detected some bacterial groups that performed a more efficient feed digestion, leaving less hydrogen available to archaea and hence associated to lower methane emissions.

This study generated the largest ruminal metagenomic dataset sequenced using ONT and grants free access to a publicly-available data set. The complexity of the rumen microbiome and the compositional nature of their sequencing data require proper statistical methods to allow disentangling the role of microbes and their genes in host complex traits such as methane emissions. Future nutritional and genetic strategies to reduce CH_4 emissions should focus on reducing the relative abundance of *Alveolata* and *Fungi* in the rumen, without impairing other important metabolic processes for an efficient feed digestion in ruminants.

6 Methods

6.1 Animal housing and feeding

Our cohort included 437 Holstein lactating cows sampled at 14 different herds from northern Spain (Cantabria, Euskadi, Navarra and Girona regions). The animals received total mixed ration (TMR) diet differently formulated on each individual herd, although most of them were based on maize and grass silage plus concentrate. Cows were fed ad-libitum, with concentrate supplementation in the automatic milking station (AMS) during milking.

6.2 Methane measuring

Methane concentration was individually recorded through breath sampling during each cow visit to the AMS (3-7 times daily) in a period of 2-3 weeks. Eructation peaks were recorded using a non-dispersive infrared methane detector (Guardian NG infrared gas monitor, Edinburgh Sensors, Scotland, UK) as described by Rey *et al.* (2019) [73]. Each cow's peaks were then averaged in order to get a unique methane record per cow, as described in López-Paredes *et al.* (2020) [74]. Animals were distributed in groups according to number of lactation (NL) and stage of lactation (SL) criteria. Furthermore, quartile-based qualitative categories were created for CH_4 recordings (ppm), resulting in a methane factor (CH4) with 4 levels (LOW, L-MID, H-MID and HIGH methane emissions).

6.3 Ruminal content sampling

Ruminal fluid was sampled using an oral tube (18 mm diameter and 160 mm long) connected to a 1000 mL Erlenmeyer flask and continued to a mechanical pump (Vacubrand ME 2SI, Wertheim, Germany), with all the material contacting the cow being carefully cleaned between cows. Each animal was moved to an individual stall for this process. The solid fraction of the ruminal content was discarded by filtering through 4 layers of sterile cheesecloth, while the outcoming liquid fraction was instantly frozen using liquid nitrogen (LN_2) and then stored at -80 °C until DNA extraction.

6.4 DNA extraction and sequencing

Genomic DNA was extracted from 250 µl of each thawed and homogenized ruminal content sample, using the "DNeasy Power Soil" commercial kit (QIAGEN, Valencia, CA, USA). Qubit fluorometer (ThermoFisher Scientific, 150 Waltham, MA, USA) and

Nanodrop ND-1000 UV/Vis spectrophotometer (Nanodrop Technologies Inc., DE, USA) were used to measure DNA concentration and purity. 260/280 and 260/230 ratios were around 1.8 and 2.0, respectively. Oxford Nanopore Technologies (ONT) SQK-LSK109 Ligation Sequencing kit was used for multiplexed sequencing in MinION automatic sequencer. The 1D Native barcoding ONT kit (EXP-NBD104 or EXP-NBD114) was used for multiplexing the samples, pooling barcoded DNA from 12 samples for each run. Pooling was done using a 1.5 ml DNA LoBind tube to perform adapter ligation and sequenced using a R9.4.1 flow cell.

6.5 Read processing, mapping and filtering

Guppy toolkit (ONT) was used for basecalling. A quality control was then applied removing sequences with QS<7 and length<150 bp. Sequence analysis was performed using SqueezeMeta (SQM) pipeline for long reads [71], which performs Diamond Blastx against GenBank nr taxonomic database and against COG and KEGG functional databases, then identifying and annotating ORFs using the LCA method for taxonomy and the fun3 algorithm for functional annotation (based on e-value and identity scores). This tool is specifically designed to process long reads from ONT.

49,718,901 reads were processed in Blastx by SQM longreads pipeline. Blastx mapped 25,750,755 reads (51.79%) to taxonomy (NCBI-nr database) or function (KEGG database). All sequences mapped as non-microbial (*i.e.*, virus, animals and plants) were discarded. Microbial sequences were then filtered by prevalence to reduce data sparsity and sequencing errors (**Supplementary Data 2**). A first estimation of sample sparsity and reads distribution was assessed using R. Two animals were then withdrawn from the filtered dataset, one due to low read coverage and other due to lack of host information, leaving 437 animals in the final dataset.

Genera were divided into superkingdom groups (Archaea, Bacteria or Eukaryota) and KEGGs were sorted by their involvement in methane metabolism (MP): KEGGs included in the KEGG orthology pathway ko00680 (Methane metabolism) were labeled as “ko00680”, while the rest were identified as “Other”.

6.6 Compositional data

Considering the compositional nature of metagenomic data, a CLR method [75] was applied using the unweighted option of the *CLR* function from the *easyCODA* R package [76] as follows:

$$\mathbf{x_{clr}} = [log(x_1/G(x)), log(x_2/G(x)) \dots log(x_D/G(x))],$$

with $G(x) = \sqrt[D]{x_1 * x_2 * \dots * x_D}$.

Being $\mathbf{x} = [x_1, x_2, \dots, x_D]$ a vector of counted features (taxa or KEGGs) in one sample and *G(x)* the geometric mean of **x**. Count zero values in the initial data frame were imputed through the Geometric Bayesian Multiplicative (GBM) procedure, using the *zCompositions* R package [77] *cmultRepl* function, so that logarithms could be computed.

6.7 Beta-diversity and PERMANOVA analysis

The CLR-transformed data (at phylum, class, order, family, genus and KEGG levels) were used to explore beta-diversity in the samples through PCA using the *prcomp* function in R. Fitted smooth surface of methane emissions corrected by SL and NL was included for principal components 1 and 2 using *ordisurf* function from the vegan R package [78]. A generalized additive model smooth fitting (GAM) was used in order to elucidate non-linear distribution of samples in PCA according to methane emissions. Differences between centroid distances using methane as grouping variable (CH4) were

determined through Permutational Multivariate Analysis of Variance (PERMANOVA) [79,80] following this model and using the matrix of Aitchison distances between samples (*i.e.*, the Euclidean distance on CLR-transformed data) as input variable:

$$D_{jklni} = \mu + B_j + SL_k + NL_l + CH4_n + e_{jklni}$$

with B_j being the farm-batch effect (j = 24 levels), SL_k being the stage of lactation at the day of sampling (k = 3 levels), NL_l the number of lactation (l = 2 levels) and $CH4_n$ the methane emission level (n = 4 levels: LOW, L-MID, H-MID, HIGH), and e_{jklni} was the corresponding residual term.

6.8 Association between microbiota and methane production

Differential abundance of genera and KEGGs between samples regarding the different methane emissions levels was addressed through linear regression using Limma [81]. Count normalization and log-transformation were addressed using CLR-transformed data as inputs. P-values were adjusted by Benjamini-Hochberg method, to control false discovery rate. Differential abundance threshold was set to $|\ \log_2 FC\ | \geq 0.5$ and the adjusted significance threshold was set to $\alpha = 0.05$.

6.9 Pairwise proportionality analysis

Pairwise correlations between phyla, genera and KEGGs were calculated as described in the *propr* R package [82]. Proportionality coefficient ρ_p [83] under CLR data transformation was chosen. Thresholds were selected according to two conditions: 1) representing the maximum number of proportionalities avoiding computational issues; 2) FDR lower than 1%. Used threshold were $|\rho_p| \geq 0.4$ for genera proportionalities and $|\rho_p| \geq 0.7$ for KEGG proportionalities.

6.10 Microbial networks

Microbial networks for taxonomy (at the genus level) and functionality were built from the proportionality matrices described above. Input edges were defined from the cytoscape function in *propr* package in R, which converts a propr object into a data frame of node connections compatible with Cytoscape software (v. 3.8.0). Results from the DA analyses were used to associate each feature (node) to high or low methane emissions levels. Significantly over-abundant genera and KEGGs in the low methane emitters group (*i.e.*, more abundant in LOW than in HIGH or H-MID groups) were designated as LOW-associated, while those contrary over-abundant in high methane emitters were appointed as HIGH-associated. Non-DA features were classified as N/A (not associated). In addition, SK and MP factors were included as node attributes for genera and KEGGs, respectively. For graph visualization, Kamada-Kawai algorithm (Edge-weighted spring embedded layout) was set [84], using ρ_p coefficient as force parameter.

7 Ethical statement

This study was conducted in accordance with Spanish Royal Decree 53/2013 for the protection of animals used for experimental and other scientific purposes and was approved by the Basque Institute for Agricultural Research and Development Ethics Committee (Neiker-OEBA-2017-004) on March 28, 2017.

8 Conflict of Interest

The authors have not stated any conflicts of interest.

9 Author Contributions

A.L.G. and A.S.M. filtered and prepared the data, implemented the statistical analyses and prepared the first draft of the manuscript. M.G.R and C.G. performed the DNA extraction and sequencing. O.G.R. supervised the DNA sequencing and contributed to the statistical analyses. R.C. contributed to develop interaction networks. O.G.R., A.G.R, R.A., I.G. conceived the study and designed the experiments. J.T. and F.P.S developed the computational pipelines for the metagenome and assisted on its analyses. A.L.G., A.S.M. and O.G.R. wrote the manuscript. All authors helped writing and configuring the last version of the manuscript.

10 Funding

This research was financed by RTA2015-00022-C03-02 (METALGEN) and RTI2018-096487-RC33 projects from the national plan of research, development and innovation 2013-2020 and the Department of Economic Development and Competitiveness (Madrid, Spain).

11 Acknowledgments

We thank the regional Holstein Associations and farmers collaborating in the project. Computational support from the High-Performance Computing Centre in Galicia (Spain) is acknowledged. A.L.G. was funded by FPI-INIA grant with reference FPI-SGIT2016-06.

12 Consent for publication

Not applicable.

13 Availability of supporting data and material

The datasets generated during and/or analyzed during the current study have been uploaded to ENA browser, with accession number of the project: PRJEB44278 (https://www.ebi.ac.uk/ena/browser/view/PRJEB44278) and can be requested to the METALGEN project, https://www.metalgen.es.

SqueezeMeta software is available at https://github.com/jtamames/SqueezeMeta. Guppy basecaller software was used to convert fast5 raw signals to fastq files (https://community.nanoporetech.com/downloads). The R environment and used packages are available from https://cran.r-project.org/. Correspondence and material requests should be addressed to Oscar González-Recio (gonzalez.oscar@inia.es). Other data further supporting this work are openly available in the GigaScience repository, GigaDB [85].

14 References

1. Wang B, Yao M, Lv L, Ling Z, Li L. The Human Microbiota in Health and Disease. *Engineering*. 2017; doi: 10.1016/J.ENG.2017.01.008.

2. Delgado B, Bach A, Guasch I, González C, Elcoso G, Pryce JE, et al.. Whole rumen metagenome sequencing allows classifying and predicting feed efficiency and intake levels in cattle. *Sci Rep*. 2019; doi: 10.1038/s41598-018-36673-w.

3. Difford GF, Plichta DR, Løvendahl P, Lassen J, Noel SJ, Højberg O, et al.. Host genetics and the rumen microbiome jointly associate with methane emissions in dairy cows. Leeb T, editor. *PLoS Genet*. 2018; doi: 10.1371/journal.pgen.1007580.

4. Weimer PJ. Redundancy, resilience, and host specificity of the ruminal microbiota: Implications for engineering improved ruminal fermentations. *Front Microbiol*. 2015; doi: 10.3389/fmicb.2015.00296.

5. Knapp JR, Laur GL, Vadas PA, Weiss WP, Tricarico JM. Invited review: Enteric methane in dairy cattle production: Quantifying the opportunities and impact of reducing emissions. *J Dairy Sci*. 2014; doi: 10.3168/JDS.2013-7234.

6. Gerber, PJ, Steinfeld, Henderson, Mottet, Opio, et al.. Tackling Climate Change Through Livestock. A global assessment of emissions and mitigation opportunities. Rome, Italy: Food and Agriculture Organization of the United Nations (FAO); 2013; doi: www.fao.org/publications.

7. FAO: Global Livestock Environmental Assessment Model (GLEAM). Food Agric. Organ. http://www.fao.org/gleam/results/en/ (2019). Accessed 2021 Aug 4.

8. Martínez-Álvaro M, Auffret MD, Stewart RD, Dewhurst RJ, Duthie CA, Rooke JA, et al.. Identification of Complex Rumen Microbiome Interaction Within Diverse Functional Niches as Mechanisms Affecting the Variation of Methane Emissions in Bovine. *Front Microbiol*. 2020; doi: 10.3389/fmicb.2020.00659.

9. Malmuthuge N, Guan LL. Understanding host-microbial interactions in rumen: Searching the best opportunity for microbiota manipulation. *J Anim Sci Biotechnol*. 2017; doi: 10.1186/s40104-016-0135-3.

10. Negussie E, de Haas Y, Dehareng F, Dewhurst RJ, Dijkstra J, Gengler N, et al.. Invited review: Large-scale indirect measurements for enteric methane emissions in dairy cattle: A review of proxies and their potential for use in management and breeding decisions. *J Dairy Sci*. 2017; doi: 10.3168/jds.2016-12030.

11. Gloor GB, Macklaim JM, Pawlowsky-Glahn V, Egozcue JJ. Microbiome datasets are compositional: And this is not optional. *Front Microbiol*. 2017; doi: 10.3389/fmicb.2017.02224.

12. Liu YX, Qin Y, Chen T, Lu M, Qian X, Guo X, et al.. A practical guide to amplicon and metagenomic analysis of microbiome data. *Protein Cell*. 2021; doi: 10.1007/s13238-020-00724-8.

13. Bonk F, Popp D, Harms H, Centler F. PCR-based quantification of taxa-specific abundances in microbial communities: Quantifying and avoiding common pitfalls. *J Microbiol Methods*. 2018; doi: 10.1016/J.MIMET.2018.09.015.

14. Brandt C, Bongcam-Rudloff E, Müller B. Abundance tracking by long-read nanopore sequencing of complex microbial communities in samples from 20 different biogas/wastewater plants. *Appl Sci*. 2020; doi: 10.3390/app10217518.

15. Burki F, Shalchian-Tabrizi K, Minge M, Skjæveland Å, Nikolaev SI, Jakobsen KS, et al.. Phylogenomics reshuffles the eukaryotic supergroups. Butler G, editor. *PLoS One*. 2007; doi: 10.1371/journal.pone.0000790.

16. Kanehisa M, Goto S. KEGG: Kyoto Encyclopedia of Genes and Genomes. *Nucleic Acids Res*. 2000; doi: 10.1093/nar/28.1.27.

17. Kanehisa M. Toward understanding the origin and evolution of cellular organisms. *Protein Sci*. 2019; doi: 10.1002/pro.3715.

18. Kanehisa M, Sato Y, Furumichi M, Morishima K, Tanabe M. New approach for understanding genome variations in KEGG. *Nucleic Acids Res*. 2019; doi: 10.1093/nar/gky962.

19. Lovell D, Pawlowsky-Glahn V, Egozcue JJ, Marguerat S, Bähler J. Proportionality: A Valid Alternative to Correlation for Relative Data. *PLOS Comput Biol*. 2015; doi: 10.1371/journal.pcbi.1004075.

20. Wallace RJ, Sasson G, Garnsworthy PC, Tapio I, Gregson E, Bani P, et al.. A heritable subset of the core rumen microbiome dictates dairy cow productivity and emissions. *Sci Adv*. 2019; doi: 10.1126/sciadv.aav8391.

21. Shah HN, Collins DM. Prevotella, a new genus to include Bacteroides melaninogenicus and related species formerly classified in the genus Bacteroides. *Int J Syst Bacteriol*. 1990; doi: 10.1099/00207713-40-2-205.

22. Pitta DW, Pinchak WE, Dowd SE, Osterstock J, Gontcharova V, Youn E, et al.. Rumen bacterial diversity dynamics associated with changing from bermudagrass hay to grazed winter wheat diets. *Microb Ecol*. 2010; doi: 10.1007/s00248-009-9609-6.

23. Lee HJ, Jung JY, Oh YK, Lee SS, Madsen EL, Jeon CO. Comparative survey of rumen microbial communities and metabolites across one caprine and three bovine groups, using bar-coded pyrosequencing and 1H nuclear magnetic resonance spectroscopy. *Appl Environ Microbiol*. 2012; doi: 10.1128/AEM.00104-12.

24. Lopes LD, de Souza Lima AO, Taketani RG, Darias P, da Silva LRF, Romagnoli EM, et al.. Exploring the sheep rumen microbiome for carbohydrate-active enzymes. *Antonie van Leeuwenhoek, Int J Gen Mol Microbiol*. 2015; doi: 10.1007/s10482-015-0459-6.

25. Li J, Zhong H, Ramayo-Caldas Y, Terrapon N, Lombard V, Potocki-Veronese G, et

al.. A catalog of microbial genes from the bovine rumen unveils a specialized and diverse biomass-degrading environment. *Gigascience*. 2020; doi: 10.1093/gigascience/giaa057.

26. Seshadri R, Leahy SC, Attwood GT, Teh KH, Lambie SC, Cookson AL, et al.. Cultivation and sequencing of rumen microbiome members from the Hungate1000 Collection. *Nat Biotechnol*. 2018; doi: 10.1038/nbt.4110.

27. Crespo-Piazuelo D, Estellé J, Revilla M, Criado-Mesas L, Ramayo-Caldas Y, Óvilo C, et al.. Characterization of bacterial microbiota compositions along the intestinal tract in pigs and their interactions and functions. *Sci Rep*. 2018; doi: 10.1038/s41598-018-30932-6.

28. Han GG, Lee JY, Jin GD, Park J, Choi YH, Kang SK, et al.. Tracing of the fecal microbiota of commercial pigs at five growth stages from birth to shipment. *Sci Rep*. 2018; doi: 10.1038/s41598-018-24508-7.

29. Ransom-Jones E, Jones DL, McCarthy AJ, McDonald JE. The Fibrobacteres: An Important Phylum of Cellulose-Degrading Bacteria. *Microb Ecol*. 2012; doi: 10.1007/s00248-011-9998-1.

30. Newbold CJ, De la Fuente G, Belanche A, Ramos-Morales E, McEwan NR. The role of ciliate protozoa in the rumen. *Front Microbiol*. 2015; doi: 10.3389/fmicb.2015.01313.

31. Williams AG, Coleman GS. The Rumen Protozoa. Williams AG, Coleman GS, editors. New York, NY: Springer New York;

32. Johnson HP. A contribution to the morphology and biology of the Stentors. *J Morphol*. 1893; doi: 10.1002/jmor.1050080303.

33. Tartar V. The Biology of Stentor. Tartar V, editor. Oxford, London, New York, Paris: Pergamon Press;

34. Wichterman R. The Biology of Paramecium. Wichterman R, editor. New York: Springer US;

35. Cavalier-Smith T. The neomuran origin of archaebacteria, the negibacterial root of the universal tree and bacterial megaclassification. *Int J Syst Evol Microbiol*. 2002; doi: 10.1099/00207713-52-1-7.

36. Poulsen M, Schwab C, Borg Jensen B, Engberg RM, Spang A, Canibe N, et al.. Methylotrophic methanogenic Thermoplasmata implicated in reduced methane emissions from bovine rumen. *Nat Commun*. 2013; doi: 10.1038/ncomms2432.

37. Santos E de O, Thompson F. The family Succinivibrionaceae. In: Rosenberg E, DeLong EF, Lory S, Stackebrandt E, Thompson F, editors. *The Prokaryotes: Gammaproteobacteria*. Springer, Berlin, Heidelberg;

38. Gomez-Gil B, Thompson, Cristinane C. Matsumura Y, Sawabe T, Iida T, Christen R, Thompson F, et al.. The Family Vibrionaceae. In: Rosenberg E, DeLong EF, Lory S,

Stackebrandt E, Thompson F, editors. *The Prokaryotes: Gammaproteobacteria*. Springer, Berlin, Heidelberg;

39. Ivanova EP, Ng HJ, Webb HK. The family Pseudoalteromonadaceae. In: Rosenberg E, DeLong EF, Lory S, Stackebrandt E, Thompson F, editors. *The Prokaryotes: Gammaproteobacteria*. Springer, Berlin, Heidelberg;

40. Marchandin H, Jumas-Bilak E. The family Veillonellaceae. In: Rosenberg E, DeLong EF, Lory S, Stackebrandt E, Thompson F, editors. *The Prokaryotes: Firmicutes and Tenericutes*. Springer, Berlin, Heidelberg;

41. Shah HN, Collins MD. Reclassification of Bacteroides multiacidus (Mitsuoka, Terada, Watanabe and Uchida) in a new genus Mitsuokella, as Mitsuokella multiacidus comb. nov. *Zentralblatt fur Bakteriol Angew und Okol Microbiol Abt1 OrigC Hyg*. 1982; doi: 10.1016/S0721-9571(82)80006-9.

42. Kittelmann S, Pinares-Patiño CS, Seedorf H, Kirk MR, Ganesh S, McEwan JC, et al.. Two Different Bacterial Community Types Are Linked with the Low-Methane Emission Trait in Sheep. *PLoS One*. 2014; doi: 10.1371/JOURNAL.PONE.0103171.

43. Wallace RJ, Rooke JA, Duthie CA, Hyslop JJ, Ross DW, McKain N, et al.. Archaeal abundance in post-mortem ruminal digesta may help predict methane emissions from beef cattle. *Sci Rep*. 2014; doi: 10.1038/srep05892.

44. Morgavi DP, Martin C, Jouany JP, Ranilla MJ. Rumen protozoa and methanogenesis: Not a simple cause-effect relationship. *Br J Nutr*. 2012; doi: 10.1017/S0007114511002935.

45. Tapio I, Snelling TJ, Strozzi F, Wallace RJ. The ruminal microbiome associated with methane emissions from ruminant livestock. *J Anim Sci Biotechnol*. 2017; doi: 10.1186/s40104-017-0141-0.

46. Danielsson R, Dicksved J, Sun L, Gonda H, Müller B, Schnürer A, et al.. Methane production in dairy cows correlates with rumen methanogenic and bacterial community structure. *Front Microbiol*. 2017; doi: 10.3389/fmicb.2017.00226.

47. Shi W, Moon CD, Leahy SC, Kang D, Froula J, Kittelmann S, et al.. Methane yield phenotypes linked to differential gene expression in the sheep rumen microbiome. *Genome Res*. 2014; doi: 10.1101/gr.168245.113.

48. Wallace RJ, Rooke JA, McKain N, Duthie CA, Hyslop JJ, Ross DW, et al.. The rumen microbial metagenome associated with high methane production in cattle. *BMC Genomics*. 2015; doi: 10.1186/s12864-015-2032-0.

49. Aguinaga Casañas MA, Rangkasenee N, Krattenmacher N, Thaller G, Metges CC, Kuhla B. Methyl-coenzyme M reductase A as an indicator to estimate methane production from dairy cows. *J Dairy Sci*. 2015; doi: 10.3168/jds.2015-9310.

50. Janssen PH, Kirs M. Structure of the archaeal community of the rumen. *Appl Environ Microbiol*. 2008; doi: 10.1128/AEM.02812-07.

51. Newbold CJ, Lassalas B, Jouany JP. The importance of methanogens associated with ciliate protozoa in ruminal methane production in vitro. *Lett Appl Microbiol*. 1995; doi: 10.1111/j.1472-765X.1995.tb01048.x.

52. Qin WZ, Li CY, Kim JK, Ju JG, Song MK. Effects of defaunation on fermentation characteristics and methane production by rumen microbes in vitro when incubated with starchy feed sources. *Asian-Australasian J Anim Sci*. 2012; doi: 10.5713/ajas.2012.12240.

53. Schönhusen U, Zitnan R, Kuhla S, Jentsch W, Derno M, Voigt J. Effects of protozoa on methane production in rumen and hindgut of calves around time of weaning. *Arch Anim Nutr*. 2003; doi: 10.1080/00039420310001594423.

54. Guyader J, Eugène M, Nozière P, Morgavi DP, Doreau M, Martin C. Influence of rumen protozoa on methane emission in ruminants: a meta-analysis approach. *Animal*. 2014; doi: 10.1017/S1751731114001852.

55. Belanche A, de la Fuente G, Newbold CJ. Study of methanogen communities associated with different rumen protozoal populations. *FEMS Microbiol Ecol*. 2014; doi: 10.1111/1574-6941.12423.

56. Hackstein JHP, de Graaf RM. Anaerobic Ciliates and Their Methanogenic Endosymbionts. In: Hackstein JHP, editor. *(Endo)symbiotic Methanogenic Archaea*. 2nd ed. Berlin: Springer Berlin;

57. Roopnarain A, Mukhuba M, Adeleke R, Moeletsi M. Biases during DNA extraction affect bacterial and archaeal community profile of anaerobic digestion samples. *Biotech*. 2017; doi: 10.1007/s13205-017-1009-x.

58. Esquivel-Elizondo S, Ilhan ZE, Garcia-Peña EI, Krajmalnik-Brown R. Insights into Butyrate Production in a Controlled Fermentation System via Gene Predictions. *mSystems*. 2017; doi: 10.1128/msystems.00051-17.

59. Crable BR, Plugge CM, McInerney MJ, Stams AJM. Formate formation and formate conversion in biological fuels production. *Enzyme Res*. 2011; doi: 10.4061/2011/532536.

60. Hao L, Michaelsen TY, Singleton CM, Dottorini G, Kirkegaard RH, Albertsen M, et al.. Novel syntrophic bacteria in full-scale anaerobic digesters revealed by genome-centric metatranscriptomics. *ISME J*. 2020; doi: 10.1038/s41396-019-0571-0.

61. Brown TDK, Jones-Mortimer MC, Kornberg HL. The enzymic interconversion of acetate and acetyl-coenzyme A in Escherichia coli. *J Gen Microbiol*. 1977; doi: 10.1099/00221287-102-2-327.

62. Xie M, Chen HH, Nie SP, Yin JY, Xie MY. Gamma-aminobutyric acid increases the production of short-chain fatty acids and decreases pH values in mouse colon. *Molecules*. 2017; doi: 10.3390/molecules22040653.

63. Graham DE. 2-oxoacid metabolism in methanogenic CoM and CoB biosynthesis. *Methods Enzymol*. Academic Press Inc.;

64. Klüber HD, Conrad R. Effects of nitrate, nitrite, NO and N2O on methanogenesis and other redox processes in anoxic rice field soil. *FEMS Microbiol Ecol*. 2006; doi: 10.1111/j.1574-6941.1998.tb00482.x.

65. Yang C, Rooke JA, Cabeza I, Wallace RJ. Nitrate and inhibition of ruminal methanogenesis: Microbial ecology, obstacles, and opportunities for lowering methane emissions from ruminant livestock. *Front Microbiol*. 2016; doi: 10.3389/fmicb.2016.00132.

66. Lee C, Beauchemin KA. Une revue de l'ajout de nitrate dans l'alimentation des ruminants : Toxicité aux nitrates, émissions de méthane et performance de production. *Can J Anim Sci*. 2014; doi: 10.4141/CJAS-2014-069.

67. Cui X. The bacteriocidal effects of transition metal complexes containing the NO+ group on the food-spoilage bacterium Clostridium sporogenes. *FEMS Microbiol Lett*. 1992; doi: 10.1016/0378-1097(92)90133-9.

68. Zumft WG. The biological role of nitric oxide in bacteria. *Arch Microbiol*. 1993; doi: 10.1007/BF00292074.

69. Reed B, Dinn J, McCann S, Wilson P, O'Sullivan H, Weir DG, et al.. Alterations in mammalian cells induced by inactivation of vitamin B12 with nitrous oxide. In: Zagalak B, Friedrich W, editors. *Vitam B12*. Zürich: De Gruyter;

70. Klüber HD, Conrad R. Inhibitory effects of nitrate, nitrite, NO and N2O on methanogenesis by Methanosarcina barkeri and Methanobacterium bryantii. *FEMS Microbiol Ecol*. 1998; doi: 10.1111/j.1574-6941.1998.tb00484.x.

71. Tamames J, Puente-Sánchez F. SqueezeMeta, A Highly Portable, Fully Automatic Metagenomic Analysis Pipeline. *Front Microbiol*. 2019; doi: 10.3389/fmicb.2018.03349.

72. Stewart RD, Auffret MD, Warr A, Walker AW, Roehe R, Watson M. Compendium of 4,941 rumen metagenome-assembled genomes for rumen microbiome biology and enzyme discovery. *Nat Biotechnol*. 2019; doi: 10.1038/s41587-019-0202-3.

73. Rey J, Atxaerandio R, Ruiz R, Ugarte E, González-Recio O, Garcia-Rodriguez A, et al.. Comparison Between Non-Invasive Methane Measurement Techniques in Cattle. *Animals*. 2019; doi: 10.3390/ani9080563.

74. López-Paredes J, Goiri I, Atxaerandio R, García-Rodríguez A, Ugarte E, Jiménez-Montero JA, et al.. Mitigation of greenhouse gases in dairy cattle via genetic selection: 1. Genetic parameters of direct methane using noninvasive methods and proxies of methane. *J Dairy Sci*. .; 2020; doi: 10.3168/jds.2019-17597.

75. Aitchison J. The Statistical Analysis of Compositional Data. *J R Stat Soc Ser B*. 1982; doi: 10.1111/j.2517-6161.1982.tb01195.x.

76. Greenacre M. Package "easyCODA". Compositional Data Analysis in Practice. Chapman & Hall / CRC Press; p. 35.

77. Palarea-Albaladejo J, Martín-Fernández JA. ZCompositions - R package for multivariate imputation of left-censored data under a compositional approach. Chemom. Intell. Lab. Syst.

78. Oksanen J, Blanchet FG, Friendly M, Kindt R, Legendre P, Mcglinn D, et al.. vegan: Community Ecology Package. R package version 2.5-6.

79. Anderson MJ. A new method for non-parametric multivariate analysis of variance. *Austral Ecol*. 2001; doi: 10.1111/j.1442-9993.2001.01070.pp.x.

80. McArdle BH, Anderson MJ. Fitting multivariate models to community data: A comment on distance-based redundancy analysis. *Ecology*. 2001; doi: 10.1890/0012-9658(2001)082[0290:FMMTCD]2.0.CO;2.

81. Ritchie ME, Phipson B, Wu D, Hu Y, Law CW, Shi W, et al.. limma powers differential expression analyses for RNA-sequencing and microarray studies. *Nucleic Acids Res*. 2015; doi: 10.1093/nar/gkv007.

82. Quinn TP, Richardson MF, Lovell D, Crowley TM. Propr: An R-package for Identifying Proportionally Abundant Features Using Compositional Data Analysis. *Sci Rep*. 2017; doi: 10.1038/s41598-017-16520-0.

83. Erb I, Notredame C. How should we measure proportionality on relative gene expression data? *Theory Biosci*. 2016; doi: 10.1007/s12064-015-0220-8.

84. Kamada T, Kawai S. An algorithm for drawing general undirected graphs. *Inf Process Lett*. 1989; doi: 10.1016/0020-0190(89)90102-6.

85. López-García A; Saborío-Montero A; Gutiérrez-Rivas M; Atxaerandio R; Goiri I; García-Rodríguez A; Jiménez-Montero JA; González C; Tamames J; Puente-Sánchez F; Serrano M; Carrasco R; Óvilo C; Gonzalez-Recio O: Supporting data for "Fungal and ciliate protozoa are the main rumen microbes associated with methane emissions in dairy cattle" GigaScience Database. 2021. http://dx.doi.org/10.5524/100950

15 Tables

Table 1: F statistic and P-values for stage of lactation (SL), number of lactation (NL) and methane emission (CH4) variables (added sequentially) and P-values from PERMANOVA of the entire dataset (i.e., including all superkingdoms).

		F statistic	R^2	*P*-value*
Phylum	*SL*	6.1	0.014	*<0.01*
	NL	1.4	0.003	0.11
	CH4	2.8	0.019	*<0.01*
Class	*SL*	5.6	0.013	*<0.01*
	NL	1.5	0.003	0.07

	CH4	2.4	0.016	*<0.01*
Order	*SL*	5.4	0.012	*<0.01*
	NL	1.7	0.004	*0.03*
	CH4	2.3	0.016	*<0.01*
Family	*SL*	4.9	0.011	*<0.01*
	NL	1.6	0.004	*0.03*
	CH4	2.1	0.014	*<0.01*
Genus	*SL*	4.0	0.009	*<0.01*
	NL	1.4	0.003	*0.03*
	CH4	1.7	0.012	*<0.01*
KEGG	*SL*	5.3	0.012	*<0.01*
	NL	2.0	0.004	*0.02*
	CH4	2.4	0.016	*<0.01*

**Significance level was considered 0.05. P-values lower than this significance levels are in italics.*

16 Figure captions

Figure 1: Average relative abundance of genera. Average relative abundance of core microbial taxa, including those classified only to family level (i.e., unclassified genera), which represent a 60.2% of total abundance.

Figure 2: **Metagenome functionality**. TreeMap distribution of functionality abundances classified as KEGG pathways (left) and BRITE hierarchies (right) associated with core KEGG subcomposition.

Figure 3. Fitted surface representation of Principal Component Analysis. Dots represent the samples using euclidean distances of CLR-transformed taxa abundances, coloured by CH4 levels. CH_4 emissions (ppm) corrected by number and stage of lactation are represented as smooth fitting following a generalized additive model (GAM) (–). *Dev. Explained*: variability explained by GAM; *P-val*: approximate significance of the smooth terms being zero (α=0.05).

Figure 4. Volcano plots. Volcano plot representing the differential abundance (DA) of genera (A) and KEGGs (B) between LOW and HIGH groups from limma. Significance

thresholds were established at *adj.P-val* = 0.05 and $\log_2 FC = \pm 0.5$. • Significant features with DA above the fold change (FC) threshold. • Significant features with DA below the FC threshold. • Non-significant features with DA above the FC threshold. • Non-significant features with DA below the FC threshold.

Figure 5. **Taxonomy interaction network**. Pairwise proportionalities between genera with $|\rho_p| \geq 0.4$. Superkingdom: △ Archaea; □ Bacteria; ○ Eukaryota. / CH_4 association: ▬ HIGH CH_4; ▬ LOW CH_4; ▬ No CH_4 associated. / Proportionality sense: ↔ direct (> 0); ↔ inverse (< 0).

Figure 6. Functionality interaction network. Presented pairwise proportionalities between KEGGs with $|\rho_p| \geq 0.7$/ Participation in methane metabolism: □ ko00680 (direct or indirect part.); ○ Other (no part.) / CH_4 association: ▬ HIGH CH_4; ▬ LOW CH_4; ▬ No CH_4 associated. / Proportionality sense: ↔ direct (> 0); ↔ inverse (< 0). Clusters are indicated as L (KEGGs associated to LOW methane), H (KEGGs associated to HIGH methane) and N (KEGGs not related to methane emissions).

Figure 7: **Taxonomy of ko00680 KEGGs**. Relative abundance of KEGGs present in ko00680 pathway for each phylum in Archaea (▬), Bacteria (▬) and Eukaryota (▬) superkingdoms. Relative abundance of each ko00680-KEGG respect to the sum of reads mapped to all ko00680-KEGGs.

Figure 8. Taxonomic distribution of DA KEGGs. Red density scale represents KEGGs over-abundant (OA) in HIGH emitters; Blue density scale represents KEGGs OA in LOW emitters. More intense colors mean a higher number of reads assigned to one phylum. Superkingdom: • Archaea; • Bacteria; • Eukaryota.

Supplementary Figure 1: **Feature counts distribution**. Features with zero counts, singletons, doubletons and 3 or more counts per sample. A) Count distribution in raw taxonomy table (87% sparsity); B) Count distribution in filtered taxonomy table (68% sparsity); C) Count distribution in raw KEGG table (72% sparsity); D) Count distribution in filtered KEGG table (39% sparsity). Filtering processes removed less than 1% of total reads in both datasets.

Supplementary Figure 2: **Phyla relative abundance per sample**. Samples are sorted from lowest to highest RA of *Bacteroidetes*.

Figure 1

Click here to access/download;Figure;Fig1.tif

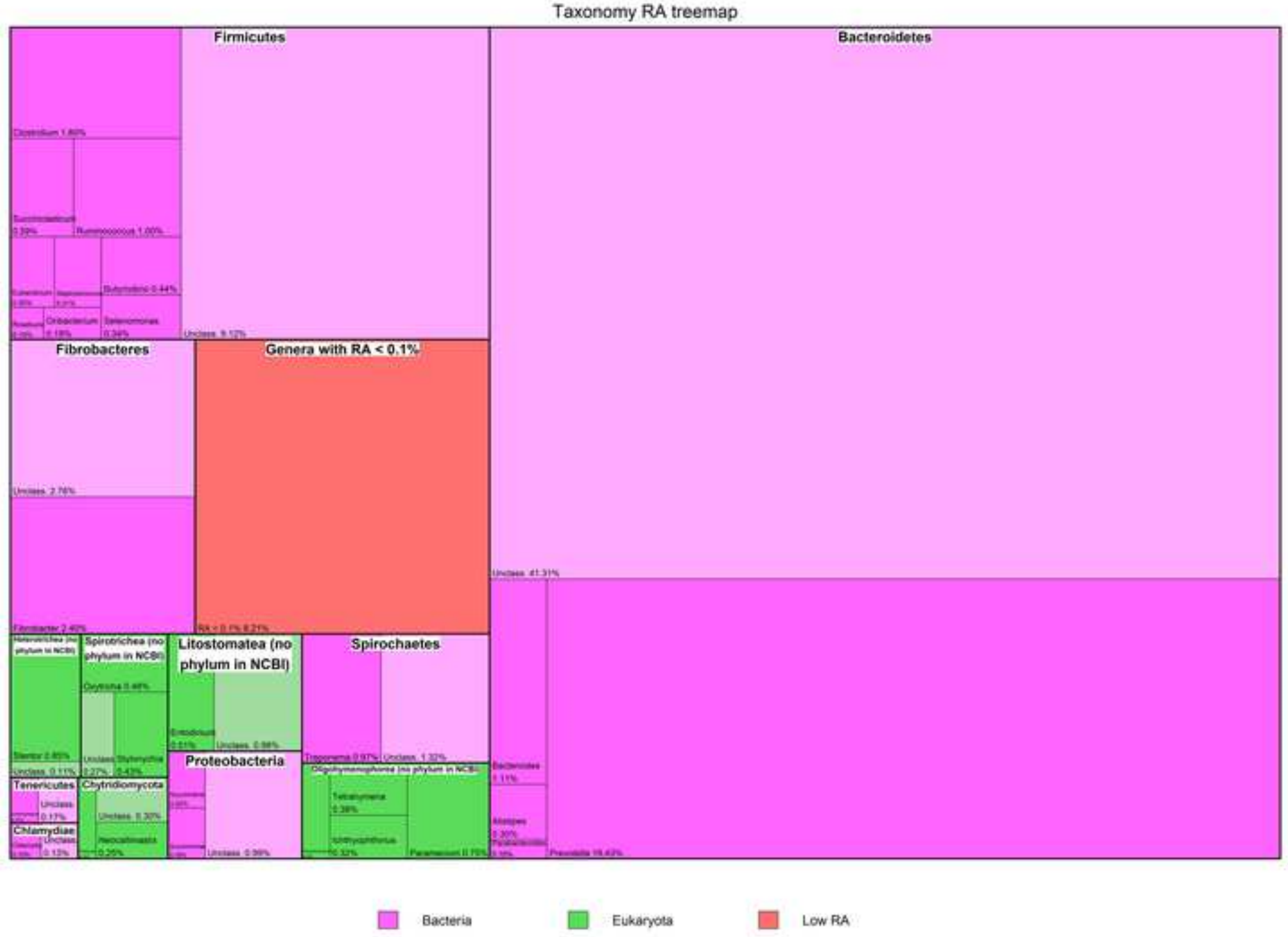

Figure 2

Click here to access/download;Figure;Fig2.tif

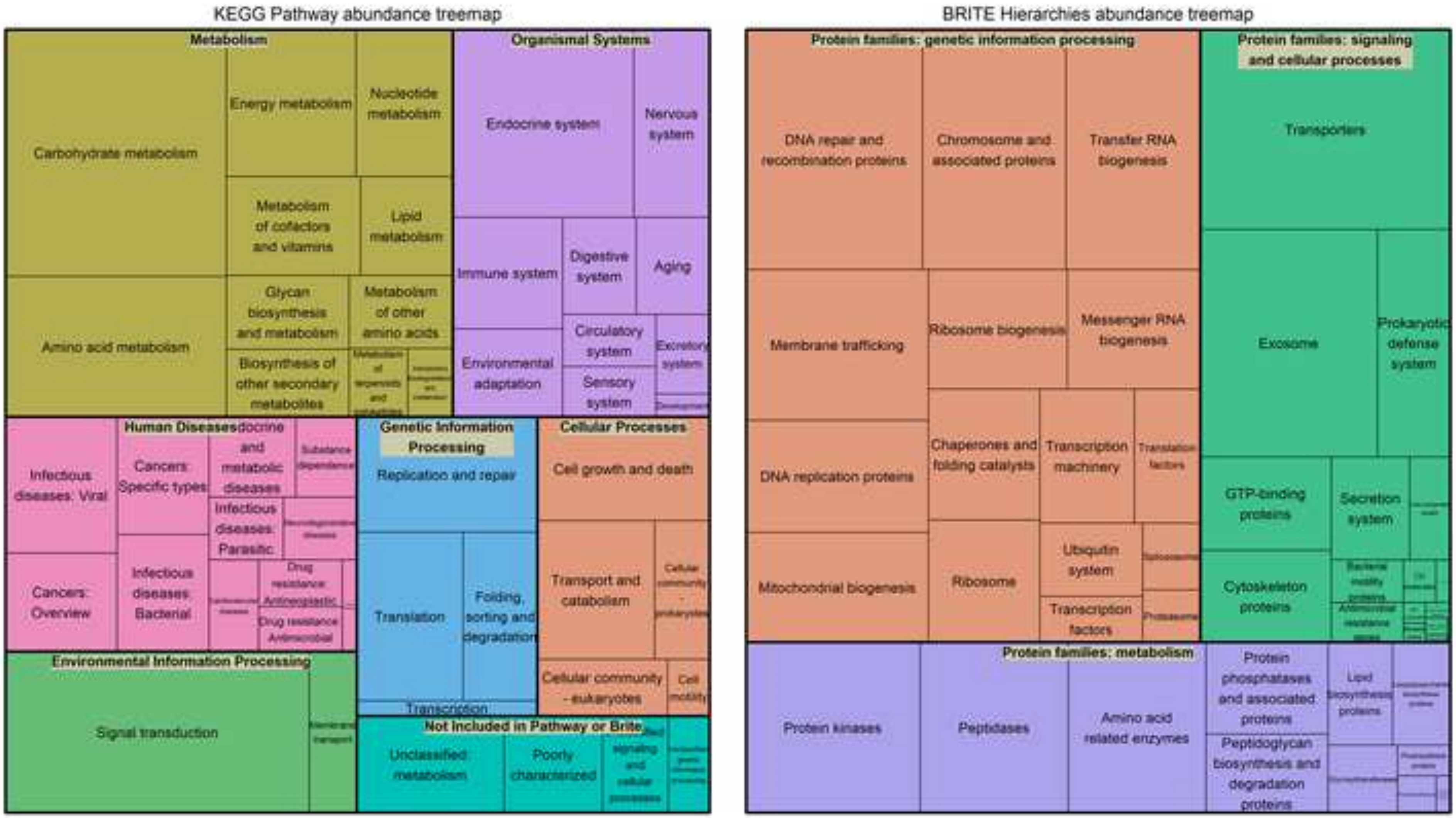

Figure 3

Click here to access/download;Figure;Fig3.tif

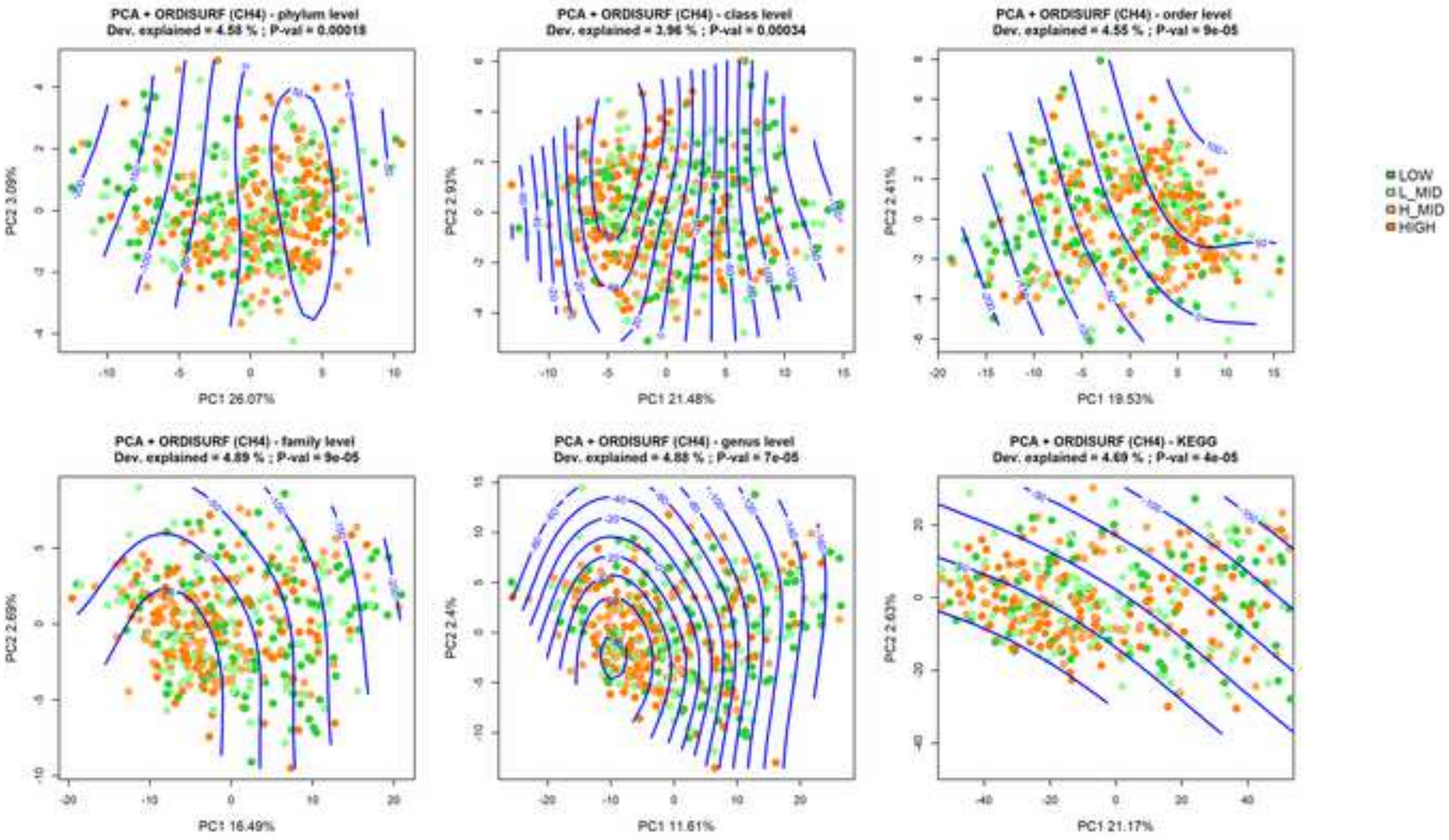

Figure 4

Click here to access/download;Figure;Fig4.tif

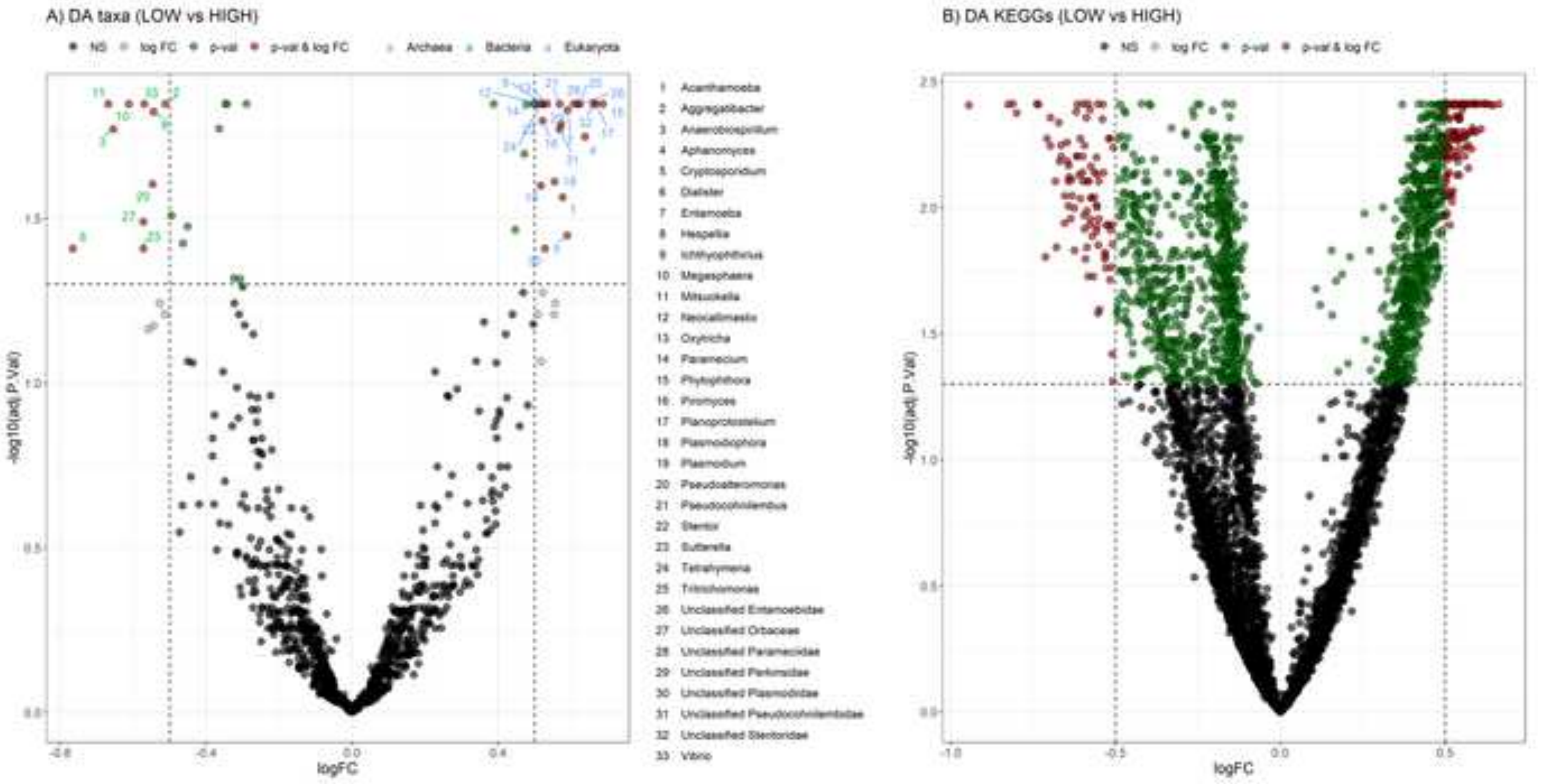

Figure 5

Click here to access/download;Figure;Fig5.tif

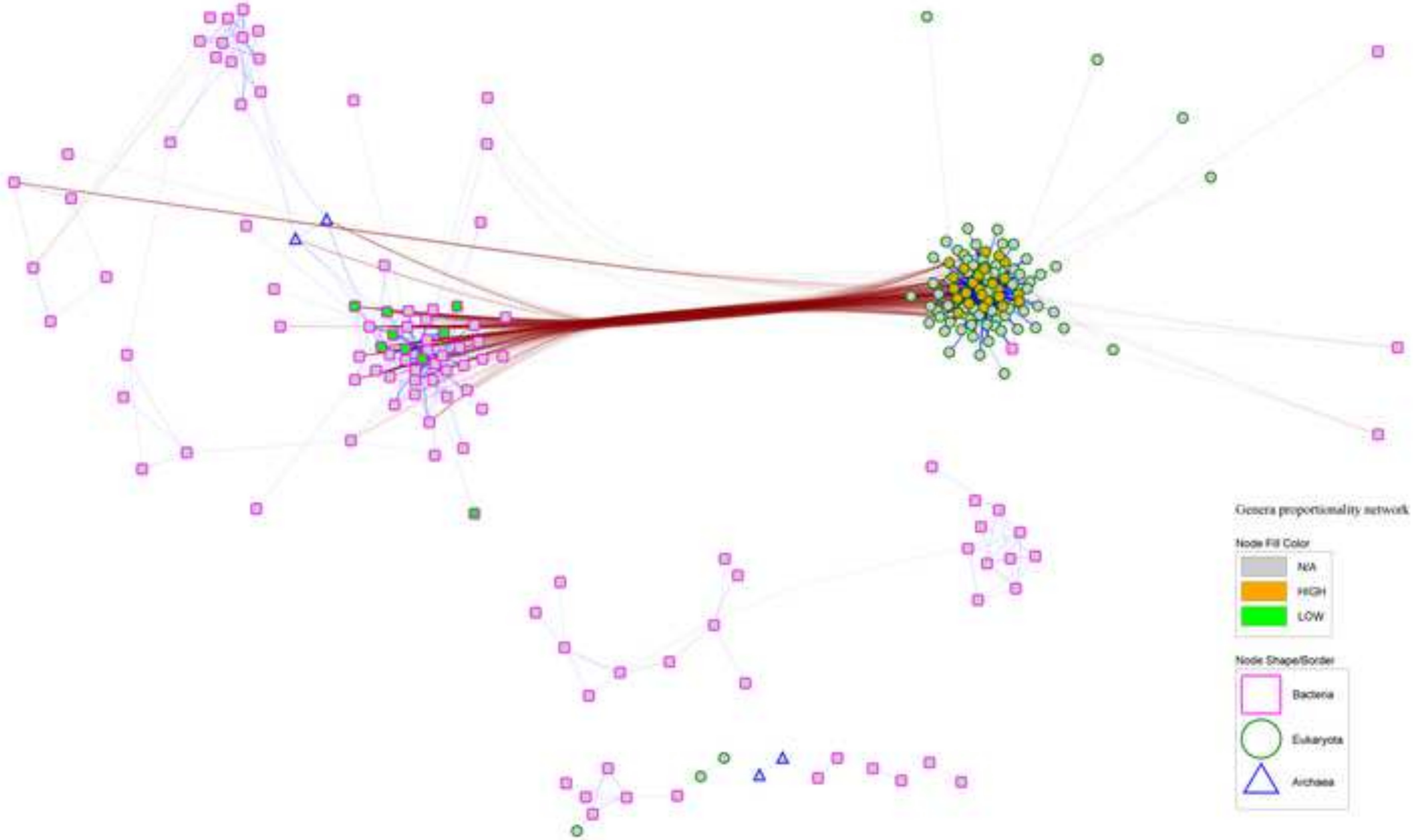

Figure 6

Click here to access/download;Figure;Fig6.tif

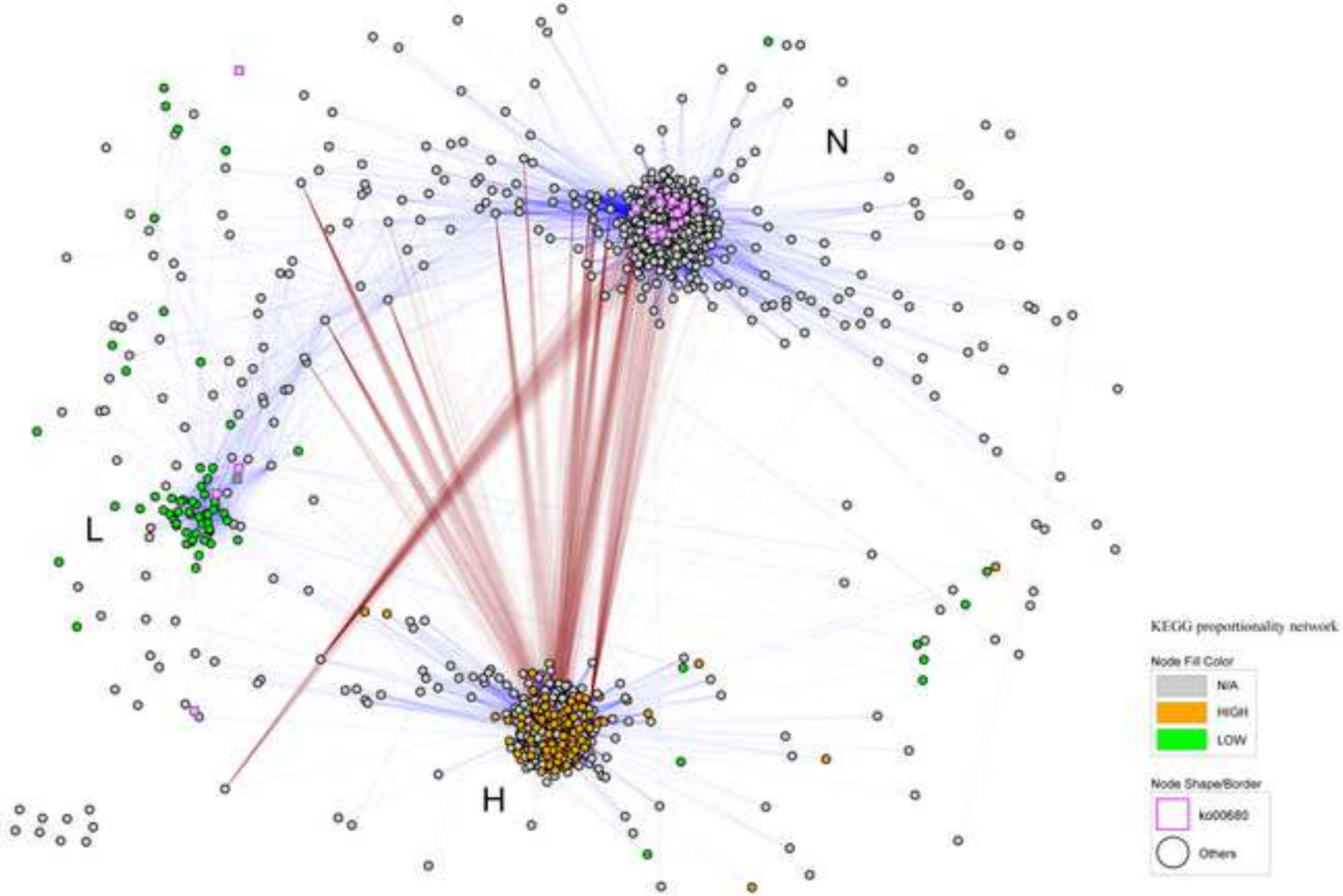

Figure 7

Click here to access/download;Figure;Fig7.tif

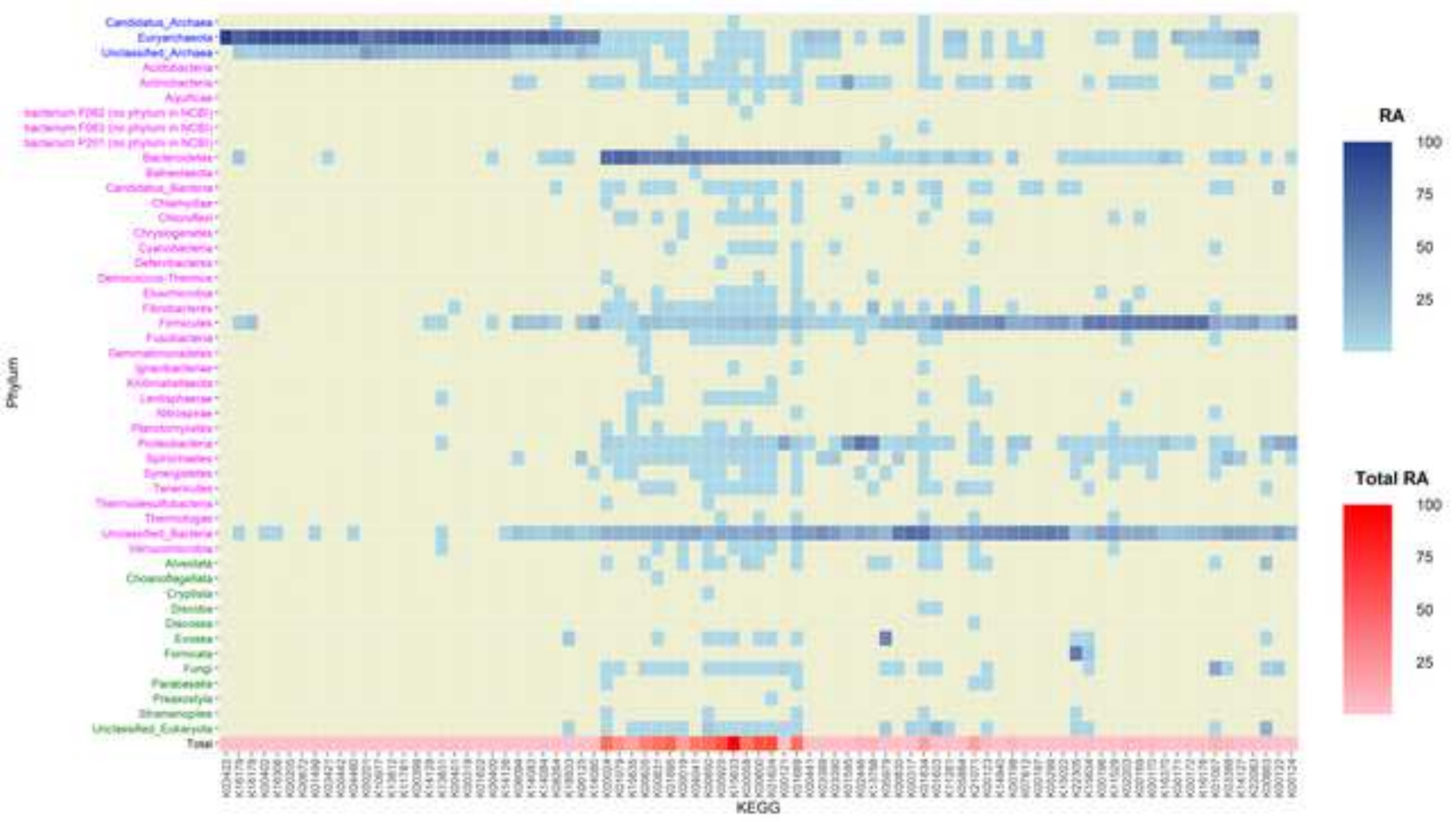

Figure 8

Click here to access/download;Figure;Fig8.tif

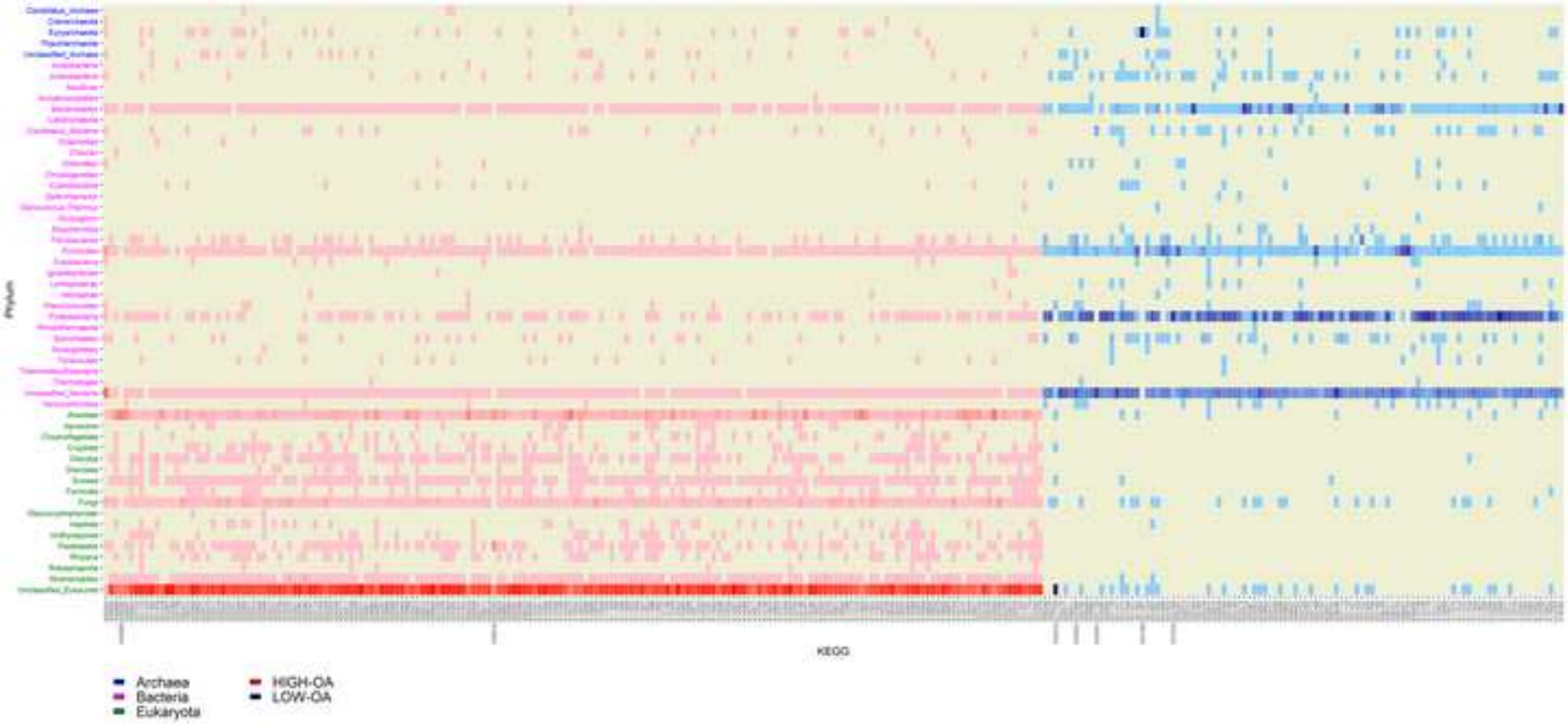

Supplementary Material FigS1

Click here to access/download
Supplementary Material
SFig1.tif

Supplementary Material FigS2

Click here to access/download
Supplementary Material
SFig2.tif

Click here to access/download;Personal Cover;Cover_letter_new.docx

GOBIERNO DE ESPAÑA

INIA Instituto Nacional de Investigación y Tecnología Agraria y Alimentaria

DEPARTAMENTO DE MEJORA GENETICA ANIMAL

Madrid, 28th October, 2021

Dear Editor Nicole Nogoy,

herewith, we re-submit our manuscript entitled "FUNGAL AND CILIATE PROTOZOA ARE THE MAIN RUMEN MICROBES ASSOCIATED WITH METHANE EMISSIONS IN DAIRY CATTLE", to be considered for publication in GigaScience.

The manuscript was previously submitted to GigaScience with manuscript ID: GIGA-D-21-00239. The manuscript was reviewed by three reviewers. Two of them recommended acceptance, whereas only one rejection. Unfortunately, the final decision was rejection. However, you kindly opened the door for a resubmission of the manuscript if we could address this reviewer's concerns in a full cover letter.

Hence, we took this opportunity and resubmit the manuscript explaining that we strongly disagree with the reasons given by the reviewer that led to rejection of the manuscript:

1) the reviewer stated that the way we annotated genes is not correct. He said "*I do not think it is at all clear that a DIAMOND search against the NR database (which is full of errors) will accurately reconstruct rumen taxonomy, nor do I believe that a DIAMOND search against KEGG or COG will reconstruct rumen function*". And he provided some reference as example on how to properly do the search. Our method is actually the same procedure as the one used in the references he provided as an example on how to do this (which he actually coauthored): (https://www.nature.com/articles/s41467-018-03317-6). In this manuscript they stated: "*Proteins were predicted using Prodigal (v.2.6.3) with option '-p meta'. Using DIAMOND, each protein was searched against KEGG (downloaded on 15 September 2018), UniRef100, UniRef90 and UniRef50 (downloaded 3 October 2018), and CAZy (dbCAN2 version, 31 July 2018). The protein predictions were clustered by CD-HIT[68](v.4.7) at 100%, 90% and 50% identity, mirroring similar methods at UniRef.*".
The reviewer assumed that the pipeline is not correct, but it actually follows the same procedure as the one cited by the reviewer as reference. The reviewer did not take the time to read how our pipeline works, which is fully described elsewhere: e.g. https://www.frontiersin.org/articles/10.3389/fmicb.2018.03349/full, https://bmcbioinformatics.biomedcentral.com/articles/10.1186/s12859-020-03703-2. Detailed descriptions can be also found in the manual (https://github.com/jtamames/SqueezeMeta/blob/master/SqueezeMeta_manual_v1.4.0.pdf).

2) The second concern of the reviewer was: "*The data presented directly contradict previous results (e.g. https://bmcgenomics.biomedcentral.com/articles/10.1186/s12864-015-2032-0) and so the authors need to ensure they are sound*." The manuscript he provided used results based on 16S and 18S RNA amplicons using abundances from qPCR. I'm not going to give details on how troublesome using PCR products from different amplicons can be at comparing bacteria, archaea and protozoa. His manuscript also used the GREENGENES database which has not been properly updated in the last decade. On the other hand, our study is free from possible bias caused by PCR, because we are using a metagenomic approach which is PCR free, and can properly compare relative abundances of bacteria and eukaryotes. Our results are in agreement with

CORREO ELECTRÓNICO:
adrian.lopez@inia.es

Ctra. de La Coruña, Km. 7,5
28040 MADRID
TEL: 91 347 14 92
FAX: 91 347 87 43

other studies that do not find a clear relationship between methane emissions and archaea relative abundance in the rumen (e.g. Shi W et al. 2014 Methane yield phenotypes linked to differential gene expression in the sheep rumen microbiome. Genome Res. 2014; doi: 10.1101/gr.168245.113. and Aguinaga Casañas MA, et al 2015. Methyl-coenzyme M reductase A as an indicator to estimate methane production from dairy cows. J Dairy Sci. 2015; doi: 10.3168/jds.2015-9310).
However, it must be pointed out that not-significant association does not imply absence of association, and therefore our results are not in contradiction with the reference provided by the reviewer. Besides, our results are in agreement with many other studies that reported an important role of fungi and protozoa with methane emissions, which we cited in our manuscript. For instances:
-Newbold CJ, et al. 2015 The role of ciliate protozoa in the rumen. Front Microbiol. 2015; doi:678 10.3389/fmicb.2015.01313.
-Williams AG and Coleman GS. The Rumen Protozoa. New York, NY: Springer New York;
and also Williams CL, et al. 2020. Rumen Protozoa Play a Significant Role in Fungal Predation and Plant Carbohydrate Breakdown. Front. Microbiol. 11:720. doi: 10.3389/fmicb.2020.00720 (actually not cited in the manuscript).
Thus, our study does not contradict previous results, not even those from the reviewer, and are actually in agreement with many other studies.

3) Finally, Dr. Watson suggested in his review that we should use some specific rumen microbiome gene catalogues. Interestingly, these reference databases were built by the reviewer and his collaborators. We believe this may incur in some conflict of interest at evaluating the manuscript. His group is currently working on a similar topic, even building a large database of rumen microbiomes using ONT. The other two reviewers are also experienced bioinformaticians working with microbial communities and they did not report any concerns with the methods in our article. Nonetheless, in order to double check the reviewer's concern, we used the Hungate database as reference in our pipeline. Below is a figure that compares the number of reads mapped to the KEGGs that resulted significant in our statistical analysis using eaither the NCBI or the Hungate databases. The figure shows a correlation=1 for the number of reads assigned from each reference data base. It also shows that the regression is exactly linear with x=y. This is a proof of concept that demonstrates that we obtain the same results with either database, and proves the reviewer wrong.

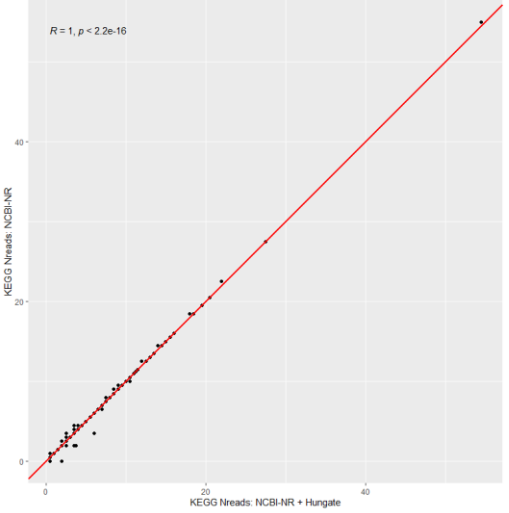

CORREO ELECTRÓNICO:
ovilo@inia.es

Ctra. de La Coruña, Km. 7,5
28040 MADRID
TEL: 91 347 14 92
FAX: 91 347 87 43

Given the responses and analyses above, we believe that we have fully addressed the comments from the three reviewers or otherwise provided convincing reasons when appropriate.
Therefore, I would like to kindly ask to reconsider the decision made on our manuscript.

Sincerely,

Oscar González-Recio, on the behalf of my coauthors and myself

CORREO ELECTRÓNICO:

adrian.lopez@inia.es

Ctra. de La Coruña, Km. 7,5
28040 MADRID
TEL: 91 347 14 92
FAX: 91 347 87 43
