# Supplementary material for: Fungal and ciliate protozoa are the main rumen microbes associated with methane emissions in dairy cattle
Source: Gigascience. 2022 Jan 25;11:giab088. doi: 10.1093/gigascience/giab088 (PMC8848325; doi:10.1093/gigascience/giab088)
Supplement: giab088_Supplemental_Files [file giab088_supplemental_files.zip › Suppl_Data_S2.pdf]

Supplementary Information

1 Supplementary Information 1: Feature selection by taxonomic classification

Taxonomy filtering was applied in order to purge features not interesting for microbiome analysis. Purged sequences were mapped to animalia, plantae and virus kingdoms (**Table 1**). Sequences unmapped to family level (unclassified) were also removed.

Table 1: Purged clades from taxonomy table

Acanthocephala	***Cnidaria***	***Nematoda***	***Rhodophyta***
Annelida	***Ctenophora***	***Nematomorpha***	***Rhopaluridae***
Arthropoda	***Dicyemida***	***Nemertea***	***Rotifera***
Brachiopoda	***Echinodermata***	***Onychophora***	***Streptophyta***
Bryozoa	***Entoprocta***	***Placozoa***	***Tardigrada***
Chaetognatha	***Gnathostomulida***	***Platyhelminthes***	***Xenacoelomorpha***
Chlorophyta	***Hemichordata***	***Porifera***	***Unclassified***
Chordata	***Mollusca***	***Priapulimorpha***	***Virus***

2 Supplementary Information 2: Data pruning by prevalence filtering

Microbial features were filtered based on Roesch et al. (2019) [1] to remove low prevalence features. Steps followed by this pipeline (**Figure 1**) are now explained:

1) Prevalence subcomposition preparation: Data subcompositions were created from removal of less prevalent features. Different prevalence thresholds were applied starting at 0% (i.e., features present in less than 5% of samples), and in 5% increments up to 95% (i.e., features present in less than 95% of samples). A total of 20 subcompositions were prepared.

2) Core microbiome selection: We used methane emissions as the phenotypic variable to be predicted in a random forest regression including microbiome features, with NL and SL as covariates. Every subcomposition was used as input for a Random Forest model (randomForest package in R) [2] using methane as response variable. The optimal subcomposition, i.e., the one classifying phenotypes with the lowest out-of-bag (OOB) error, while minimizing total read loss, will be chosen as core microbiome. Our lowest OOB error showed at a prevalence threshold of 20% for taxonomy dataset, and 15% for functionality dataset.

3) Addition of low-prevalence/high-importance (LP/hVI) features: After choosing the core microbiome, the inverse subcomposition of LP features, was used for an additional step to recover low prevalence features with potential large importance. Another Random Forest was then performed solely with these discarded features, and LP features with a VI > 1 that were present in at 6 samples were kept for further analyses as core microbiome.

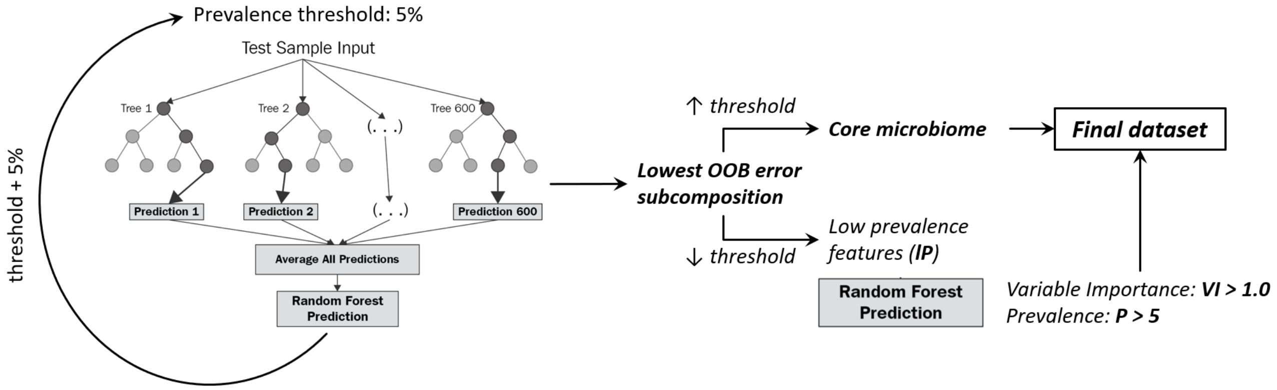

Figure 1. Scheme of the prevalence filter pipeline.

3 References:

1) Roesch, L.F.W., Dobbler, P.T., Pylro, V.S., Kolaczkowski, B., Drew, J.C., Triplett, E.W. (2020). pime: A package for discovery of novel differences among microbial communities. *Mol Ecol Resour. Blackwell Publishing Ltd* 20:415–28.

2) Liaw, A., Wiener, M. (2002). Classification and Regression by randomForest.
