# Supplementary figures and images for: Fungal and ciliate protozoa are the main rumen microbes associated with methane emissions in dairy cattle
Source: Gigascience. 2022 Jan 25;11:giab088. doi: 10.1093/gigascience/giab088 (PMC8848325; doi:10.1093/gigascience/giab088)

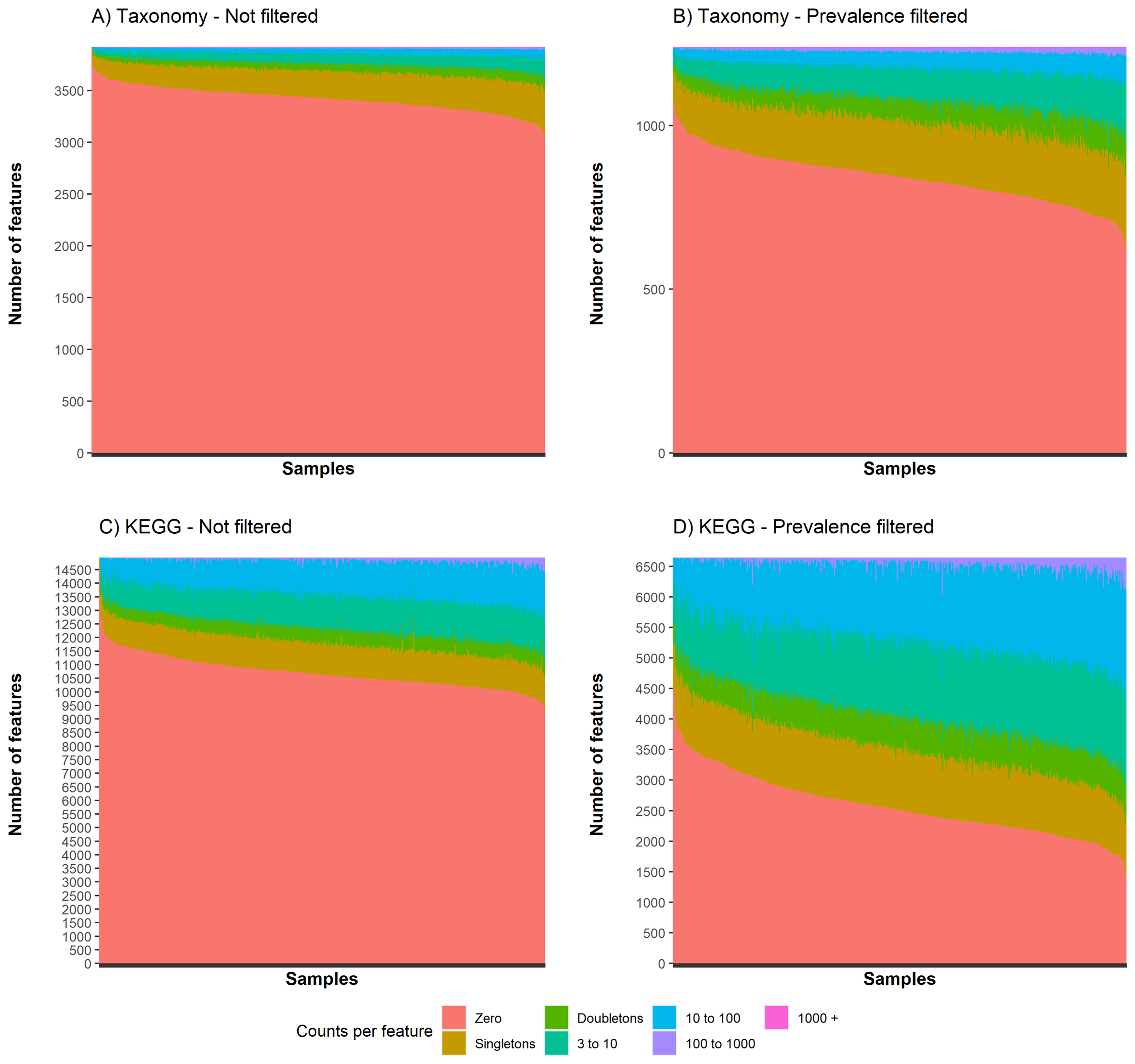

Supplement: giab088_Supplemental_Files [file giab088_supplemental_files.zip › SFig1.tif]

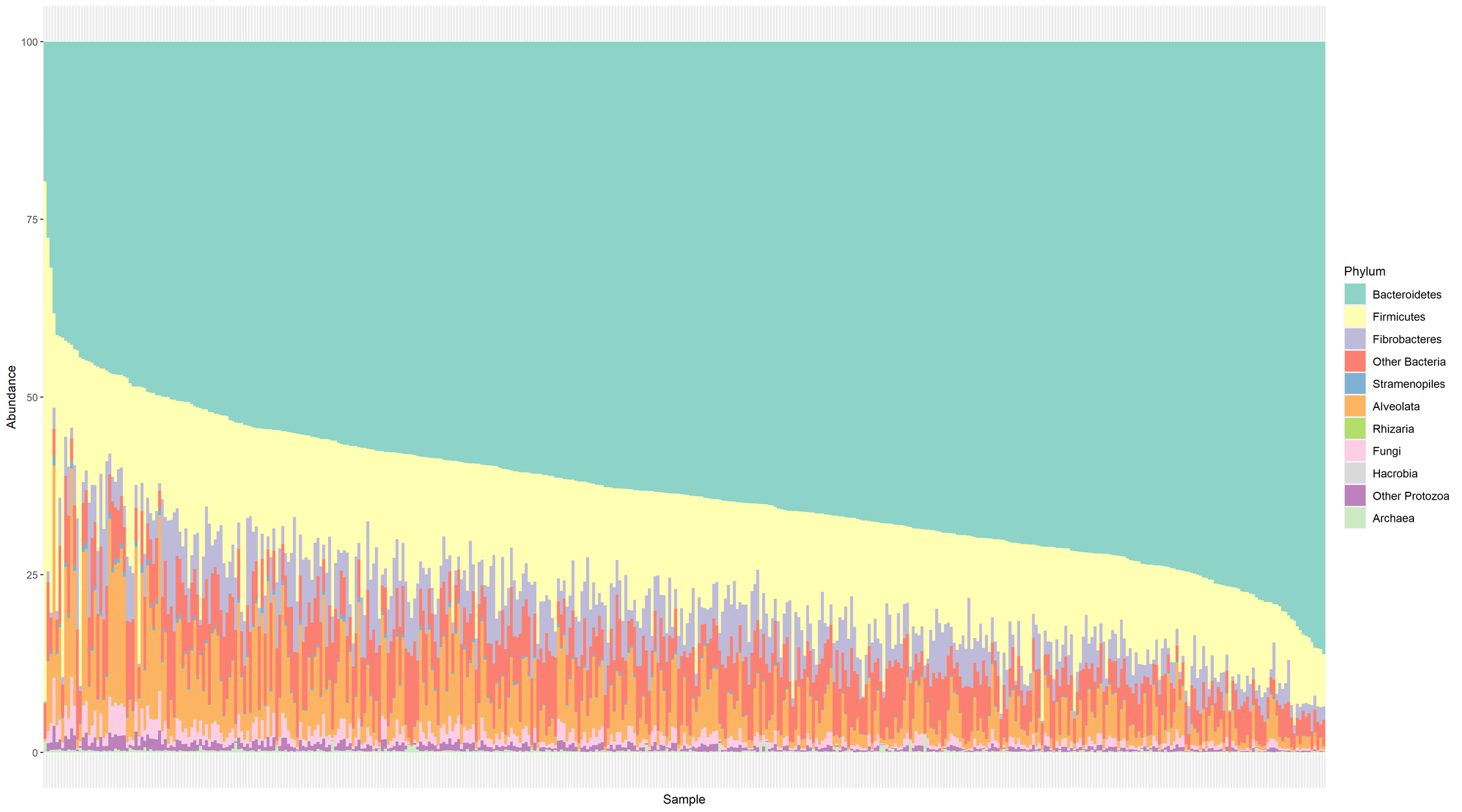

Supplement: giab088_Supplemental_Files [file giab088_supplemental_files.zip › SFig2.tif]
