# Supplementary material for: Fungal and ciliate protozoa are the main rumen microbes associated with methane emissions in dairy cattle
Source: Gigascience. 2022 Jan 25;11:giab088. doi: 10.1093/gigascience/giab088 (PMC8848325; doi:10.1093/gigascience/giab088)
Supplement: giab088_Response_to_Reviewer_Comments_Original_Submission [file giab088_response_to_reviewer_comments_original_submission.pdf]

Author's Response To Reviewer Comments

Close

herewith, we re-submit our manuscript entitled “FUNGAL AND CILIATE PROTOZOA ARE THE MAIN RUMEN MICROBES ASSOCIATED WITH METHANE EMISSIONS IN DAIRY CATTLE”, to be considered for publication in GigaScience.

The manuscript was previously submitted to GigaScience with manuscript ID: GIGA-D-21-00239. The manuscript was reviewed by three reviewers. Two of them recommended acceptance, whereas only one rejection. Unfortunately, the final decision was rejection. However, you kindly opened the door for a resubmission of the manuscript if we could address this reviewer’s concerns in a full cover letter.

Hence, we took this opportunity and resubmit the manuscript explaining that we strongly disagree with the reasons given by the reviewer that led to rejection of the manuscript:

1) the reviewer stated that the way we annotated genes is not correct. He said "I do not think it is at all clear that a DIAMOND search against the NR database (which is full of errors) will accurately reconstruct rumen taxonomy, nor do I believe that a DIAMOND search against KEGG or COG will reconstruct rumen function". And he provided some reference as example on how to properly do the search. Our method is actually the same procedure as the one used in the references he provided as an example on how to do this (which he actually coauthored): (https://www.nature.com/articles/s41467-018-03317-6). In this manuscript they stated: "Proteins were predicted using Prodigal (v.2.6.3) with option ‘-p meta’. Using DIAMOND, each protein was searched against KEGG (downloaded on 15 September 2018), UniRef100, UniRef90 and UniRef50 (downloaded 3 October 2018), and CAZy (dbCAN2 version, 31 July 2018). The protein predictions were clustered by CD-HIT68(v.4.7) at 100%, 90% and 50% identity, mirroring similar methods at UniRef.".
The reviewer assumed that the pipeline is not correct, but it actually follows the same procedure as the one cited by the reviewer as reference. The reviewer did not take the time to read how our pipeline works, which is fully described elsewhere: e.g.
https://www.frontiersin.org/articles/10.3389/fmicb.2018.03349/full,
https://bmcbioinformatics.biomedcentral.com/articles/10.1186/s12859-020-03703-2. Detailed descriptions can be also found in the manual
(https://github.com/jtamames/SqueezeMeta/blob/master/SqueezeMeta_manual_v1.4.0.pdf).

2) The second concern of the reviewer was: "The data presented directly contradict previous results (e.g. https://bmcgenomics.biomedcentral.com/articles/10.1186/s12864-015-2032-0) and so the authors need to ensure they are sound." The manuscript he provided used results based on 16S and 18S RNA amplicons using abundances from qPCR. I'm not going to give details on how troublesome using PCR products from different amplicons can be at comparing bacteria, archaea and protozoa. His manuscript also used the GREENGENES database which has not been properly updated in the last decade. On the other hand, our study is free from possible bias caused by PCR, because we are using a metagenomic approach which is PCR free, and can properly compare relative abundances of bacteria and eukaryotes. Our results are in agreement with other studies that do not find a clear relationship between methane emissions and archaea relative abundance in the rumen (e.g. Shi W et al. 2014 Methane yield phenotypes linked to differential gene expression in the sheep rumen microbiome. Genome Res. 2014; doi: 10.1101/gr.168245.113. and Aguinaga Casañas MA, et al 2015. Methyl-coenzyme M reductase A as an indicator to estimate methane production from dairy cows. J Dairy Sci. 2015; doi: 10.3168/jds.2015-9310).
However, it must be pointed out that not-significant association does not imply absence of association, and therefore our results are not in contradiction with the reference provided by the reviewer. Besides, our results are in agreement with many other studies that reported an important role of fungi and protozoa with methane emissions, which we cited in our manuscript. For instances:
-Newbold CJ, et al. 2015 The role of ciliate protozoa in the rumen. Front Microbiol. 2015; doi:678 10.3389/fmicb.2015.01313.
-Williams AG and Coleman GS. The Rumen Protozoa. New York, NY: Springer New York;
and also Williams CL, et al. 2020. Rumen Protozoa Play a Significant Role in Fungal Predation and Plant

Carbohydrate Breakdown. Front. Microbiol. 11:720. doi: 10.3389/fmicb.2020.00720 (actually not cited in the manuscript).
Thus, our study does not contradict previous results, not even those from the reviewer, and are actually in agreement with many other studies.

3) Finally, Dr. Watson suggested in his review that we should use some specific rumen microbiome gene catalogues. Interestingly, these reference databases were built by the reviewer and his collaborators. We believe this may incur in some conflict of interest at evaluating the manuscript. His group is currently working on a similar topic, even building a large database of rumen microbiomes using ONT. The other two reviewers are also experienced bioinformaticians working with microbial communities and they did not report any concerns with the methods in our article. Nonetheless, in order to double check the reviewer's concern, we used the Hungate database as reference in our pipeline. Below is a figure that compares the number of reads mapped to the KEGGs that resulted significant in our statistical analysis using eaither the NCBI or the Hungate databases. The figure shows a correlation=1 for the number of reads assigned from each reference data base. It also shows that the regression is exactly linear with x=y. This is a proof of concept that demonstrates that we obtain the same results with either database, and proves the reviewer wrong.

Given the responses and analyses above, we believe that we have fully addressed the comments from the three reviewers or otherwise provided convincing reasons when appropriate.
Therefore, I would like to kindly ask to reconsider the decision made on our manuscript.

Sincerely,

Oscar González-Recio, on the behalf of my coauthors and myself

Close
