# Supplementary material for: Fungal and ciliate protozoa are the main rumen microbes associated with methane emissions in dairy cattle
Source: Gigascience. 2022 Jan 25;11:giab088. doi: 10.1093/gigascience/giab088 (PMC8848325; doi:10.1093/gigascience/giab088)
Supplement: giab088_Reviewer_1_Report_Original_Submission — danielagaiagaio Gaio -- 9/5/2021 Reviewed [file giab088_reviewer_1_report_original_submission.pdf]

Reviewer Report

Title: Fungal and ciliate protozoa are the main rumen microbes associated with methane emissions in dairy cattle

Version: Original Submission **Date:** 9/5/2021

Reviewer name: danielagaiagaio Gaio

Reviewer Comments to Author:

The manuscript by Lopez-Garcia et al "Fungal and ciliate protozoa are the main rumen microbes associated with methane emissions in dairy cattle" describes a study where a large dataset of nanopore data was generated from rumen samples. It clearly describes the various steps that were taken to analyse the data. Various aspects of importance were taken into consideration such as abundance normalisation, and significance adjustment for multiple hypothesis testing. This study gives insights into previously unknown correlations between methane emission and taxa (particularly eukaryotic organisms), and between methane emission and genes.
Provided that some suggestions are implemented, this research paper should be considered for publication.
Minor comments:
Figures: almost all figures need some improvement:
All figures: axes titles font, and particularly, axes labels font needs to be larger.
All figures should be pdf/vectorised files. I (reviewer) see them all pixelated and I was not able to read most of the words.
Figure 1: this figure could be removed as it is little informative. If kept, a title on top of each panel could be added to highlight the different nature of the features.
Figure 2: 2A is little informative. I suggest keeping 2B, but visualized as a treemap instead, much more readable than a pie chart.
Figure 3: is there a message in this figure that has not already been made broadly explicit in the manuscript? If not, I suggest removing it or keep it as a supplementary figure.
A figure to report the core microbiome composition (bact, euk, and arch) as reported between line 131-138 would be more informative than the current figures 1,2A, and 3.
Figure 6: unreadable text right of 6A.
Figure 7: there are node fill colours that do not match the colours shown in the legend. Shouldn't the node fill colours be orange, green, grey (high, low, ns)?
Line 46: differentially abundant between low and high emission animals? Needs to be made explicit.
Line 124: RA acronym has not been introduced before
Line 143: What is the N50 of these reads? N50 and L50 could be reported.
Line 149: Could "most" here be made explicit in numbers (i.e.percentages)?
Line 150: Explicit what falls within cellular generic processes (or sub groups here of) in numbers (i.e.percentages)?
Line 155: PCA acronym first time appearance; needs introduction.

Line 170: I assume p-values in Table 1 have been adjusted for FDR with the BH method as described in the methods. If not, adjusted p-values should (also) be reported. Also, p-value adjustment method could be mentioned either here or in the Table caption.
Line 171-172: correlation is reported between parentheses when a statement about variance is made. Parentheses containing the correlation metric should be reported before the comma, while variance should be reported at the end of the sentence.
Line 179-181: very interesting findings!
Line 182-183: sentence structure needs revisiting.
Line 225-226: CLR "helps" but does not avoid compositional artefacts, and therefore it does not avoid spurious correlations.See Quinn et al (2021) "A Critique of Differential Abundance Analysis, and Advocacy for an Alternative" and his previous works
Line 291-293: sentence structure needs revisiting.
Line 296: As the methods section comes later, SqueezeMeta software needs citation and (possibly) a short intro.
Line 316: sentence structure needs revisiting.
Line 369-371 + 396: Good acknowledgement of limitations :)
Line 378: sentence structure needs revisiting.
Line 404: VFA acronym has been introduced once 200 lines earlier, could be just spelled out.
Line 426-431: Interesting hypotheses!
Line 484: I am not sure if the data from nanopore sequencing suffers much from the consequences of batch effects, but in other metagenomic sequencing techniques batch effects are unfortunately often cause of trouble. Here batches were used of 12 samples at a time per run. Was the batch effect controlled/tested for? Even though probably the samples were randomised (were they?), a batch effect could still be present. This is worth checking. A batch correction might even improve the signal!
Line 500: what package was used to assess sparsity?
Supplementary Table 1:p>0.05 hits should be included.

Methods

Are the methods appropriate to the aims of the study, are they well described, and are necessary controls included? Choose an item.

Conclusions

Are the conclusions adequately supported by the data shown? Choose an item.

Reporting Standards

Does the manuscript adhere to the journal’s guidelines on minimum standards of reporting? Choose an item.

Choose an item.

Statistics

Are you able to assess all statistics in the manuscript, including the appropriateness of statistical tests used? Choose an item.

Quality of Written English

Please indicate the quality of language in the manuscript: Choose an item.

Declaration of Competing Interests

Please complete a declaration of competing interests, considering the following questions:

- Have you in the past five years received reimbursements, fees, funding, or salary from an organisation that may in any way gain or lose financially from the publication of this manuscript, either now or in the future?
- Do you hold any stocks or shares in an organisation that may in any way gain or lose financially from the publication of this manuscript, either now or in the future?
- Do you hold or are you currently applying for any patents relating to the content of the manuscript?
- Have you received reimbursements, fees, funding, or salary from an organization that holds or has applied for patents relating to the content of the manuscript?
- Do you have any other financial competing interests?
- Do you have any non-financial competing interests in relation to this paper?

If you can answer no to all of the above, write 'I declare that I have no competing interests' below. If your reply is yes to any, please give details below.

I declare that I have no competing interests

I agree to the open peer review policy of the journal. I understand that my name will be included on my report to the authors and, if the manuscript is accepted for publication, my named report including any attachments I upload will be posted on the website along with the authors' responses. I agree for my report to be made available under an Open Access Creative Commons CC-BY license (http://creativecommons.org/licenses/by/4.0/). I understand that any comments which I do not wish to be included in my named report can be included as confidential comments to the editors, which will not be published.

Choose an item.

To further support our reviewers, we have joined with Publons, where you can gain additional credit to further highlight your hard work (see: https://publons.com/journal/530/gigascience). On publication of this paper, your review will be automatically added to Publons, you can then choose whether or not to claim your Publons credit. I understand this statement.

Yes Choose an item.
