# Supplementary material for: Fungal and ciliate protozoa are the main rumen microbes associated with methane emissions in dairy cattle
Source: Gigascience. 2022 Jan 25;11:giab088. doi: 10.1093/gigascience/giab088 (PMC8848325; doi:10.1093/gigascience/giab088)
Supplement: giab088_Reviewer_3_Report_Original_Submission — Wei Fan -- 9/13/2021 Reviewed [file giab088_reviewer_3_report_original_submission.pdf]

Reviewer Report

Title: Fungal and ciliate protozoa are the main rumen microbes associated with methane emissions in dairy cattle

Version: Original Submission **Date:** 9/13/2021

Reviewer name: Wei Fan

Reviewer Comments to Author:

This study has generated the largest ONT ruminal metagenomic dataset currently available, and found that ciliates and fungi are closely associated with methane emission. The findings is novel and have wide interest, but the manuscript need revision to be a good manuscript.
1. In all the Figures, the text are not clear. For me, I can't distinguish most of the characters, words, so it must be resolved.
2. In the Abstract Part:
"Background: This study analyzed whole rumen metagenome using long reads and considering its compositional nature in order to disentangle the role of rumen microbes in methane emissions." Is this really background? I can't see any backgroud and logic.
Methods: Line 33-38, can be removed from Abstract. Abstract needs concise, methods is not neccessary here.
3. In the Results Part:
"3.1 Cohort description
Our cohort included 437 Holstein lactating cows sampled at 14 different herds from northern Spain (Cantabria, Euskadi, Navarra and Girona regions)." This paragraph is very short and only have one sentence. I suggest to put it into methods part, and remove it from the results part. It can be merged with Methods 6.1 "Animal housing and feeding".
"3.7 Interaction networks" and "3.8 Taxonomy of genes" do not like sub-title names, they should be revised, to focus on the novel discoveries, which be more meaningful for the readers.
4. The Discussion Part:
The biggest problem is that it is too long. I suggest either make them concise, or move some into the results part. Those disscussion contents that are closely with the results can be merged into the results part, and further disscusions can be kept in the Disscussion part.
5. In the Methods Part:
"6.6 Bioinformatics" this sub-title is not meaningful, better change it to "Reads filtering, taxonomic and functional assignment" or others.
6. The last suggestion: try to assemble the ONT long reads, generate gene set, and chromosome results by binning or other technologies. Maybe this will get a lot more useful sequence data.

Methods

Are the methods appropriate to the aims of the study, are they well described, and are necessary controls included? Choose an item.

Conclusions

Are the conclusions adequately supported by the data shown? Choose an item.

Reporting Standards

Does the manuscript adhere to the journal's guidelines on minimum standards of reporting? Choose an item.

Choose an item.

Statistics

Are you able to assess all statistics in the manuscript, including the appropriateness of statistical tests used? Choose an item.

Quality of Written English

Please indicate the quality of language in the manuscript: Choose an item.

Declaration of Competing Interests

Please complete a declaration of competing interests, considering the following questions:

- Have you in the past five years received reimbursements, fees, funding, or salary from an organisation that may in any way gain or lose financially from the publication of this manuscript, either now or in the future?
- Do you hold any stocks or shares in an organisation that may in any way gain or lose financially from the publication of this manuscript, either now or in the future?
- Do you hold or are you currently applying for any patents relating to the content of the manuscript?
- Have you received reimbursements, fees, funding, or salary from an organization that holds or has applied for patents relating to the content of the manuscript?
- Do you have any other financial competing interests?
- Do you have any non-financial competing interests in relation to this paper?

If you can answer no to all of the above, write 'I declare that I have no competing interests' below. If your reply is yes to any, please give details below.

I declare that I have no competing interests

I agree to the open peer review policy of the journal. I understand that my name will be included on my report to the authors and, if the manuscript is accepted for publication, my named report including any attachments I upload will be posted on the website along with the authors' responses. I agree for my

report to be made available under an Open Access Creative Commons CC-BY license (http://creativecommons.org/licenses/by/4.0/). I understand that any comments which I do not wish to be included in my named report can be included as confidential comments to the editors, which will not be published.

Choose an item.

To further support our reviewers, we have joined with Publons, where you can gain additional credit to further highlight your hard work (see: https://publons.com/journal/530/gigascience). On publication of this paper, your review will be automatically added to Publons, you can then choose whether or not to claim your Publons credit. I understand this statement.

Yes Choose an item.
